# Maturation of Human Induced Pluripotent Stem Cell-Derived Cardiomyocytes by Soluble Factors from Human Mesenchymal Stem Cells

**DOI:** 10.1016/j.ymthe.2018.08.012

**Published:** 2018-08-16

**Authors:** Shohei Yoshida, Shigeru Miyagawa, Satsuki Fukushima, Takuji Kawamura, Noriyuki Kashiyama, Fumiya Ohashi, Toshihiko Toyofuku, Koichi Toda, Yoshiki Sawa

**Affiliations:** 1 Department of Cardiovascular Surgery, Osaka University Graduate School of Medicine, 2-2 Yamadaoka, Suita, Osaka 565-0871, Japan; 2 Department of Immunology and Regenerative Medicine, Osaka University Graduate School of Medicine, 2-2 Yamadaoka, Suita, Osaka 565-0871, Japan

**Keywords:** maturation of cardiomyocytes, induced pluripotent stem cells, mesenchymal stem cells

## Abstract

In this study, we proposed that the functionality or phenotype of differentiated cardiomyocytes derived from human induced pluripotent stem cells (iPSC-CMs) might be modified by co-culture with mesenchymal stem cells (MSCs), resulting in an improved therapeutic potential for failing myocardial tissues. Structural, motility, electrophysiological, and metabolic analyses revealed that iPSC-CMs co-cultured with MSCs displayed aligned myofibrils with A-, H-, and I-bands that could contract and relax quickly, indicating the promotion of differentiation and the establishment of the iPSC-CM structural framework, and showed clear gap junctions and an electric pacing of >2 Hz, indicating enhanced cell-cell interactions. In addition, soluble factors excreted by MSCs, including several cytokines and exosomes, enhanced cardiomyocyte-specific marker production, produced more energy under normal and stressed conditions, and reduced reactive oxygen species production by iPSC-CMs under stressed condition. Notably, gene ontology and pathway analysis revealed that microRNAs and proteins in the exosomes impacted the functionality and maturation of iPSC-CMs. Furthermore, cell sheets consisting of a mixture of iPSC-CMs and MSCs showed longer survival and enhanced therapeutic effects compared with those consisting of iPSC-CMs alone. This may lead to a new type of iPSC-based cardiomyogenesis therapy for patients with heart failure.

## Graphical Abstract

**Figure:**
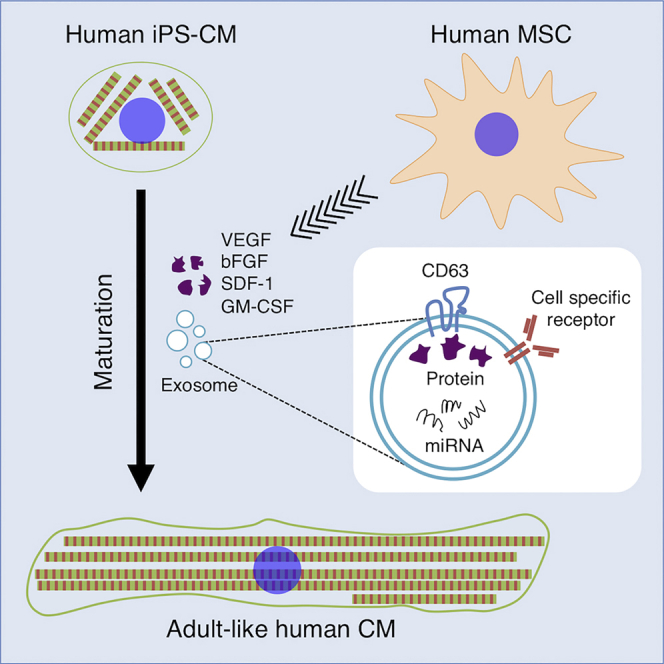

## Teaser

Maturation of cardiomyocytes derived from human induced pluripotent stem cells was promoted by soluble factors secreted from mesenchymal stem cells, such as several cytokines and exosomes, enhancing therapeutic potential for failing myocardial tissues. This may lead to a new type of iPSC-based cardiomyogenesis therapy for patients with heart failure.

## Introduction

Heart failure retains a high global mortality rate, despite marked progress in medical treatments; therefore, it is vital to apply new concepts for developing novel therapeutic alternatives.^1^, ^2^ In the past decade, several stem cell therapies including bone marrow progenitor cells, cardiac cells, and somatic stem cells have been explored in clinical settings.^3^, ^4^, ^5^, ^6^, ^7^ Unfortunately, their therapeutic effects are limited to the specific region of the heart or to the responding patient, likely because these depend primarily on paracrine effects by the transplanted cells and not on the recovery of functioning cardiomyocytes. Recently, a cardiomyogenesis therapy using cardiomyocytes derived from human induced pluripotent stem cells (hiPSC-CMs) was proposed as a new, alternative candidate therapeutic treatment for several previous stem cell applications.^8^, ^9^, ^10^ However, remaining concerns including poor cell survival or immature cardiomyogenic differentiation, which directly influence therapeutic effects, limit the efficacy of clinical applications.^8^, ^9^, ^11^ Conversely, it was reported that hiPSC-derived mature cardiac tissue showed longer survival after orthotopic transplantation.^12^ Therefore, strategies to support the transplanted hiPSC-CMs by enhancing their maturity and functionality must be identified.

Several maturation protocols described previously showed that hiPSC-CMs were not matured completely compared with cardiomyocytes in adult hearts, and that substantial time was required to mature the hiPSC-CMs.^13^, ^14^, ^15^, ^16^ Moreover, Yang et al.^14^ described the limitations of using a single factor to induce a complex trait such as maturation. In contrast, human mesenchymal stem cells (hMSCs) have been shown to secrete several soluble factors, which promote the differentiation of other stem or progenitor cells such as neural stem cells or oligodendroglial progenitor cells, and enhance the electrical coupling of hiPSC-CMs.^17^, ^18^, ^19^, ^20^ On the other hand, one potential reason for poor cell survival may constitute a damaged vascular network at the implanted site.^21^, ^22^ Somatic stem cells, such as myoblasts, co-cultured with hMSCs have been reported to enhance cell survival by forming a rich vascular network induced by cytokine secretion; hMSCs can also behave as feeder cells to support co-cultured stem or progenitor cell survival, proliferation, and differentiation.^23^, ^24^, ^25^

Herein, we hypothesized that hMSC co-culture might modulate hiPSC-CM maturity and functionality *in vitro* and enhance their cell survival and therapeutic potential for treating heart failure following myocardial infarction *in vivo*. We also investigated whether soluble factors secreted from hMSCs could induce hiPSC-CM maturation, and whether co-culture and co-transplantation with hMSCs could enhance hiPSC-CM cell survival and therapeutic effects.

## Results

### hMSCs Increase the Cardiac Troponin T-Positive Cell Population and Promote hiPSC-CM Molecular Development

**Figure 1:**
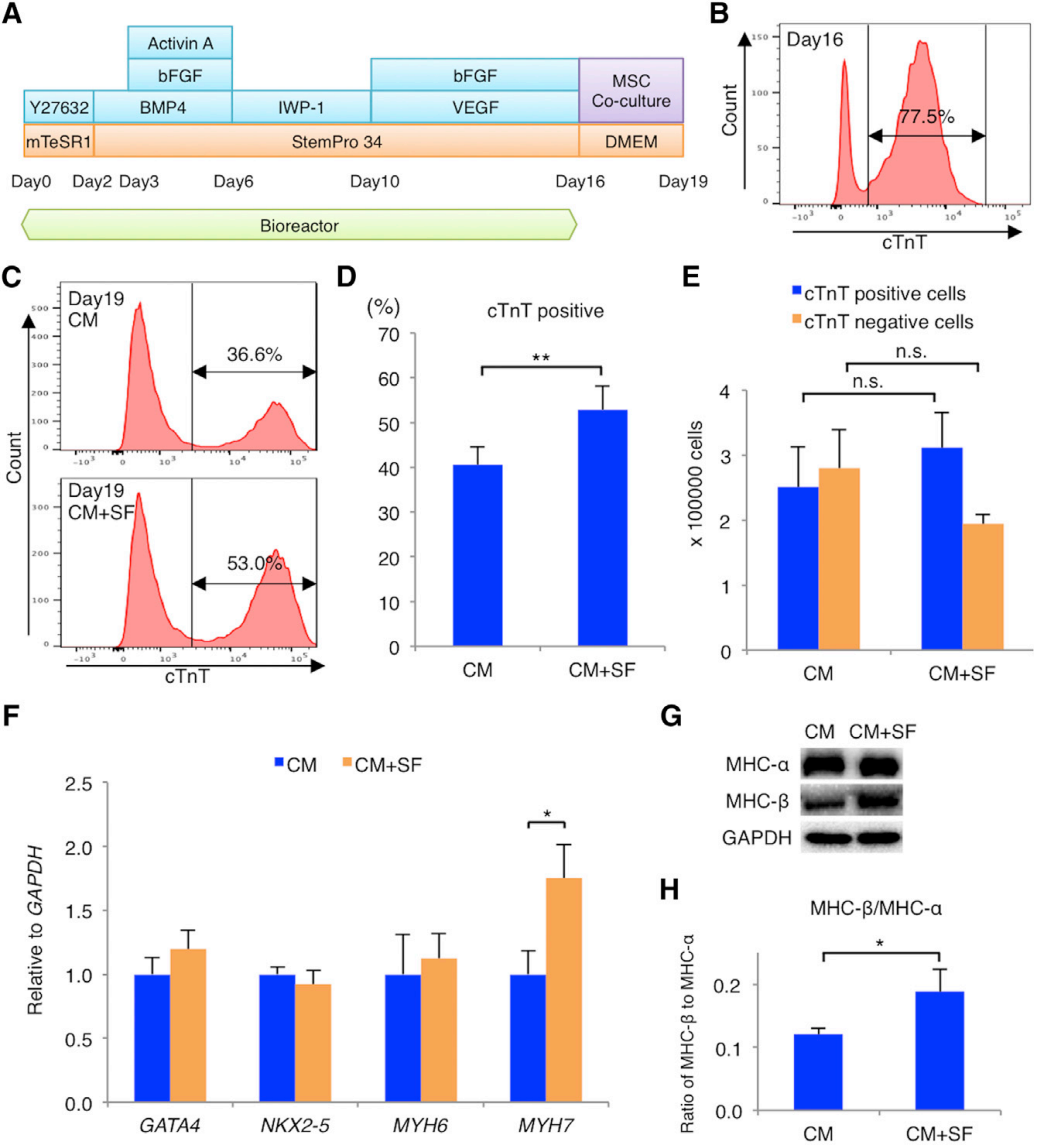
Human Mesenchymal Stem Cells Increase the Population of Cardiac Troponin T-Positive Cells and Promote the Molecular Development of Cardiomyocytes Derived from Human Induced Pluripotent Stem Cells (A) Cardiomyogenic differentiation protocol and co-culture with human mesenchymal stem cells (hMSCs). (B) Representative flow cytometry data of differentiated human induced pluripotent stem cells (hiPSC-CMs) stained with anti-cardiac troponin T (cTnT) antibodies at day 16. (C) Representative flow cytometry data of differentiated hiPSC-CMs with and without hMSC-derived soluble factors stained with anti-cTnT antibodies at day 19 (CM+SF and CM, respectively). (D) Percentage of cTnT-positive cells in the CM and CM+SF groups as determined by flow cytometry (n = 5 for each group). **p < 0.01, Student t test. (E) Number of cTnT-positive or -negative cells in the CM and CM+SF groups (n = 3 for each group). n.s., not significant, Student t test. (F) Expression of cardiac cell-specific genes (GATA binding protein 4 [*GATA4*], NK2 homeobox 5 [*NKX2-5*], and myosin heavy chain 6 [*MYH6*] and *MYH7*) in CM or CM+SF cells, normalized against *GAPDH* expression (n = 7 for each group). *p < 0.05, Student t test. (G) Western blot of CM or CM+SF cells using anti-myosin heavy chain alpha (MHC-α) antibody, anti-MHC-β antibody, and anti-GAPDH antibodies. (H) Ratio of MHC-β to MHC-α in CM or CM+SF cells as determined by western blotting (n = 4 for each group). *p < 0.05, Student t test. For all experiments, results are shown as mean + SEM. bFGF, basic fibroblast growth factor; BMP4, bone morphogenetic protein 4; VEGF, vascular endothelial growth factor.

hiPSCs were differentiated into cardiomyocytes using the protocol described in the Materials and Methods (Figure 1A). The obtained cardiomyocyte purity was 76% ± 3%, as assessed by flow cytometry for cardiac troponin T (cTnT) (Figure 1B). After the cardiac differentiation, hiPSC-CMs were cultured alone (CM), or co-cultured with hMSCs (CM+MSC) or hMSC-derived soluble factors (CM+SF) for 3 days in new plate dish, as described in the Materials and Methods (Figure S1). Although the suspension culture using bioreactors did not require a high number of adherent cells, a 3-day plate culture of hiPSC-CMs after the suspension culture increased the number of adherent cells such as fibroblasts, leading to a decrease in the purity of cardiomyocytes. Nevertheless, the cTnT-positive cell proportion was significantly higher in the CM+SF (53% ± 5%) than in the CM group (40% ± 4%; p = 0.0013) (Figures 1C and 1D). In the CM+SF and CM groups, total cell numbers were similar, whereas the cTnT-positive cell number was slightly, albeit not significantly, higher (3.1 ± 0.5 × 10^6^ versus 2.5 ± 0.6 × 10^6^ cells; p = 0.2603) and the cTnT-negative cell number (1.9 ± 0.2 × 10^6^ versus 2.8 ± 0.6 × 10^6^ cells; p = 0.2571) was slightly lower in the CM+SF than in the CM group (Figure 1E). qRT-PCR analysis (described in the Supplemental Materials and Methods) of known cardiac markers further validated maturation enhancement by co-culture with hMSC soluble factors. The mRNA expression of transcriptional regulators, such as GATA binding protein 4 (*GATA4*) and NK2 homeobox 5 (*NKX2-5*), did not differ significantly between groups. However, hMSC-secreted soluble factors significantly increased relative myosin heavy chain 7 (*MYH7*) mRNA expression (p = 0.0272), whereas that of myosin heavy chain 6 (*MYH6*) did not differ significantly between groups (Figure 1F). Notably, western blotting analysis (described in the Supplemental Materials and Methods) revealed that the myosin heavy chain-β (MHC-β)-to-MHC-α ratio, a known cardiac maturation marker,^11^ was significantly higher in the CM+SF (0.19 ± 0.04) group than the CM group (0.12 ± 0.01; p = 0.0455) (Figures 1G and 1H), consistent with the result of mRNA expression analysis. Thus, hiPSC-CMs induced differentiation more effectively with than without hMSC co-cultivation.

### hMSCs Promote hiPSC-CM Structural Development

**Figure 2:**
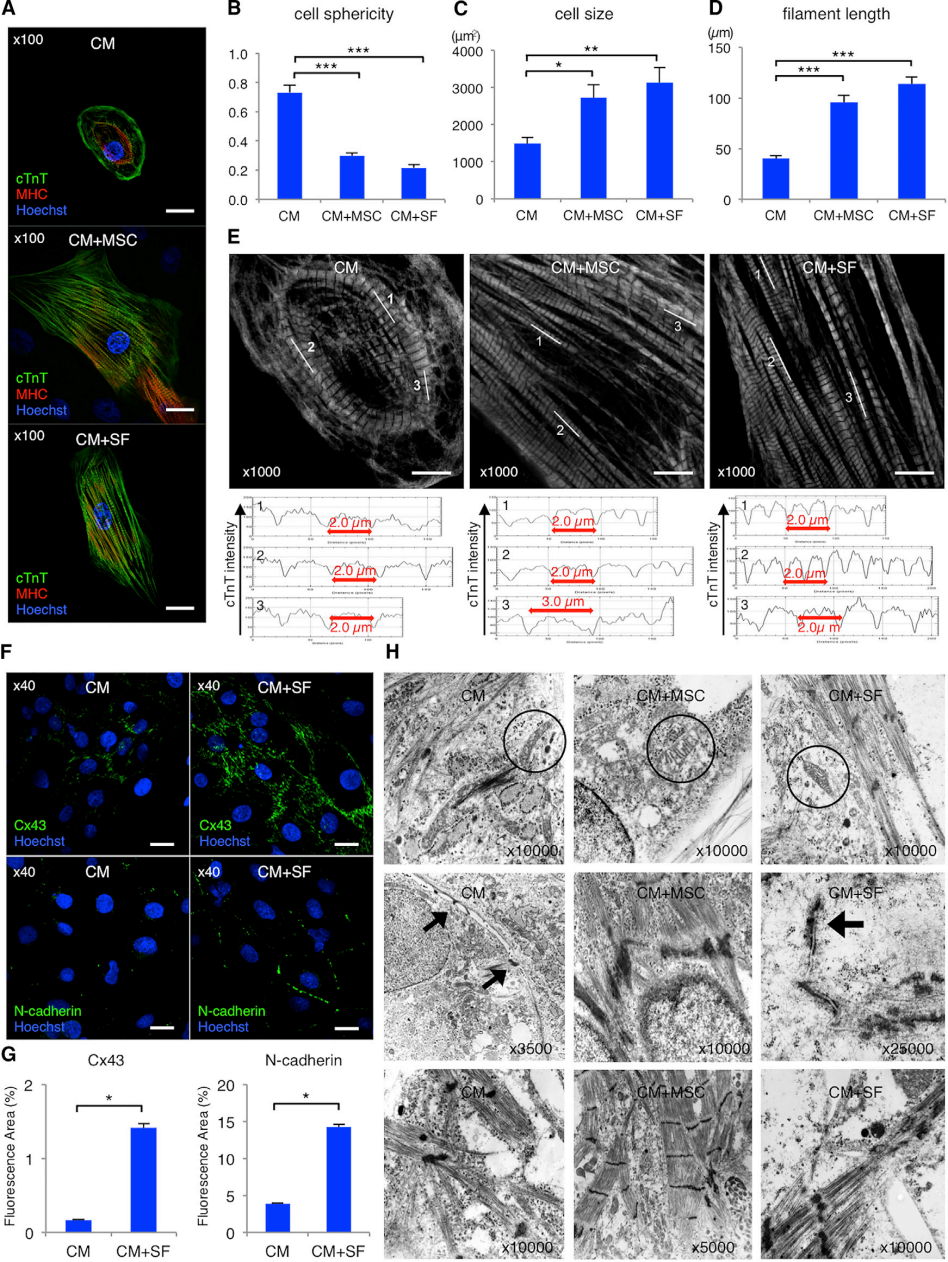
hMSCs Promote Structural Development in hiPSC-CMs (A) Immunohistochemistry of cardiac troponin T (cTnT; green), myosin heavy chain (MHC; red), and nuclei (Hoechst33258; blue) in differentiated cardiomyocytes (CM), cardiomyocytes co-cultured with mesenchymal stem cells (CM+MSC), and cardiomyocytes cultured with MSC-derived soluble factors (CM+SF). Scale bars: 30 μm. (B–D) Cell sphericity (B), cell size (C), and filament length (D) in the CM, CM+MSC, and CM+SF groups (n = 7 for each group). *p < 0.05; **p < 0.01; ***p < 0.001, one-way ANOVA with post hoc Tukey’s honestly significant difference (HSD) test. (E) Upper panels display immunohistochemistry of cTnT (white) in the CM, CM+MSC, or CM+SF groups through super-resolution microscopy. Lower panels show the intensity of cTnT at the white lines in the above images. Scale bars: 10 μm. (F) Upper panels show immunohistochemistry of connexin 43 (Cx43; green) and Hoechst33258 (blue) in the CM and CM+SF groups. Lower panels show immunohistochemistry of N-cadherin (green) and nuclei (Hoechst33258; blue) in the CM and CM+SF groups. Scale bars: 20 μm. (G) Percent of fluorescence area, which was stained with Cx43 and N-cadherin, in the CM and CM+SF groups (n = 4 for each group). *p < 0.05, Student t test. (H) Transmission electron microscopy images of cardiomyocytes in the CM, CM+MSC, and CM+SF groups. For all experiments, results are shown as mean + SEM.

To evaluate the presence of cardiac-specific components in hiPSC-CMs, we performed immunostaining (detailed in the Supplemental Materials and Methods). Differentiated cardiomyocytes in the CM, CM+MSC, and CM+SF groups were stained with cTnT (green), cardiac MHC (red), and nuclei (Hoechst 33342; blue) (Figure 2A). The CM group (0.73 ± 0.05) exhibited a significantly higher sphericity index than the CM+MSC (0.30 ± 0.02; p < 0.0001) and CM+SF groups (0.22 ± 0.02, p < 0.0001; ANOVA: p < 0.0001) (Figure 2B), but a significantly lower average cell size (1,483 ± 496 versus 2,720 ± 955 μm^2^, p = 0.0327 [CM+MSC], and 3,138 ± 1,034 μm^2^, p = 0.0042 [CM+SF]; ANOVA: p = 0.0037) (Figure 2C). The filament length was also significantly shorter in the CM (40 ± 9 μm) than in the CM+MSC (96 ± 18 μm; p < 0.0001) and CM+SF groups (114 ± 18 μm; p < 0.0001) (Figure 2D). Super-resolution microscopic images demonstrated that CM group sarcomeres had an average length of 2.0 μm and did not contain H-bands (Figure 2E), whereas CM+MSC group sarcomeres had the same or greater lengths and contained H-bands, and CM+SF group sarcomeres exhibited 2.0-μm average length in addition to H-bands. These findings indicated that hMSC-derived soluble factors and cell-cell contact with hMSCs might contribute to hiPSC-CM structural alternations.

Connexin 43 (green) or N-cadherin (green) and nuclei (Hoechst 33342; blue) staining images showed higher connexin 43 or N-cadherin expression in the CM+SF group (1.4% ± 0.1% and 14.2% ± 0.3%, respectively) than in the CM group (0.2% ± 0.0%, p = 0.0495, and 3.9% ± 0.1%, p = 0.0495) (Figures 2F and 2G), which might lead to a robust physical and electrical junction in the hiPSC-CMs.

Moreover, transmission electron microscopy (TEM) images of hiPSC-CMs in the CM group showed intersecting immature myofibrils without A- or I-bands, small narrow mitochondria with indistinct cristae, and poor adhesion at the intercellular junction. Conversely, the CM+SF group showed myofibrils with immature A- and I-bands, mitochondria with distinct cristae, and gap junctions and intercalated disks at the intercellular junction. The CM+MSC group showed aligned myofibrils with clear A- and I-bands, mitochondria with more distinct cristae, and a high density of intercalated disks to which actin filaments attached (Figure 2H). Thus, structural analyses indicated that co-cultured hMSCs enhanced hiPSC-CM maturation.

### hMSCs Promote hiPSC-CM Motility

**Figure 3:**
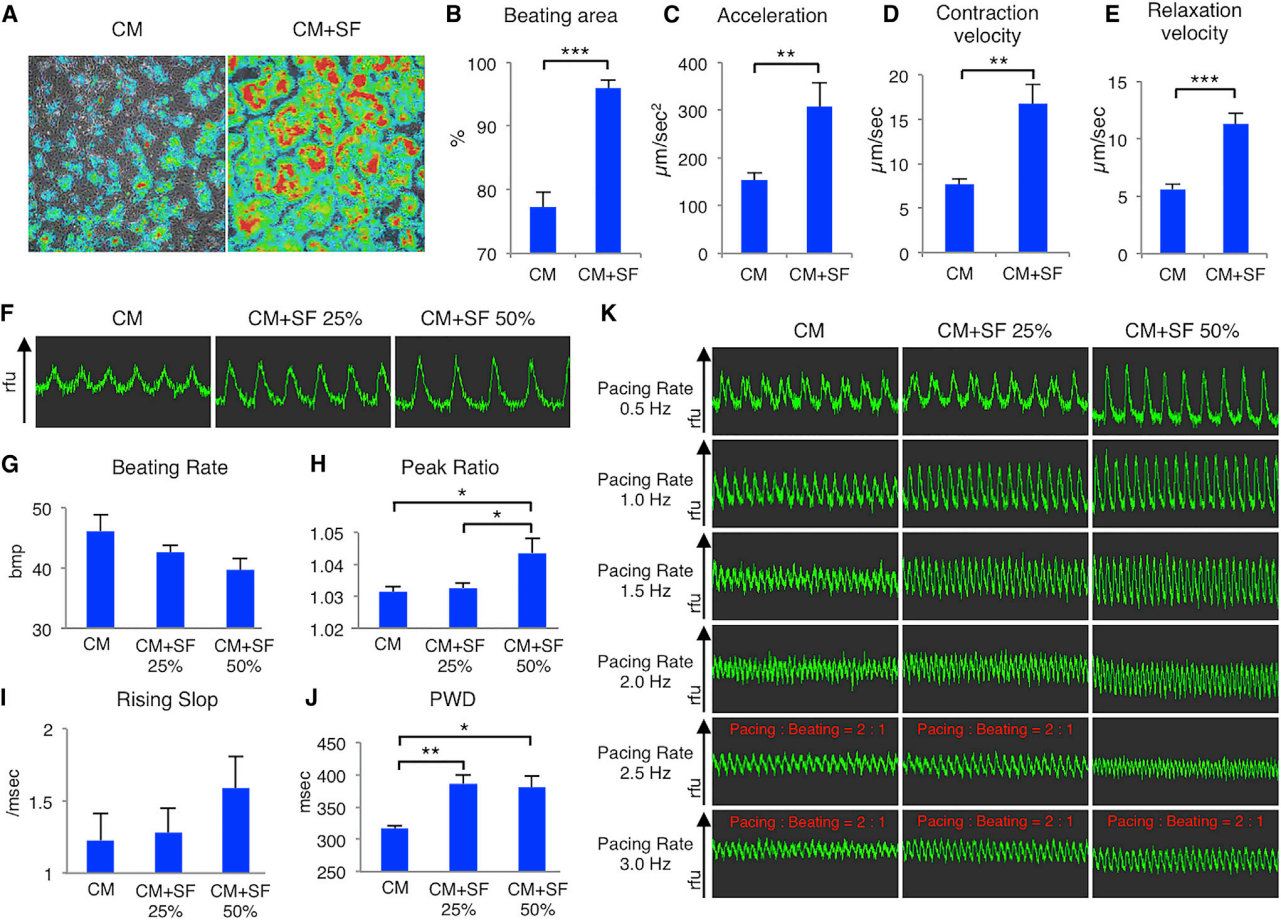
hMSCs Promote Contractile and Electrophysiological Development in hiPSC-CMs (A) Representative velocity data in differentiated cardiomyocytes (CM; left panel) and cardiomyocytes cultured with MSC-derived soluble factors (CM+SF; right panel) by a motion analysis system. Red and blue represent high and low velocity, respectively. (B–E) Percentage of beating area (B), acceleration (C), contraction velocity (D), and relaxation velocity (E) in the CM and CM+SF groups (n = 4 for each group). **p < 0.01; ***p < 0.001, Student t test. (F) Representative wave forms associated with Ca^2+^ transients in CM, CM with soluble factors secreted from 25% total percentage hMSCs (CM+SF 25%), or CM with soluble factors secreted from 50% total percentage hMSCs (CM+SF 50%), using the FDSS/μCELL system. (G–J) Beating rate (G), peak ratio (H), rising slope (I), or peak width duration (PWD) (J) of cells in the CM, CM+SF 25%, and CM+SF 50% groups as analyzed by FDSS software U8524-12 (n = 6 for each group). *p < 0.05; **p < 0.01, one-way ANOVA with post hoc Tukey’s HSD test. (K) Representative wave forms associated with Ca^2+^ transients in CM, CM+SF 25%, and CM+SF 50% cells with a pacing rate of 0.5, 1, 1.5, 2, 2.5, or 3 Hz. For all experiments, results are shown as mean + SEM.

To evaluate hiPSC-CM contractility, we performed motion analysis, in which the high-velocity area is red and low-velocity area is blue (Figure 3A; Videos S1, S2, S3, and S4). The CM+SF group had a significantly larger beating area (96% ± 1%) than the CM group (77% ± 2%; p < 0.0001) (Figure 3B). Other CM+SF parameters such as acceleration (307 ± 50 versus 153 ± 15 μm/s2; p = 0.0079), contraction velocity (16.8 ± 2.2 versus 7.7 ± 0.6 μm/s; p = 0.0027), and relaxation velocity (11.3 ± 0.9 versus 5.6 ± 0.4 μm/s; p < 0.0001) were also significantly higher than those in the CM group (Figures 3C–3E). Thus, hiPSC-CMs co-cultured with hMSCs showed increased contractility.

Video S1. Representative Video of hiPSC-CMs in the CM Group without Color

Video S2. Representative Video of hiPSC-CMs in the CM Group with Color

Video S3. Representative Video of hiPSC-CMs in the CM+SF Group without Color

Video S4. Representative Video of hiPSC-CMs in the CM+SF Group with Color

### hMSCs Promote hiPSC-CM Electrophysiological Development

To further investigate the mechanisms underlying the cardiomyocyte performance changes, we performed an intracellular calcium ratiometric dye fluo-8 assay (Figure 3F). The CM+SF 25% (43 ± 1 beats per minute [bpm]) and 50% groups (40 ± 2 bpm) beating rates were slightly lower than the CM group rate (46 ± 3 bpm) (ANOVA: p = 0.1258) (Figure 3G). The CM+SF 50% group had a significantly higher peak ratio (1.044 ± 0.004) than the CM+SF 25% group (1.033 ± 0.001; p = 0.0208) and the CM group (1.031 ± 0.002, p = 0.0170; ANOVA: p = 0.0289) (Figure 3H). The rising slope in the CM+SF 50% (1.6 ± 0.2/ms) group was not significantly higher than that in the CM (1.2 ± 0.2/ms) or CM+SF 25% group (1.3 ± 0.2/ms; ANOVA: p = 0.4904) (Figure 3I). The CM group showed significantly shorter peak width duration (PWD; 317 ± 4 ms) than the CM+SF 25% (386 ± 13 ms; p = 0.0098) and 50% groups (380 ± 18 ms, p = 0.0256; ANOVA: p = 0.0085) (Figure 3J). Additionally, although CM and CM+SF 25% group cardiomyocytes could not achieve an electrical pacing of >2 Hz, CM+SF 50% group cardiomyocytes showed 2.5-Hz electric pacing (Figure 3K). Thus, co-culture with hMSCs promoted electrophysiological development in hiPSC-CMs.

### hMSCs Promote hiPSC-CM Metabolic Development

**Figure 4:**
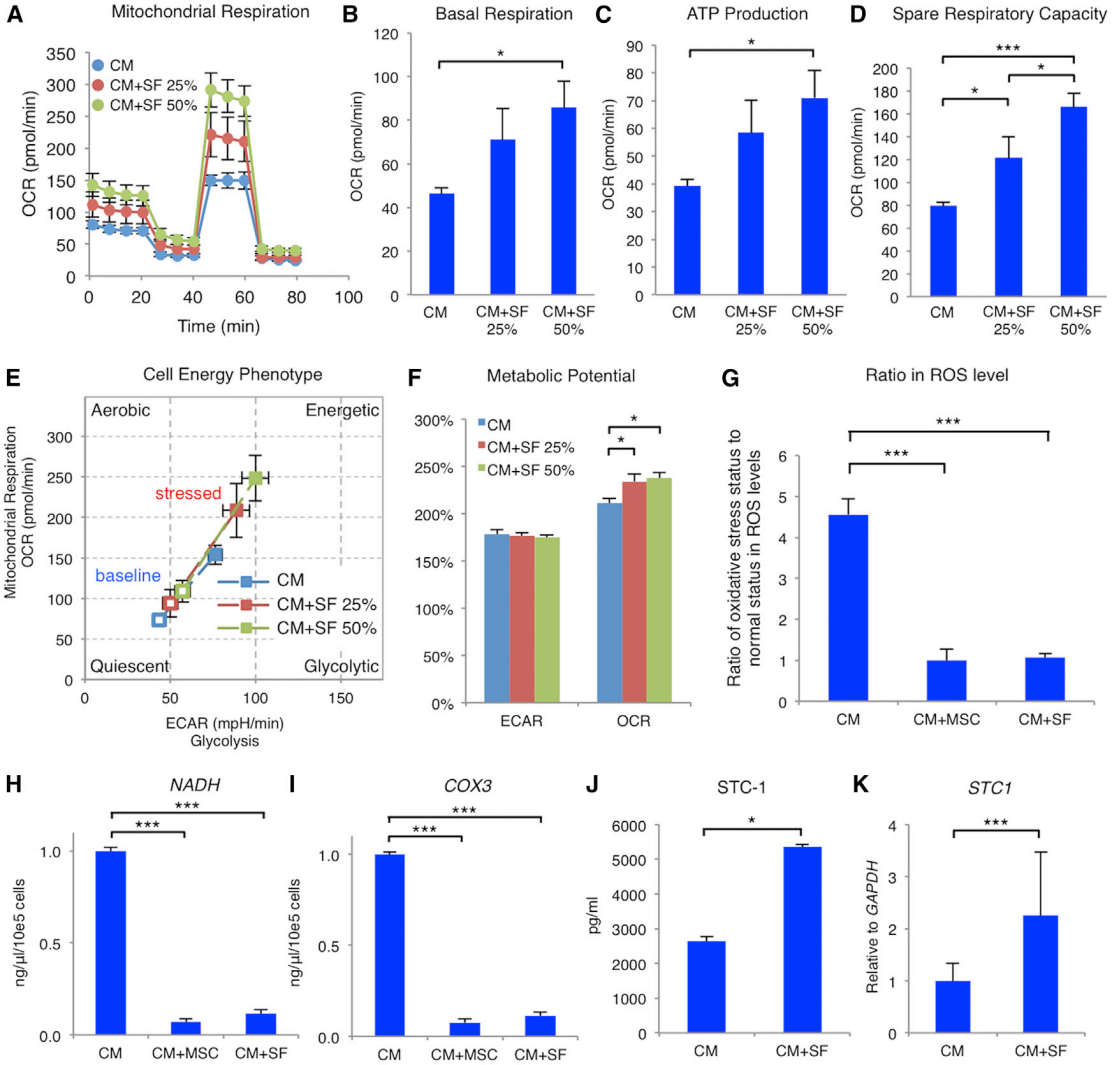
hMSCs Promote Metabolic Development in hiPSC-CMs (A) Representative mitochondrial respiration in differentiated cardiomyocytes (CM), CM with soluble factors secreted from 25% of hMSCs (CM+SF 25%), or CM with soluble factors secreted from 50% of hMSCs (CM+SF 50%) after incubation with the ATP synthase inhibitor oligomycin, the respiratory uncoupler carbonyl cyanide-p-trifluoromethoxyphenylhydrazone (FCCP), and the respiratory chain blockers rotenone and antimycin A. (B–D) Basal respiration (B), ATP production (C), and spare respiratory capacity (D) of cells in the CM, CM+SF 25%, and CM+SF 50% groups (n = 9 for each group). *p < 0.05; ***p < 0.001, one-way ANOVA with post hoc Tukey’s HSD test. (E) Cell energy phenotype of cells in the CM, CM+SF 25%, and CM+SF 50% groups under normal (outlined shapes) and stressed (solid shapes) conditions (n = 9 for each group). (F) Metabolic potential of glycolysis or mitochondrial respiration in the CM, CM+SF 25%, and CM+SF 50% groups (n = 9 for each group). *p < 0.05. (G) Ratio of reactive oxygen species (ROS) levels in cells undergoing oxidative stress compared with normal cells in the CM, CM+MSC, and CM+SF groups (n = 7 for each group). ***p < 0.001, one-way ANOVA with post hoc Tukey’s HSD test. (H and I) Quantitative analysis of mitochondrial genes (*NADH*, H; *COX3*, I) from media containing CM, CM+MSC, or CM+SF cell culture (n = 4 for each group). ***p < 0.001, one-way ANOVA with post hoc Tukey’s HSD test. (J) Concentration of stanniocalcin 1 (STC-1) in media containing CM or CM+SF cell culture (n = 3 for each group). *p < 0.05, Student t test. (K) Expression of the *STC1* gene in the CM and CM+SF groups, normalized against *GAPDH* expression (n = 7 for each group). ***p < 0.001, Student t test. For all experiments, results are shown as mean + SEM. ECAR, extracellular acidification rate; OCR, oxygen consumption rate.

hMSC-derived soluble factors increased the hiPSC-CM oxygen consumption rate (OCR) at every phase in a dose-dependent manner (Figure 4A). The CM+SF 50% group had significantly higher basal respiration (85.6 ± 12.5 pmol/min) and ATP production (70.9 ± 10.0 pmol/min) than the CM group (46.1 ± 3.0 pmol/min, p = 0.0205, and 39.2 ± 2.4 pmol/min, p = 0.0229, respectively), whereas basal respiration and ATP production in the CM+SF 25% group (71.1 ± 14.2 pmol/min, p = 0.1151, and 58.2 ± 11.8 pmol/min, p = 0.1405) were similar to those in the CM group (Figures 4B and 4C). Moreover, the CM+SF 50% (165.6 ± 12.3 pmol/min; p = 0.0002) and 25% groups (121.0 ± 18.8 pmol/min; p = 0.0340) had significantly higher spare respiratory capacity than the CM group (79.3 ± 3.4 pmol/min) (Figure 4D). Figure 4E shows cellular energy phenotypes of the CM, CM+SF 25%, and CM+SF 50% groups under normal and stressed conditions. Although extracellular acidification rate (ECAR) metabolic potentials did not differ between the three groups (CM: 164% ± 4%; CM+SF 25%: 166% ± 4%; CM+SF 50%: 161% ± 4%; ANOVA: p = 0.6325), the CM group OCR metabolic potential (211% ± 6%) was significantly lower than those of the CM+SF 25% (234% ± 7%; p = 0.0463) and CM+SF 50% groups (238% ± 7%, p = 0.0189; ANOVA: p = 0.0137) (Figure 4F). Thus, hMSC-derived soluble factors enhanced hiPSC-CM mitochondrial energetics.

In addition, oxidative stress increased CM group reactive oxygen species (ROS) levels to 4.6 ± 0.4-fold more than those of normal culture conditions. Conversely, ROS levels were unchanged in the CM+MSC (1.0 ± 0.3-fold increase; p < 0.0001) and the CM+SF (1.1 ± 0.1-fold increase; p < 0.0001) groups under oxidative stress (ANOVA: p < 0.0001) (Figure 4G). Consistent with the release of mitochondrial DNA fragments into the media upon mitochondrial damage, the relative levels of mitochondrial genes, such as *NADH* and *COX3*, were significantly increased in the CM group compared with those of the CM+MSC and CM+SF groups (*NADH*: 1.00 ± 0.02 versus 0.07 ± 0.02, p < 0.0001, versus 0.07 ± 0.02, p < 0.0001; *COX3*: 1.00 ± 0.01 versus 0.08 ± 0.02, p < 0.0001, versus 0.08 ± 0.02, p < 0.0001; ANOVA: p < 0.0001) (Figures 4H and 4I). Stanniocalcin 1 (STC-1), a protein that stabilizes mitochondrial membrane potential, was significantly increased in the medium of the CM+ SF group (5,354 ± 75 pg/mL) compared with that in the CM group (2,632 ± 133 pg/mL; p = 0.0478), suggestive of ROS production suppression (Figure 4J). Furthermore, qRT-PCR revealed significantly higher relative hiPSC-CM *STC-1* mRNA expression in the CM+SF group (2.3 ± 1.2; p = 0.0004) than in the CM group (1.0 ± 0.3), suggesting that hMSC-derived soluble factors increased *STC-1* mRNA expression in hiPSC-CMs (Figure 4K). Thus, hiPSC-CMs co-cultured with hMSCs were protected from mitochondria-derived ROS production.

### hMSC-Derived Soluble Factors

**Figure 5:**
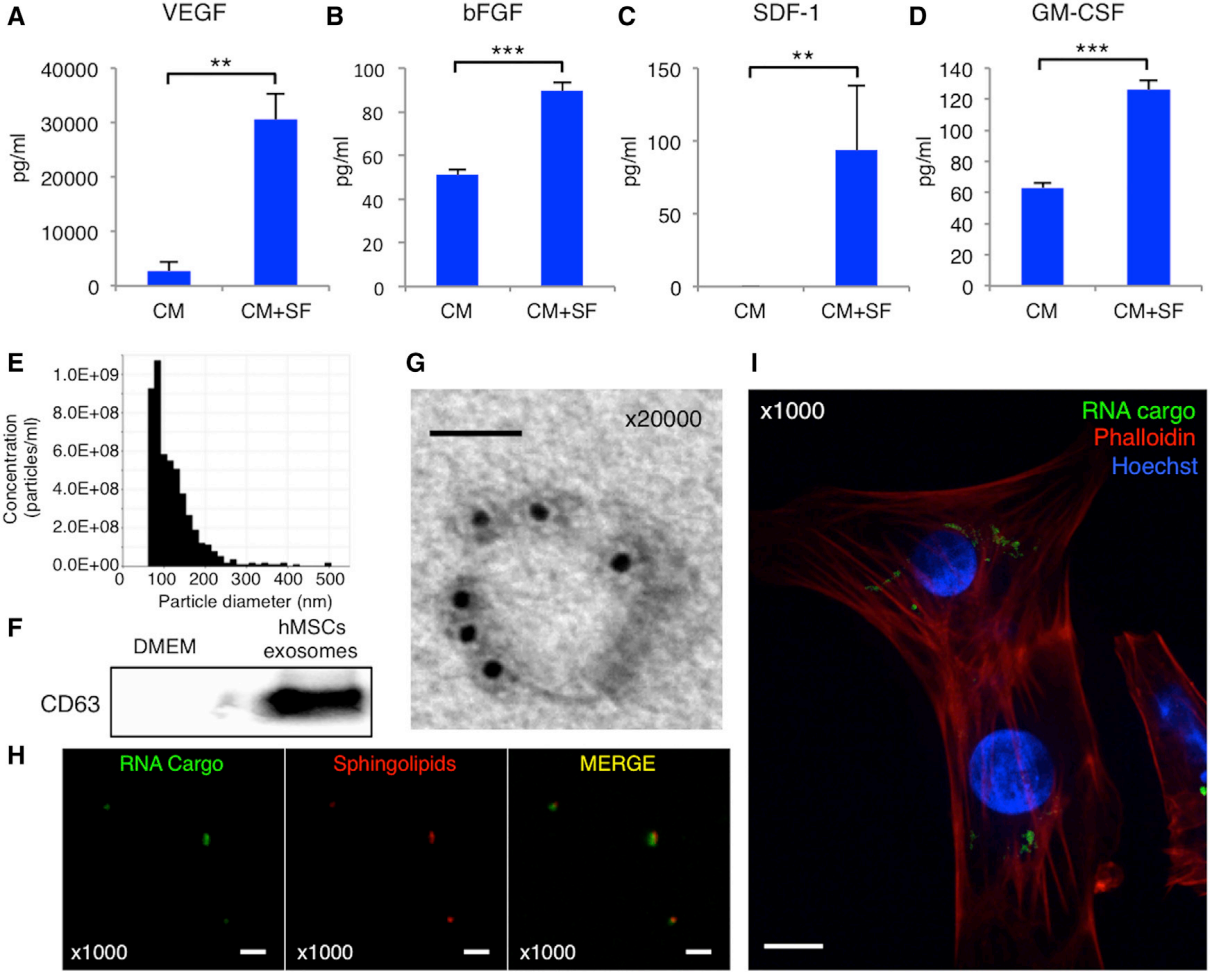
Soluble Factors Derived from hMSCs (A–D) Concentration of vascular endothelial growth factor (VEGF) (A), basic fibroblast growth factor (bFGF) (B), stromal cell-derived factor (SDF-1) (C), and granulocyte-macrophage colony-stimulating factor (GM-CSF) (D) in differentiated cardiomyocytes (CM) and cardiomyocytes cultured with MSC-derived soluble factors (CM+SF; n = 8 for each group). **p < 0.01; ***p < 0.001, Student t test. (E) Distribution of particle size after ultracentrifugation of media with cultured hMSCs using the qNano system. (F) Representative western blotting data of the exosomes of hMSCs using anti-CD63 antibody. (G) Transmission electron microscopy images of an exosome derived from hMSCs stained with anti-CD63 antibody. Scale bar: 50 nm. (H) RNA (stained green, left) and sphingolipids (stained red, middle) are shown; particles containing both (right) RNA and sphingolipids were considered to be exosomes derived from hMSCs. Scale bars, 2 μm. (I) Exosomes derived from hMSCs stained with RNA cargo (green) were incubated with hiPSC-CMs stained with phalloidin (red) and nuclei (Hoechst33258; blue) 12 hr after addition of the exosomes into the culture media. Scale bar, 10 μm. For all experiments, results are shown as mean + SEM.

Next, we examined the mechanisms by which hMSC co-culture affected hiPSC-CMs. To assess hiPSC-CM maturation mechanisms, we investigated the hMSC-derived soluble factors. Cytokines in the supernatant were measured using Bio-Plex and an ELISA (Figure S2). The CM+SF group had higher vascular endothelial growth factor (VEGF; 30,566 ± 4755 pg/mL), basic fibroblast growth factor (bFGF; 89 ± 4 pg/mL), stromal cell-derived factor 1 (SDF-1; 94 ± 44 pg/mL), and granulocyte-macrophage colony-stimulating factor (GM-CSF; 126 ± 6 pg/mL) concentrations than the CM group (2,737 ± 1,664 pg/mL, p = 0.0016; 51 ± 2 pg/mL, p = 0.0008; 0 ± 0 pg/mL, p = 0.0027; 63 ± 3 pg/mL, p = 0.0008, respectively) (Figures 5A–5D). Additionally, investigation of hMSC-derived exosome contents showed that the majority of particles, which were concentrated using ultracentrifugation, had a diameter of 60–200 nm (Figure 5E) and were positive for CD63, which is specific to extracellular exosomes, by western blotting (Figure 5F). Following anti-CD63 antibody immunostaining, TEM images showed CD63-positive particles with 120-nm diameter (Figure 5G). Therefore, we considered the particles to be exosomes in the following assay. Super-resolution microscopic images revealed that the exosomes have sphingolipids and RNA (Figure 5H). Exosomes, in which the RNA cargo was stained green, were added to culture media containing hiPSC-CMs. Histological analysis revealed that the exosomes were taken into the hiPSC-CM cytosol after 12 hr of incubation (Figure 5I). Thus, co-cultured hMSCs released various bioactive factors for cardiac cells, as well as exosomes transmissible to cardiac cells.

### hMSC-Derived Soluble Factors Impact hiPSC-CM Maturity

**Figure 6:**
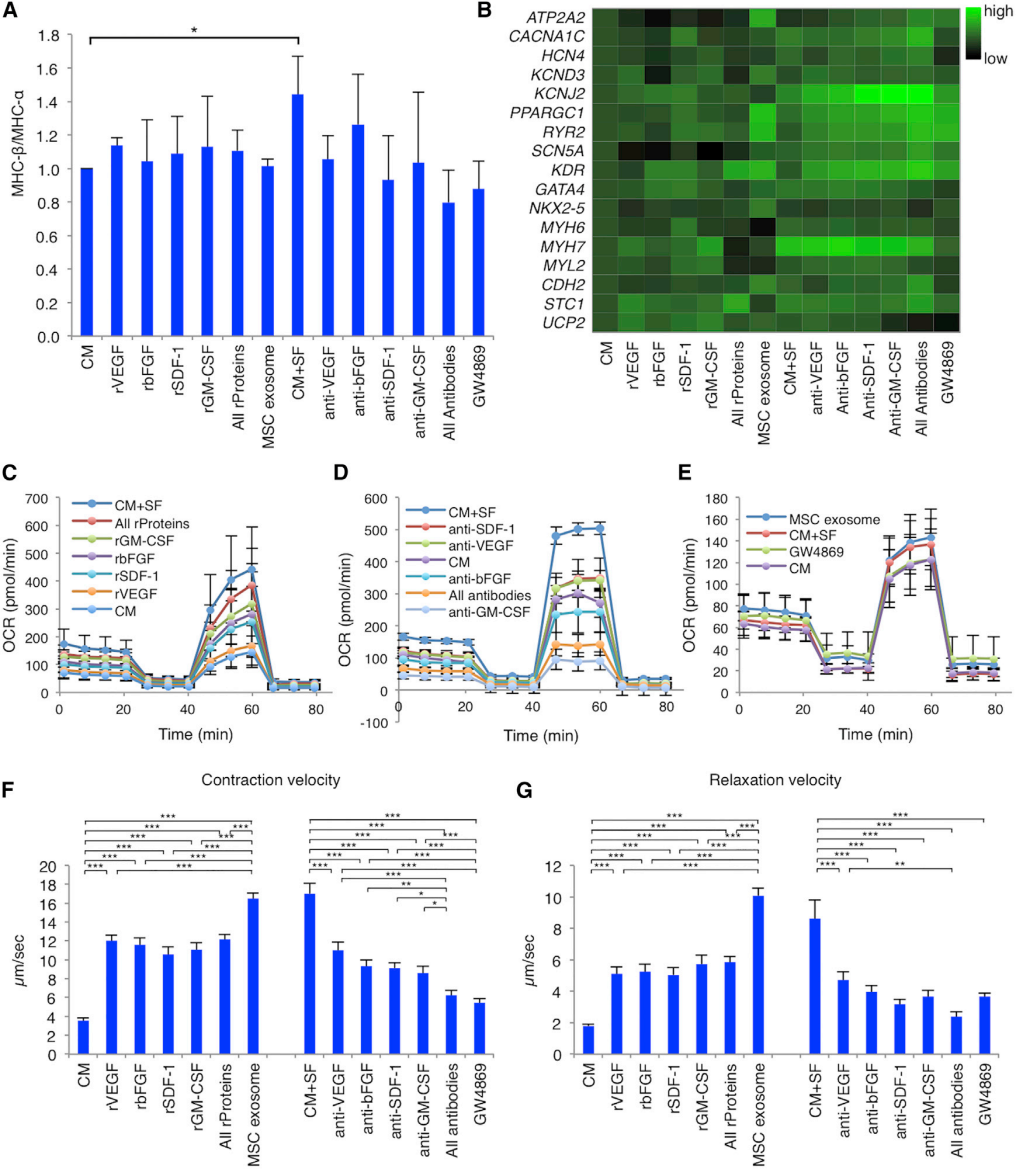
Impact of the Soluble Factors Derived from hMSCs on the Maturity of hiPSC-CMs Recombinant vascular endothelial growth factor (rVEGF), recombinant basic fibroblast growth factor (rbFGF), recombinant stromal cell-derived factor 1 (rSDF-1), recombinant granulocyte-macrophage colony-stimulating factor (rGM-CSF), all four recombinant proteins (all rProteins), or hMSC exosomes (MSC exosome) were added to culture media containing hiPSC-CMs (CM). Anti-VEGF neutralizing antibody (anti-VEGF), anti-bFGF neutralizing antibody (anti-bFGF), anti-SDF-1 neutralizing antibody (anti-SDF-1), anti-GM-CSF neutralizing antibody (anti-GM-CSF), all four neutralizing antibodies (all antibodies), or GW4879 were also added to culture media containing hiPSC-CMs with hMSC-derived soluble factors. (A) Ratio of myosin heavy chain (MHC)-β to MHC-α in all groups (n = 4 for each group). *p < 0.05, one-way ANOVA with post hoc Tukey’s HSD test. (B) Heatmap regarding expression of the cardiac genes in all groups, normalized against *GAPDH* expression (n = 9 for each group). (C) Representative mitochondrial respiration rates in the CM, rVEGF, rbFGF, rSDF-1, rGM-CSF, all rProteins, and CM+SF groups. (D) Representative mitochondrial respiration rates in the CM, anti-VEGF, anti-bFGF, anti-SDF-1, anti-GM-CSF, all antibodies, and CM+SF groups. (E) Representative mitochondrial respiration rates in the CM, MSC exosome, GW4879, and CM+SF groups. (F and G) Contraction velocity (F) or relaxation velocity (G) in all groups (n = 5 for each group). *p < 0.05; **p < 0.01; ***p < 0.001, one-way ANOVA with post hoc Tukey’s HSD test. For all experiments, results are shown as mean + SEM. OCR, oxygen consumption rate.

We investigated the impact of each hMSC-derived soluble factor on hiPSC-CM maturity using recombinant proteins and inhibitors. Although each recombinant cytokine showed a slightly increased ratio of a known cardiac maturation marker MHC-β to MHC-α (Figure 6A), the overall cytokine ratio did not increase as much as the hMSC-derived soluble factors. Conversely, the ratio decreased upon addition of each blocking antibody or 1 μM GW4869, an exosome secretion blocker. A heatmap generated to present the relative mRNA expression related to cardiac markers (Figure 6B) showed that exosomes impacted mRNA expression rather than cytokines. Regarding metabolic processes, each recombinant cytokine increased the OCR in every phase, with GM-CSF most effective, bFGF and SDF-1 moderately effective, and VEGF least effective. All recombinant cytokines yielded OCR similar to that in the CM+SF group (Figure 6C). Notably, each blocking antibody decreased the OCR in every phase, with GM-CSF most effective, then bFGF, VEGF, and SDF-1 least effective. In particular, bFGF and GM-CSF decreased the ratio more strongly than that in the CM group, likely because the hiPSC-CM-secreted cytokines were blocked (Figure 6D). The hMSC-released exosomes also increased the OCR, which was lowered upon 1 μM GW4869 addition (Figure 6E). Regarding motility, contraction and relaxation velocity were significantly increased by each recombinant cytokine and hMSC-released exosomes, but decreased by each blocking antibody and GW4869. Furthermore, hMSC exosomes influenced both velocities more strongly than any cytokine (Figures 6F and 6G). Thus, released factors, especially hMSC-derived exosomes, could exert beneficial effects on hiPSC-CM performance.

### MicroRNAs and Proteins in hMSC Exosomes

**Figure 7:**
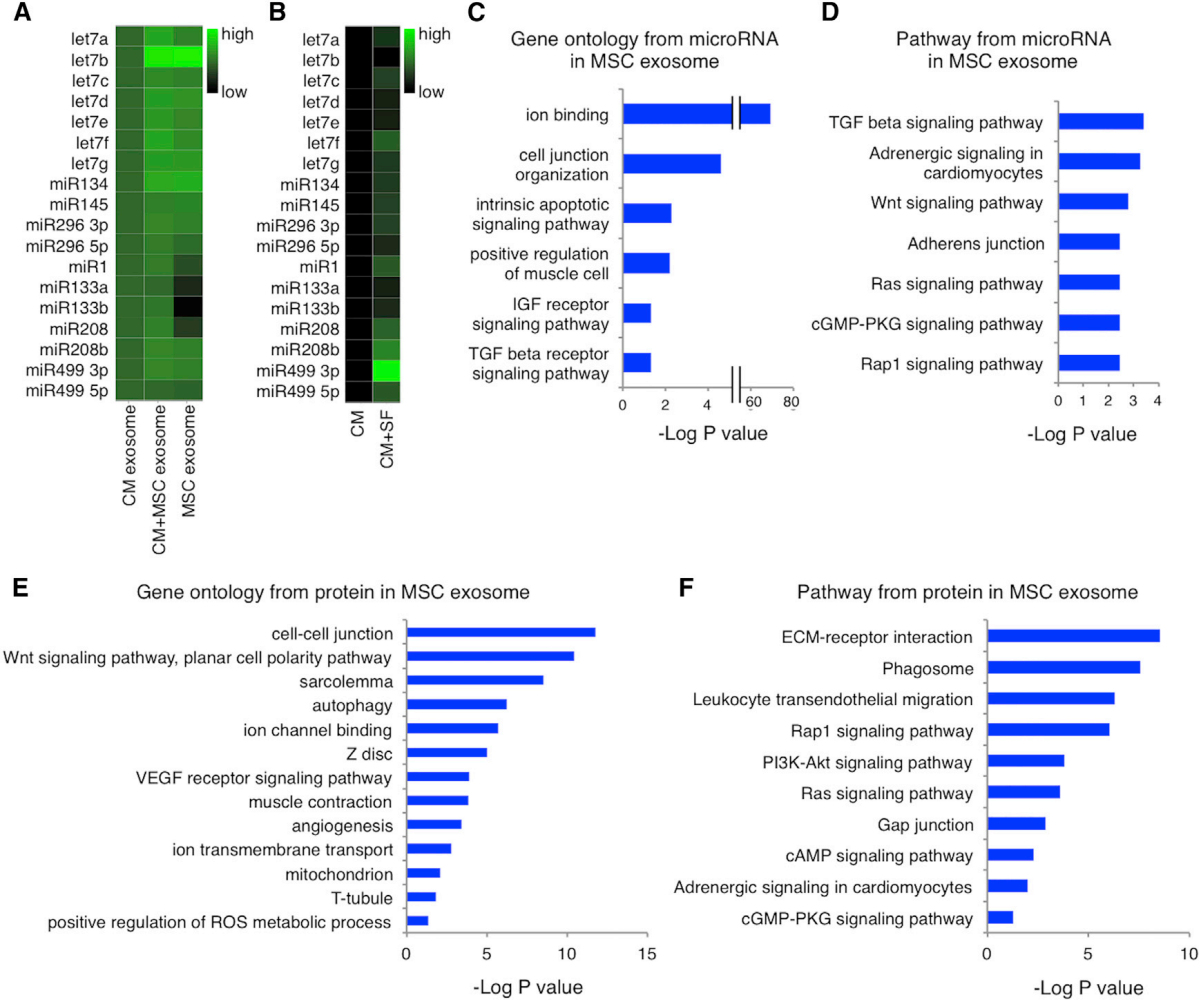
MicroRNAs and Proteins in hMSC-Derived Exosomes (A and B) Expression of microRNAs reported to promote the maturation of cardiomyocytes (let7 family, microRNA134, microRNA145, microRNA296) or to be specific to cardiomyocytes (microRNA1, microRNA133, microRNA208, microRNA499) in exosomes from culture media containing differentiated cardiomyocytes (CM), cardiomyocytes co-cultured with MSCs (CM+MSC), or MSC (A); and in CM or cardiomyocytes cultured with MSC-derived soluble factors (CM+SF) (B). (C and D) Gene ontology (C) and pathway analysis (D) of the microRNAs associated with the maturation of cardiomyocytes. The vertical axes show the gene ontology category and pathway category, respectively, and the horizontal axes show the extent of enrichment of each gene ontology category or pathway, respectively. (E and F) Gene ontology (E) and pathway analysis (F) of proteins in hMSC exosomes. The vertical axes show the gene ontology category and the pathway category, respectively, and the horizontal axes show the extent of enrichment of each gene ontology category or pathway, respectively.

We extracted microRNA from exosomes derived from the CM, CM+MSC, and MSC groups. A PCR-based microRNA microarray assay revealed the expression of microRNAs reported to promote cardiomyocyte maturation, including the let7 family, microRNA134, microRNA145, and microRNA296 (Table S3).^15^, ^26^, ^27^, ^28^, ^29^ These microRNAs were found at higher levels in hMSC-derived than in hiPSC-CM-derived exosomes (Figure 7A). Cardiomyocyte-specific microRNA levels, including microRNA1, microRNA133, microRNA208, and microRNA499,^30^, ^31^, ^32^, ^33^, ^34^, ^35^ were lower in hMSC-derived than in hiPSC-CM-derived exosomes. Such microRNA expression was higher in CM+MSC than in CM group exosomes. Next, we extracted microRNA from hiPSC-CMs with and without the addition of hMSC-derived soluble factors. CM+SF group cells showed higher expression of all of the above microRNAs than the CM group cells (Figure 7B).

Target prediction was performed using microT-CDS and DIANA mirPath v.3. Table S4 provides a detailed list of the identified target genes. Gene ontology enrichment analysis revealed that these genes were markedly enriched in the “cellular nitrogen compound metabolic process,” “ion binding,” “cell junction organization,” “extracellular matrix organization,” “cell adhesion,” “intrinsic apoptotic signaling pathway,” “positive regulation of muscle cell differentiation,” “histone acetylation,” “insulin-like growth factor receptor signaling pathway,” and “transforming growth factor beta (TGF-β) receptor signaling pathway” (Figure 7C). In addition, pathway analysis of the target genes of these microRNAs revealed that pathways associated with adrenergic signaling in cardiomyocytes and the TGF-β, FoxO, AMP-activated protein kinase (AMPK), Wnt, Ras, cyclic guanosine monophosphate-protein kinase G (cGMP-PKG), and Rap1 signaling pathways were upregulated (Figure 7D).

Proteomics analysis of the proteins extracted from the hMSC-derived exosomes identified 598 gene products (Table S5). Functional enrichment analysis of 463 of the gene products, which were classified as products found in extracellular exosomes, revealed marked enrichment in the Wnt signaling pathway, autophagy, muscle contraction, angiogenesis, responses to calcium ions, cell-cell junctions, and actin filaments (Figure 7E). Additionally, pathway analysis of these gene products revealed substantial enrichment of gap junctions, cyclic AMP (cAMP) signaling pathway, and adrenergic signaling in cardiomyocytes (Figure 7F).

### hiPSC-CM and hMSC Combination Enhanced Therapeutic Effects *In Vivo*

**Figure 8:**
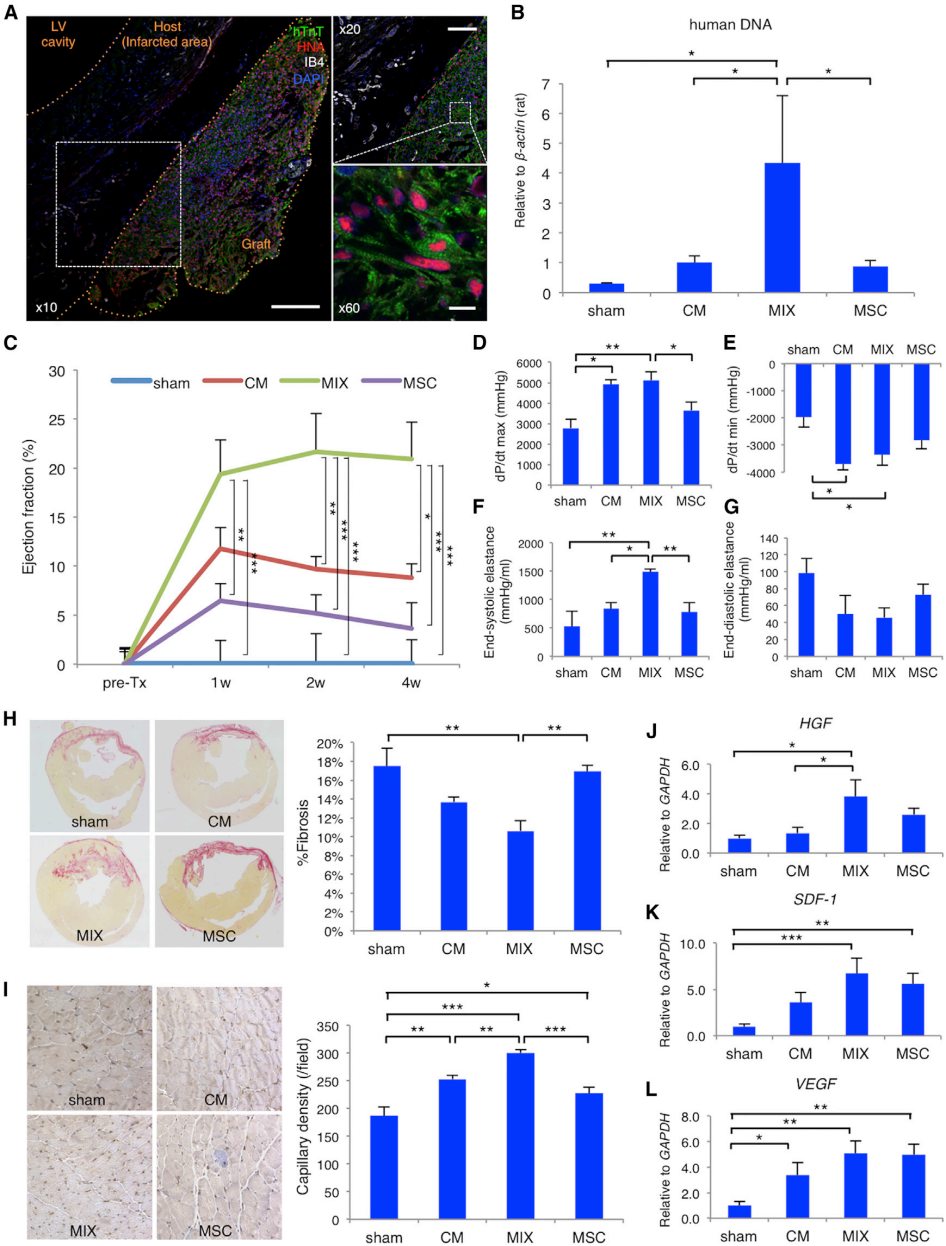
Combination of hiPSC-CMs and hMSCs Enhanced Therapeutic Effects *In Vivo* (A) Immunohistochemistry of human troponin T (hTnT; green), human nuclei (HNA; red), isolectin B4 (IB4; white), and nuclei (DAPI; blue) in a rat transplanted with a cell sheet containing both differentiated cardiomyocytes and MSCs (MIX) 4 weeks after cell sheet transplantation. Scale bars: 200 μm (left); 100 μm (right, top); 10 μm (right, bottom). (B) Expression of human DNA in the whole hearts of rat given no transplant (sham) or transplanted with a CM sheet, a MIX sheet, or an MSC sheet, normalized against rat *β-actin* expression. (C) Serial changes in the relative left ventricular ejection fraction in each group compared with sham rats, analyzed by transthoracic echocardiography (n = 10 for each group). pre-Tx, pre-transplantation; w, weeks after transplantation. (D–G) The dP/dt max (D), dP/dt min (E), end-systolic elastance (F), and end-diastolic elastance (G) in each group, analyzed by cardiac catheterization. (H) Left panels display representative images of myocardial fibrosis in each group, as assessed by Sirius Red staining. Right graph shows the percentage of fibrotic to myocardial tissue in each group (n = 8 for each group). (I) Left panels display representative images of immunohistochemistry of von Willebrand factor in each group. Right graph shows the capillary density per unit area in each group (n = 8 for each group). (J–L) Expression of the hepatocyte growth factor (*HGF*) (J), stromal cell-derived factor 1 (*SDF-1*) (K), or vascular endothelial growth factor (*VEGF*) (L) genes in each group, normalized against *GAPDH* expression (n = 8 for each group). For all experiments, one-way ANOVA with post hoc Tukey’s HSD test is used, and results are shown as mean + SEM. *p < 0.05; **p < 0.01; ***p < 0.001.

We performed cell sheets transplantation onto the hearts of athymic nude rats in a 2-week-old myocardial infarction model. Immunohistochemical analysis of cell sheets consisting of hiPSC-CM (CM sheet) or CM+MSC sheets (MIX sheet) revealed higher connexin 43 expression in the MIX sheet than in the CM sheet (Figure S3). Survival of the transplanted cells from the MIX sheets was observed 4 weeks after sheet transplantation through immunohistochemical images (Figure 8A), whereas transplanted cells from the other sheets were not detected at this time point. PCR showed that rats that had received MIX sheet transplantation had significantly higher human *GAPDH* levels, measured using total DNA extracted from whole heart samples (4.3 ± 2.3), than those receiving other sheets (sham: 0.3 ± 0.0, p = 0.0187; CM sheet: 1.0 ± 0.2, p = 0.0144; MSC sheet: 0.9 ± 0.2, p = 0.0190; Figure 8B). Figure 8C shows the relative left ventricular ejection fractions compared with sham rats through serial echocardiography. MIX sheet rats exhibited greater relative ejection fraction (19.4% ± 3.5%) than MSC sheet rats (6.5% ± 1.7%; p = 0.0033) and sham rats (0.0% ± 2.5%; p = 0.0007) at 1 week (ANOVA: p = 0.0004), 2 weeks (21.6% ± 3.9% [MIX sheet] versus 9.7% ± 1.3% [CM sheet], p = 0.0098, versus 5.2% ± 1.9% [MSC sheet], p = 0.0003, versus 0.0% ± 3.1% [sham], p < 0.0001; ANOVA: p < 0.0001), and 4 weeks after sheet transplantation (20.9% ± 3.8% [MIX sheet] versus 8.8% ± 1.5% [CM sheet], p = 0.0112; 3.6% ± 2.6% [MSC sheet], p = 0.0002, versus 0.0% ± 2.5% [sham], p < 0.0001; ANOVA: p < 0.0001).

Cardiac catheterization, performed to assess systolic and diastolic cardiac function,^36^, ^37^ showed significantly higher maximal rate of change in left ventricular pressure (dP/dt) in the MIX sheet (5,120 ± 400 mm Hg) than in the sham (2,782 ± 428 mm Hg; p = 0.0028) and MSC sheet groups (3,646 ± 392 mm Hg; p = 0.0150), and was slightly, but not significantly, higher than in the CM sheet group (4,906 ± 233 mm Hg; p = 0.7296; Figure 8D). The MIX sheet (−3,362 ± 384 mm Hg; p = 0.0351) and CM sheet groups (−3,701 ± 223 mm Hg; p = 0.0177) had significantly lower minimum dP/dt than the sham group (−1,974 ± 364 mm Hg; Figure 8E). The MIX sheet group had significantly higher end-systolic elastance (1,492 ± 45 mm Hg/mL) than other groups (sham: 526 ± 269 mm Hg/mL, p = 0.0053; CM: 836 ± 106 mm Hg/mL, p = 0.0255; MSC: 780 ± 170 mm Hg/mL, p = 0.0096; Figure 8F). The MIX sheet group had slightly lower end-diastolic elastance (46 ± 12 mm Hg/mL) than the other groups (sham: 99 ± 17 mm Hg/mL, p = 0.0802; CM sheet: 50 ± 22 mm Hg/mL, p = 0.8726; MSC sheet: 73 ± 12 mm Hg/mL, p = 0.3909; Figure 8G).

Additionally, the MIX sheet group fibrotic area in recipient cardiac tissue (11% ± 1%), which was positively associated with the region of myocardial infarction, was smaller than those of the sham (17% ± 2%; p = 0.0068) and MSC sheet groups (17% ± 1%; p = 0.0028; Figure 8H). The MIX sheet group capillary density (301 ± 6/field) was higher than those of the sham (187 ± 15/field; p < 0.0001), CM sheet (253 ± 7/field; p = 0.0028), and MSC sheet groups (228 ± 12/field; p < 0.0001; Figure 8I).

qRT-PCR analysis revealed that the MIX sheet group displayed higher relative hepatocyte growth factor (HGF; 3.8 ± 1.1) expression than the sham group (1.0 ± 0.2; p = 0.0107) and the CM sheet group (1.4 ± 0.4, p = 0.0235; Figure 8J). Although the expressions of *SDF-1* and *VEGF* were not different between the CM sheet, MSC sheet, and MIX sheet groups, the MIX sheet group showed a relatively higher SDF-1 (6.7 ± 1.6) and VEGF (5.1 ± 1.0) expression than the sham group (1.0 ± 0.3; p = 0.0007; Figure 8K; and 1.0 ± 0.3, p = 0.0011; Figure 8L, respectively). Thus, the MIX sheet could not only maintain cardiac properties *in vivo*, but also improved the functionality of transplanted hearts.

## Discussion

The major finding of this study was that hMSC co-culture enhanced hiPSC-CM functionality in structural, motility, electrophysiological, and metabolic aspects. This conclusion is supported by the following experiments. hMSC co-culture allowed hiPSC-CMs to: (1) increase cardiomyocyte purity; (2) increase the MHC-β-to-MHC-α ratio; (3) develop a rod-shaped morphology; (4) have fully formed mitochondria and aligned myofibrils with A-, H-, and I-bands; (5) produce more energy under normal and stressed conditions; and (6) reduce ROS production under oxidative stress. Thus, hMSC co-culture promoted iPSC-CM differentiation and enhanced myofibril maturation. Furthermore, hMSC co-culture allowed hiPSC-CMs to: (7) have clear gap junctions, (8) increase connexin 43 and N-cadherin expression, (9) contract and relax quickly, and (10) have an electric pacing of >2 Hz. Thus, hMSC co-culture promoted the iPSC-CM structural framework and enhanced cell-cell interactions. Finally, cell sheets consisting of a mixture of hiPSC-CMs and hMSCs showed longer survival and enhanced therapeutic effects after transplantation into athymic nude rats in a 2-week-old myocardial infarction model, compared with cell sheets consisting of hiPSC-CMs without hMSCs. Collectively, hMSC co-culture promoted iPSC-CM maturation and survival.

Following hiPSC establishment in 2007, they were expected to become a cell source for therapeutic applications such as regenerative medicine, disease modeling, drug screening, and toxicity testing.^38^, ^39^, ^40^, ^41^ The efficiency of cardiomyocyte differentiation from hiPSCs has been greatly improved in recent years,^38^ with many reports describing over 60% cardiomyocytes in differentiated cultures. These cardiomyocytes exhibit sarcomeres, calcium transients, and spontaneous beating, but display a low degree of maturation based on the studied parameters.^42^, ^43^ A general consensus has emerged that maturation protocols must be developed to maximize hiPSC-CM therapeutic applications. In the present study, hMSC co-culture for 3 days, using a relatively simple method, affected various parameters representing the degree of cardiomyocyte maturity in adult hearts.

Although hMSC co-culture enhanced iPSC-CM maturation, the specific underlying mechanisms remain unclear. The enhancement of hiPSC-CM maturation caused by hMSC co-culture was observed even in a transwell assay, indicating that hMSC-secreted soluble factors comprised the primary cause of the mechanism. However, it was unclear which factor promoted differentiation and maturation. The present study revealed that hMSCs release cytokines such as VEGF, bFGF, SDF-1, and GM-CSF that are capable of modulating hiPSC-CM functionality. Notably, cytokines that have not been previously used in differentiation protocols for hiPSC-CMs, such as GM-CSF and SDF-1, had a greater influence on hiPSC-CM respiratory capacity than those used in current differentiation methods, such as VEGF and bFGF.

Furthermore, the present study demonstrated that hiPSC-CM functionality was also affected by the exosomes released from hMSCs. Exosomes are small vesicles, 30–150 nm in diameter, containing a wide range of functional proteins and microRNAs. Recent studies have revealed that exosomes play an important role in cell-to-cell communication as intercellular messengers.^44^, ^45^ We demonstrated that exosomes released from hMSCs included microRNAs previously shown to promote cardiomyocyte maturation.^15^, ^26^, ^27^, ^28^, ^29^ Additionally, gene ontology and pathway analysis revealed that the microRNAs and the proteins in the hMSC-released exosomes impacted hiPSC-CM functionality and maturation. These results suggested that hiPSC-CM maturation could not be induced by a single factor, but rather by multiple factors that modulate a wide variety of genes or pathways.

hMSC co-culture promoted hiPSC-CM survival, although the mechanisms controlling this remain unclear. The transplanted cells are insufficiently supported by a vascular network of native myocardial cells, resulting in cellular stress.^21^ The angiogenic potential of hMSCs might contribute to the longer hiPSC-CM survival in ischemic areas. This study revealed that hMSC co-culture allowed hiPSC-CMs to produce more energy by aerobic respiration under stressed conditions and to suppress ROS production induced by oxidative stress. In general, ROS play important roles in the regulation of cell survival; a sharp increase in ROS can induce cell death.^46^, ^47^ The suppression of ROS production by hMSCs could reduce cell death around the transplanted site *in vivo*. Moreover, hMSC co-culture allowed hiPSC-CMs to have clear gap junctions and to increase connexin 43 and N-cadherin expression. Gap junction channels allow the intercellular passage of small molecules and regulate essential processes,^48^ and intercellular communication through gap junctions plays vital roles in cell differentiation and survival.^49^ In addition, N-cadherin is essential for cell-cell contact in cardiomyocytes *in vivo* and *in vitro*,^50^ with N-cadherin adhesion playing important roles in cardiomyocyte differentiation and survival.^51^ Moreover, hMSCs may promote the formation of new intercalated disc-like structures between implanted and host cells, resulting in the synchronous beating of implanted hiPSC-CMs and host cells.

There were a few limitations to this study. The experiments in this study were performed using only a single cell line. Another cell line may yield different results; nevertheless, new concepts concerning maturation or transplantation methods were suggested through this research. In addition, too many factors may be associated with hiPSC-CM maturation to clarify each factor precisely, although hMSC-derived soluble factors caused hiPSC-CM maturation. Some factors might simultaneously contribute to the hiPSC-CM maturation in some aspects, but not in other aspects. The differentiation or maturation methods of hiPSC-CMs require further comprehensive analyses. Overall, however, the generated matured hiPSC-CMs may be useful for regenerative medicine, as well as disease modeling, drug screening, and toxicity testing. On the other hand, the regulation and quality control in both cells might be more complex than those in only hiPSC-CMs, when using this maturation method in a clinical setting.

In conclusion, this study provides a proof-of-concept and useful baseline data for future research aimed at elucidating the mechanisms underlying these morphological and functional changes. Co-culture with hMSCs was clearly shown to modulate the maturity and functionality of hiPSC-CMs *in vitro* and to enhance the survival and therapeutic potential of hiPSC-CMs for heart failure following myocardial infarction *in vivo*.

## Materials and Methods

### hMSC Culture

A population of hMSCs from human bone marrow was purchased from Lonza (Basel, Switzerland) and maintained in MSC basal media (Lonza). hMSCs at P4 to P6 were used for all experiments in this study.

### Cardiac Differentiation of hiPSCs

Cardiomyogenic differentiation from hiPSC line 253G1 was induced using a previously reported bioreactor system (Figure 1A).^39^, ^52^ Details are given in the Supplemental Materials and Methods (see Cardiac Differentiation of Human Induced Pluripotent Stem Cells section).

### Co-culture with hMSCs

hiPSC-CMs were cultured on new dishes with the same number of hMSCs (CM+MSC) or without hMSCs (CM) for 3 days after differentiation in DMEM high glucose (Thermo Fisher Scientific, Waltham, MA, USA). To assess the effects of hMSC-secreted soluble factors, we also co-cultured hiPSC-CMs and hMSCs without direct cell-cell contact using Transwell inserts (3.0-μm pore polycarbonate membrane; Corning, Armonk, NY, USA) for 3 days; hMSCs were removed before assay performance (CM+SF) (Figure S1). Because the wells in 96-well plates were too small to culture equivalent hMSC numbers, 40,000 hiPSC-CMs were co-cultured therein with 20,000 (CM+SF 50%) or 10,000 hMSCs (CM+SF 25%). For all other experiments, hiPSC-CMs were co-cultured with equivalent hMSC numbers per plate.

### Cell Sheet Preparation and Transplantation

In temperature-responsive culture dishes (UpCell; CellSeed, Tokyo, Japan),^25^ we prepared three types of cell sheets as follows: (1) 1 × 10^6^ hiPSC-CMs (CM sheet), (2) 1 × 10^6^ hiPSC-CMs with 1 × 10^6^ hMSCs (MIX sheet), and (3) 1 × 10^6^ hMSCs (MSC sheet). Each cell sheet, or no sheet (sham), was transplanted and attached by several sutures onto the anterior wall of the left ventricle of athymic nude rats (F344/NJcl-rnu/rnu, 7 weeks old, male, 120–130 g; CLEA Japan, Osaka, Japan) 2 weeks after permanent ligation of the proximal site of the left anterior descending artery. Animal care procedures were consistent with the *Guide for the Care and Use of Laboratory Animals* (NIH). Experimental protocols were approved by the Ethics Review Committee for Animal Experimentation of Osaka University Graduate School of Medicine (reference no. 25-025-045).

### Isolation of Exosomes from Cell Culture Media

Conditioned media were collected from cells grown in serum-free media for 48 hr. Dead cells and contaminating cell debris were removed by centrifugation at 300 × *g* for 10 min and then at 2,000 × *g* for 10 min at 4°C. Media were subjected to ultracentrifugation (SW32Ti, Ultra-Clear tube; Beckman Coulter, Brea, CA, USA) at 175,000 × *g* for 120 min at 4°C; ultracentrifugation of the resulting pellet was repeated following washing with PBS to produce a pellet containing extracellular vesicles including exosomes.

## Author Contributions

S.Y. designed research studies, conducted experiments, acquired data, analyzed data, and wrote the manuscript. S.M. designed research studies, obtained funding, and revised the manuscript. S.F., T.K., and N.K. designed research studies and searched literature. F.O. conducted experiments, acquired data, and analyzed data. T.T. designed research studies, searched literature, and revised the manuscript. K.T. designed research studies and searched literature. Y.S. designed research studies, obtained funding, and approved the article.

## Supplemental Information

Document S1. Supplemental Materials and Methods, Figures S1–S3, and Tables S1 and S2

Table S3. Microarray for MicroRNAs in hiPSC-CMs or Exosomes in Culture Media

Table S4. Target Prediction with Gene Expression from Identified MicroRNAs in Exosomes

Table S5. Proteomics of MSC Exosomes

Document S2. Article plus Supplemental Information
