# Supplementary material for: Maturation of Human Induced Pluripotent Stem Cell-Derived Cardiomyocytes by Soluble Factors from Human Mesenchymal Stem Cells
Source: Mol Ther. 2018 Aug 16;26(11):2681–95. doi: 10.1016/j.ymthe.2018.08.012 (PMC6224789; doi:10.1016/j.ymthe.2018.08.012)
Supplement: Document S1. Supplemental Materials and Methods, Figures S1–S3, and Tables S1 and S2 [file mmc1.pdf]

YMTHE, Volume *26*

Supplemental Information

Maturation of Human Induced Pluripotent Stem Cell-Derived Cardiomyocytes by Soluble Factors from Human Mesenchymal Stem Cells

Shohei Yoshida, Shigeru Miyagawa, Satsuki Fukushima, Takuji Kawamura, Noriyuki Kashiyama, Fumiya Ohashi, Toshihiko Toyofuku, Koichi Toda, and Yoshiki Sawa

SUPPLEMENTAL INFORMATION

Figure S1. Co-culture of hiPSC-CMs and hMSCs.
Figure S2. Concentration of Cytokines in Culture Media.
Figure S3. Immunohistochemistry of Cell Sheets.
Table S1. Lists of Primers Used in This Study.
Table S2. Lists of Primary and Secondary Antibodies Used in This Study.
Table S3. Microarray for MicroRNAs in hiPSC-CMs or Exosomes in Culture Media.
Table S4: Target Prediction with Gene Expression from Identified MicroRNAs in Exosomes.
Table S5. Proteomics of MSC Exosomes.
Movie S1. Representative Video of hiPSC-CMs in the CM Group without Color.
Movie S2. Representative Video of hiPSC-CMs in the CM Group with Color.
Movie S3. Representative Video of hiPSC-CMs in the CM+SF Group without Color.
Movie S4. Representative Video of hiPSC-CMs in the CM+SF Group with Color.
Supplemental Materials and Methods
References

SUPPLEMENTAL FIGURE

Figure S1. Co-culture of hiPSC-CMs and hMSCs.

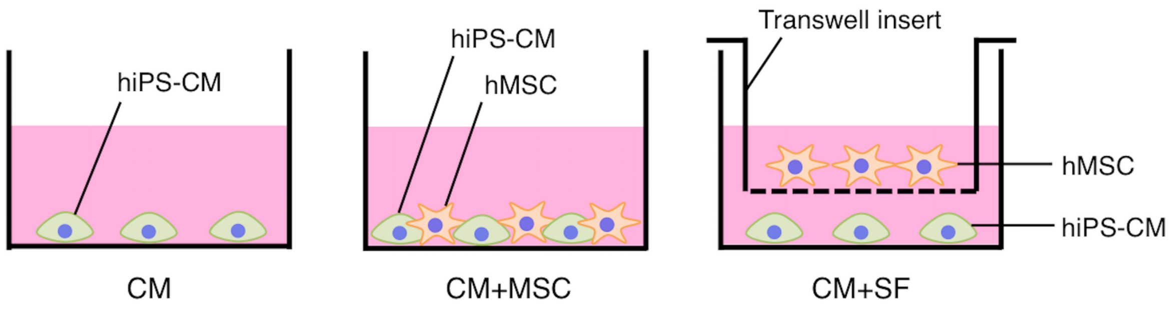

hiPSC-CMs were cultured on new dishes with the same number of hMSCs (CM+MSC) or without hMSCs (CM) for three days after differentiation in Dulbecco's modified Eagle's medium high glucose. hiPSC-CMs and hMSCs were also co-cultured without direct cell-cell contact using Transwell inserts (3.0-µm pore polycarbonate membrane) for three days; hMSCs were removed before assay performance (CM+SF).

Figure S2. Concentration of Cytokines in Culture Media.

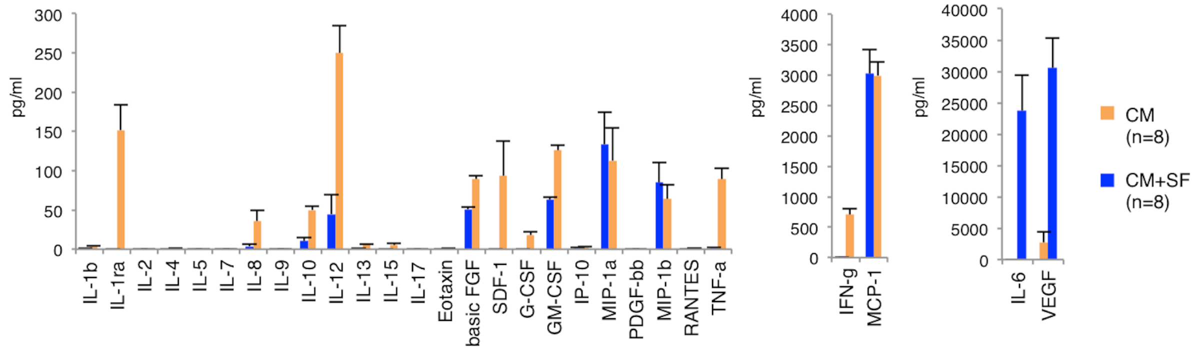

The concentration of each cytokine in culture media containing differentiated cardiomyocytes (CM) and cardiomyocytes cultured with mesenchymal stem cells derived soluble factors (CM+SF; n = 8, for each group), analyzed by an enzyme-linked immunosorbent assay (ELISA) kit and the Bio-plex suspension array system.

Figure S3. Immunohistochemistry of Cell Sheets.

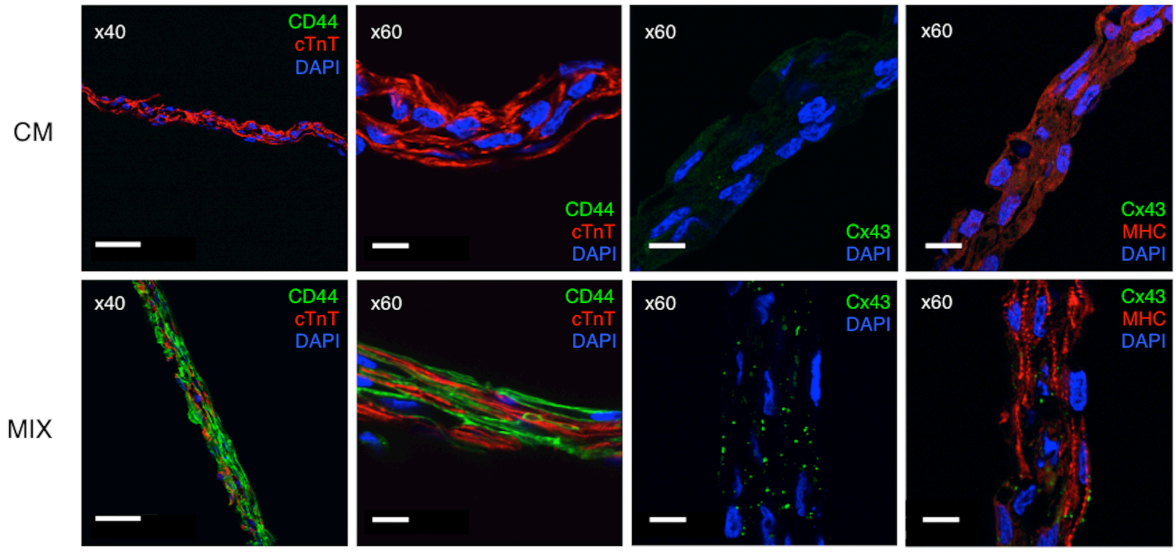

Cell sheets containing differentiated cardiomyocytes (CM, upper panels) mainly consisted of cardiac troponin T (cTnT)-positive cells, whereas cell sheets containing a mixture of CM and mesenchymal stem cells (MIX; lower panels) consisted of cTnT-positive cells and CD44-positive cells (left). The expression of connexin 43 (Cx43) in the MIX sheets was higher than in the CM sheets (right). Scale bar: 50 µm (left) and 10 µm (middles, right).

SUPPLEMENTAL TABLE

Table S1. Lists of Primers Used in This Study.

Gene Name	Primers	
CDH2	TaqMan	Hs00983056_m1
RYR2	TaqMan	Hs00181461_m1
ATP2A2	TaqMan	Hs00544877_m1
CACNA1C	TaqMan	Hs00167681_m1
KCND3	TaqMan	Hs00542597_m1
KCNJ2	TaqMan	Hs01876357_s1
SCN5A	TaqMan	Hs00165693_m1
HCN4	TaqMan	Hs00975492_m1
PPARGC1A	TaqMan	Hs01016719_m1
STC1	TaqMan	Hs00174970_m1
KDR	TaqMan	Hs00911700_m1
GJA1	TaqMan	Hs00748445_s1
β-actin (rat)	Europhins	1247000
GATA4	SYBR	F: 5'-AGGCCTCTTGCAATGCGGA-3'<br>R: 5'-CTGGTGGTGGCGTTGCTGG-3'
NKX2.5	SYBR	F: 5'-ACCTCAACAGCTCCCTGACTC-3'<br>R: 5'-ATAATCGCCGCCACAAACTCTCC-3'
MYH6	SYBR	F: 5'-TCAGCTGGAGGCCAAAAGTAAAGGA-3'<br>R: 5'-TTCTTGAGCTCTGAGCACTCGTCT-3'
MYH7	SYBR	F: 5'-TCGTGCCTGATGACAAACAGGAGT-3'<br>R: 5'-ATACTCGGTCTCGGCAGTGACTTT-3'
MYL2	SYBR	F: 5'-TGTCCCTACCTTGTCTGTTAGCCA-3'<br>R: 5'-ATTGGAACATGGCCTCTGGATGGA-3'
ALU	SYBR	F: 5'-GTCAGGAGATCGAGACCATCCC-3'<br>R: 5'-TCCTGCCTCAGCCTCCCAAG-3'
NADH	SYBR	F: 5'-ATACCCATGGCCAACCTCCT-3'<br>R: 5'-CGTCAGCTCGTGTTGTGAAA-3'
COX3	SYBR	F: 5'-ATGACCCACCAATCACATGC-3'<br>R: 5'-ATCACATGGCTAGGCCGGAG-3'

Table S2. Lists of Primary and Secondary Antibodies Used in This Study.

Antibody	Source
**For Immunocytochemistry and Immunohistochemistry**	
cTnT	Santa Cruz Biotechnology, sc-20025
MHC	Invitrogen, MA183347
Hoechst 33342	Dojindo, H342
Cx43	Sigma, C6219
N-cadherin	Abcam, ab12221
RNA cargo	Invitrogen, S32703
BODIPY™ TR Ceramide	Invitrogen, D7540
Phalloidin	Invitrogen, A34055
hTnT	Abcam, ab91605
HNA	Chemicon, MAB1281
IB4	Invitrogen, l32450
DAPI	Vector, H-1200
vWF	DAKO, A0082
CD44	Abcam, ab15107
Mouse IgG (H+L)	Life Technologies, A11001
Mouse IgG (H+L)	Life Technologies, A21422
Rabbit IgG (H+L)	Life Technologies, A11008
Rabbit IgG (H+L)	Life Technologies, A21428
**For Western Blotting**	
CD63	Invitrogen, 10628D
MHC-α	Abcam, ab185967
MHC-β	Abcam, ab172967
GAPDH	Abcam, ab8245
Beta Actin	Abcam, ab8224
Mouse IgG	GE, NA934-1ML
Rabbit IgG	GE, NA931-1ML
**For Flow Cytometry**	
Cardiac TnT	Santa Cruz Biotechnology, sc-20025
Isotype Control	Santa Cruz Biotechnology, sc-2025
Mouse IgG	Life Technologies, A11011
**For Immunoelectron Microscopy**	
CD63	Invitrogen, 10628D
Mouse IgG	Abcam, ab39619

cTnT: cardiac troponin T, hTnT: human troponin T, MHC: myosin heavy chain, Cx43: Connexin-43, HNA: human nuclear antigen, IB4: isolectin B4, DAPI: 4',6-diamidino-2-phenylindole, vWF: von Willebrand factor

Table S3. Microarray Analysis of MicroRNAs in hiPSC-CMs or Exosomes in Culture Media.

CM: differentiated cardiomyocytes derived from human induced pluripotent stem cells, MSC: human mesenchymal stem cells, SF: soluble factors secreted from human mesenchymal stem cells.

Table S4. Target Prediction with Gene Expression from Identified MicroRNAs in Exosomes.

GO: Gene Ontology, KEGG: Kyoto Encyclopedia of Genes and Genomes.

Table S5. Proteomics of MSC Exosomes.

GO: Gene Ontology, KEGG: Kyoto Encyclopedia of Genes and Genomes

SUPPLEMENTAL MOVIE

Movie S1. Representative Video of hiPSC-CMs in the CM Group without Color.

Movie S2. Representative Video of hiPSC-CMs in the CM Group with Color.
Red and blue represent a high and low velocity, respectively.

Movie S3. Representative Video of hiPSC-CMs in the CM+SF Group without Color.

Movie S4. Representative Video of hiPSC-CMs in the CM+SF Group with Color.
Red and blue represent a high and low velocity, respectively.

SUPPLEMENTAL MATERIALS AND METHODS

Cardiac Differentiation of Human Induced Pluripotent Stem Cells (hiPSCs)

hiPSC cell line 253G1, which was established in CiRA using the four Yamanaka factors, was used in this study.[1] Undifferentiated hiPSCs were expanded and maintained on SNL feeder cell layers in primate embryonic stem (ES) cell media (ReproCell, Tokyo, Japan) supplemented with 4 ng/ml basic fibroblast growth factor (bFGF) (Wako, Osaka, Japan). Cardiomyogenic differentiation was induced using a bioreactor system as previously reported.[2] Undifferentiated hiPSCs were detached and dissociated into single cells by 5 min incubation with Accumax (Innovative Cell Technologies, San Diego, CA, USA). The cells were then allowed to form embryoid bodies through suspension in modified Tenneille Serum Replacer 1 media (STEMCELL Technologies, Vancouver, Canada) supplemented with 10 µM Y-27632 (Wako) in bioreactors for 2 days (Figure 1a). On day 2, the media were changed to StemPro 34 media (Thermo Fisher Scientific, Waltham, MA, USA) supplemented with 0.5 ng/ml bone morphogenetic protein 4 (BMP4) (R&D Systems, Minneapolis, MN, USA); on day 3, media including human recombinant activin A (R&D Systems), BMP4, and basic fibroblast growth factor (bFGF) were added to the bioreactors. The final concentrations were as follows: activin A, 3 ng/ml; BMP4, 10 ng/ml; and bFGF, 5 ng/ml. On day 6, media including IWP-3, a Wnt inhibitor (Stemgent, Cambridge, MA, USA) were added at a final concentration of 4 µM IWP-3. On day 10, the media were changed to Stem Pro 34 supplemented with 5 ng/ml vascular endothelial growth factor (VEGF; R&D Systems) and 10 ng/ml bFGF. On day 16, embryoid bodies were dissociated using Accumax (Innovative Cell Technologies) for 10 min, and the number of dissociated cells was counted by Countes (Thermo Fisher Scientific).

hiPSC-derived cardiomyocytes (hiPSC-CMs) were cultured on new dishes with the same number of hMSCs (CM+MSC) or without hMSCs (CM) for three days after differentiation in Dulbecco's modified Eagle's medium (DMEM) high glucose (Thermo Fisher Scientific). To assess the effects of hMSC-secreted soluble factors, hiPSC-CMs and hMSCs were also co-cultured without direct cell-cell contact using Transwell inserts (3.0-µm pore polycarbonate membrane, Corning Inc., Armonk, NY, USA) for 3 days; hMSCs were removed before assay performance (CM+SF). As the wells in 96-well plates were too small to culture equivalent hMSC numbers, 40,000 hiPSC-CMs were co-cultured therein with 20,000 (CM+SF 50%) or 10,000 hMSCs (CM+SF 25%). For all other experiments, hiPSC-CMs were co-cultured with equivalent hMSC numbers per plate.

Flow Cytometry

Cardiomyocytes derived from human induced pluripotent stem cells (hiPSC-CMs) were dissociated with 0.25% trypsin-EDTA (Thermo Fisher Scientific), fixed with CytoFix fixation buffer (Becton Dickinson, Franklin Lakes, NJ, USA) for 20 min, permeabilized with Perm/Wash buffer (Becton Dickinson) at room temperature for 10 min, and then incubated with human anti-troponin T (TnT) antibody (Santa Cruz Biotechnology, Dallas, TX, USA) for 30 min. The labeled cells were washed with Perm/Wash buffer prior to incubation with the secondary antibody at room temperature for 30 min, and then assayed using a FACS Canto II (Becton Dickinson). The hiPSC-CMs in the CM and CM+SF groups were used in this experiment and the hiPSC-CMs in the CM+MSC group were excluded, because co-existence with hMSCs made an accurate measurement in this experiment impossible.

Quantitative Real-Time PCR (RT-qPCR)

Total RNA from hiPSC-CMs *in vitro* or cardiac tissue after cell sheet transplantation *in vivo* was isolated using the PureLink RNA Mini Kit (Thermo Fisher Scientific) or RNeasy Fibrous Tissue Mini Kit (Qiagen, Hilden, Germany), respectively. RNA was reverse transcribed to cDNA using the SuperScript III reverse transcription kit (Thermo Fisher Scientific). RT-qPCR was performed using the Viia7 Real-Time PCR system (Thermo Fisher Scientific) in triplicate for each sample with TaqMan (Thermo Fisher Scientific) or SYBR green (Thermo Fisher Scientific) probes. The samples were normalized against the housekeeping gene glyceraldehyde-3-phosphate dehydrogenase (*GAPDH*). The hiPSC-CMs in the CM and CM+SF groups were used in this experiment and the hiPSC-CMs in the CM+MSC group were excluded, because co-existence with hMSCs made an accurate measurement in this experiment impossible.

For quantification of mitochondrial damage, RT-qPCR was performed on mitochondrial genes including human cytochrome C oxidase subunit III (*COX3*) and human *NADH* dehydrogenase using culture media in the CM, CM+MSC, and CM+SF groups.

For quantification of engraftment, RT-qPCR for genomic DNA was performed 4 weeks after cell sheet transplantation using the Viia7 Real-Time PCR system in triplicate for each sample.[3] Genomic DNA was extracted from the whole LV using the PureLink Genomic DNA Mini Kit (Thermo Fisher Scientific). The samples were normalized to the rat housekeeping gene *β-actin*.

The primers used for all PCR analyses can be found in Table SI.

Western Blotting

Total protein was acquired from cardiomyocytes with or without co-culture and subjected to sodium dodecyl sulfate-polyacrylamide gel electrophoresis. After blocking with blocking one (Nakalai Tesque, Osaka, Japan), the membranes were incubated with anti-myosin heavy chain alpha (MHC-α) rabbit monoclonal antibody, anti-MHC-β rabbit monoclonal antibody, or anti-GAPDH mouse monoclonal antibody (Abcam, Cambridge, UK) overnight with shaking at 4 °C. After incubation with anti-rabbit or anti-mouse horseradish peroxidase-coupled secondary antibody (Santa Cruz Biotechnology), bands were visualized using Amersham ECL Prime Western Blotting Detection Reagent (GE Healthcare, Little Chalfont, UK) and quantified using the ChemiDoc MP Imaging System (Bio-Rad Laboratories, Hercules, CA, USA). The hiPSC-CMs in the CM and CM+SF groups were used in this experiment and the hiPSC-CMs in the CM+MSC group were excluded, because co-existence with hMSCs made an accurate measurement in this experiment impossible.

Exosomes isolated from the supernatant of hMSCs were examined by western blotting using anti-CD63 antibody (Thermo Fisher Scientific) with the same procedures explained above.

Immunocytochemistry and Immunohistochemistry Analysis

Dissociated single cells or harvested hearts were fixed with 4% paraformaldehyde and labeled with primary antibodies, followed by incubation with fluorescence-conjugated secondary antibodies, counterstaining with 4', 6-Diamidino-2-phenylindole (DAPI) (Vector Laboratories, Burlingame, CA, USA) or Hoechst33258 (Dojindo, Kumamoto, Japan), and finally analysis by confocal microscopy (FV1200 or SD-OSR, Olympus, Tokyo, Japan). The labeled cells were captured based on their fluorescence intensity. A list of the antibodies used can be found in Supplemental Table SI. The hiPSC-CMs in the CM, CM+MSC, and CM+SF groups were used in this experiment.

Electron Microscopy

hiPSC-CMs were fixed overnight in 1/2 strength Karnovsky's (2% paraformaldehyde/2.5% glutaraldehyde buffered with 0.2 M cacodylate) and post-fixed in 2% OsO_4 buffer. After dehydration, cells were embedded in Quetol 812 (Nissin EM, Tokyo, Japan), sectioned into 70-nm slices, and stained with uranyl acetate for 2 h and lead citrate for 5 min. The samples were imaged using an electron microscope (Hitachi H-7500; Hitachi, Tokyo, Japan) set to 80 kV. The hiPSC-CMs in the CM, CM+MSC, and CM+SF groups were used in this experiment.

Cell Motility Analysis

Beating cells were monitored at a rate of 150 Hz for 6 s at 37 °C with a high-speed camera-based motion analysis system (SI8000 View; Sony, Tokyo, Japan). The beating area, contraction velocity, relaxation velocity, and acceleration were measured using SI8000C Analyzer (Sony). The hiPSC-CMs in the CM and CM+SF groups were used in this experiment and the hiPSC-CMs in the CM+MSC group were excluded, because co-existence with hMSCs made an accurate measurement in this experiment impossible.

Ca^{2+} Transient Measurement

Ca^{2+} transient measurement was performed using 96-well plates. After hiPSC-CMs were washed with phosphate buffered saline (PBS), cells were loaded with 5 μM Fluo-8 regents (AAT Bioquest, Sunnyvale, CA, USA) in serum-free minimal essential media (MEM) at 37 °C for 30 min. Intracellular fluorescence was recorded with or without pacing at 0.5–3 Hz at 37 °C using an FDSS/μCELL system (Hamamatsu Photonics, Hamamatsu, Japan). Data were analyzed using FDSS software U8524-12 (Hamamatsu Photonics) to obtain beating rate, peak ratio, rising slope, and peak width duration. Fluorescence intensity was described using relative fluorescence units (rfu) in this study. The hiPSC-CMs in the CM, CM+SF 25%, and CM+SF 50% groups were used in this experiment because 96-well plates should be used for this experiment. The hiPSC-CMs in the CM+MSC group were excluded, because co-existence with hMSCs made an accurate measurement in this experiment impossible.

Mitochondrial Function Assay

The Seahorse XF96 extracellular flux analyzer was used to assess mitochondrial function. Plates were pre-treated with bovine serum albumin; at 16 days after differentiation, the cells were seeded onto the plates at a density of 40,000 cells per XF96 well. The cells were cultured at 37 °C for 3 days in the Seahorse plates before analysis. Culture medium was exchanged for a basal medium (XF assay medium supplemented with 25 mM glucose and 1 mM sodium pyruvate) 1 h before the assay and for the duration of the measurement of oxygen consumption rate (OCR) and extracellular acidification rate (ECAR). Substrates and selective inhibitors were injected during the measurements to achieve final concentrations of 25 mM glucose, 2.5 μM oligomycin, 1 μM carbonyl cyanide-p-trifluoromethoxyphenylhydrazone (FCCP), 2.5 μM rotenone, and 2.5 μM antimycin A. The basal respiration rate was defined as the average values of OCR measured from time point 1 to 4 (0–21 min) during the experiments. Adenosine triphosphate (ATP) production was defined as the difference in OCR between the basal respiration and the OCR value after 2.5 μM oligomycin injection. Spare respiratory capacity was defined as the difference in OCR

between the basal respiration and the OCR value after 1 µM FCCP injection. In addition, the metabolic potential was evaluated by calculating the stressed OCR and the stressed ECAR. The stressed OCR was defined as the ratio of the OCR under stressed conditions with 1 µM FCCP to the OCR under normal conditions. We measured the ECAR under normal and stressed conditions with 2.5 µM oligomycin; the stressed ECAR was defined as the ratio of the ECAR under stressed conditions to the ECAR under normal conditions. The hiPSC-CMs in the CM, CM+SF 25%, and CM+SF 50% groups were used in this experiment because 96-well plates should be used for this experiment. The hiPSC-CMs in the CM+MSC group were excluded because co-existence with hMSCs made an accurate measurement in this experiment impossible.

Reactive Oxygen Species (ROS) Measurement

The levels of intracellular ROS were analyzed using the OxiSelect Intracellular ROS Assay Kit (Cell Biolabs, San Diego, CA, USA) according to the manufacturer's instructions after hiPSC-CMs were cultured with or without hMSCs in 24-well plates for 3 days. Briefly, the cells were washed three times with PBS and 200 µl 1× 2′,7′-dichlorodihydrofluorescin diacetate (DCFH-DA) solution was added to the cells, which were incubated at 37 °C for 30 min in light. After washing three times with PBS, the cells were incubated in DMEM with or without 2 µM H_2O_2 for 60 min. The absorbance of the cells was measured at 530 nm on a microplate reader (PowerWave HT; BioTek, Beijing, China). The cells in the CM, CM+MSC, and CM+SF groups were used in this experiment.

Protein Analysis

Enzyme-linked immunosorbent assay (ELISA) kits were used to measure proteins such as hepatocyte growth factor (HGF), stromal cell-derived factor 1 (SDF-1), and vascular endothelial growth factor (VEGF; R&D Systems) secreted from the cultured cells, according to the manufacturers' instructions. The concentration of other proteins secreted from the cultured cells was measured using the Bio-Plex suspension array system (27-plex; Bio-Rad Laboratories) according to the manufacturer's instructions. The media in the CM and CM+MSC groups were used in this experiment.

Recombinant Proteins and Inhibitors

To investigate the impact of each cytokine derived from hMSCs on the maturity of hiPSC-CMs, we used recombinant proteins and inhibitors in several experiments. Recombinant VEGF, recombinant SDF-1, recombinant bFGF, and recombinant

granulocyte-macrophage colony-stimulating factor (GM-CSF, all from R&D Systems) were purchased as recombinant proteins. Anti-VEGF neutralizing antibodies, anti-SDF-1 neutralizing antibodies, anti-bFGF neutralizing antibodies, and anti-GM-CSF neutralizing antibodies (all from R&D Systems) were purchased as blocking antibodies. GW4869 (Sigma-Aldrich, St. Louis, MO, USA) was purchased as an exosome secretion blocker.

Immunoelectron Microscopy

Preparation of exosomes for transmission electron microscopy (TEM) was performed as described previously by Lässer *et al*.[4] After isolation of the exosomes, they were pre-fixed using 2% paraformaldehyde for 10 min. Then, the samples were immunostained with anti-CD63 antibody (Thermo Fisher Scientific) for 40 min and 10 nm-gold labeled secondary antibodies (Abcam) for 40 min. After washing the grid, we post-fixed the samples by incubating the grid with 2.5% glutaraldehyde for 10 min. We contrasted the sample with 2% uranyl acetate for 15 min. After incubating the grid for 10 min with 0.13% methyl cellulose and 0.4% uranyl acetate, the samples were examined under an electron microscope (Hitachi H-7500; Hitachi, Tokyo, Japan). The antibodies used in this study can be found in Table SII.

Particle Size Analysis

Size distribution analysis of the exosomes was performed using the qNano system (Izon Science, Christchurch, New Zealand). After diluting 5 μl of samples in 45 μl of dilution buffer (100 mM KCl (Wako), 10 mM Tris (hydroxymethyl) aminomethane (Wako), 3 mM ethylenediaminetetraacetic acid (Nakalai Tesque), and 0.01% v/v Triton X-100 (MP Biomedicals, Santa Ana, CA, USA)), the size of the exosomes was measured using NP100 nanopores and CPC100B calibration particles and analyzed using Izon Control Suite 3.2 software according to the manufacturer's instructions.

Microarray for MicroRNAs

MicroRNAs were extracted from 1 $\times 10^6$ hiPSC-CMs or exosomes using the mirVana™ miRNA Isolation Kit (Thermo Fisher Scientific) according to the manufacturer’s instructions. The concentration and purity of the extracted RNA were determined using the ND-1000 Spectrophotometer (NanoDrop). Cellular RNA (200 ng) or exosomal RNA (50 ng) were retrotranscribed and pre-amplified according to the manufacturer’s instructions (Thermo Fisher Scientific). Pre-amplified products were loaded onto the TaqMan Array Human MicroRNA A Cards v2.0 (Thermo Fisher Scientific). PCR was then performed using Viia7 Real-Time PCR system. The results of PCR were normalized against *RNU44*, which was

selected as an endogenous control.

Liquid Chromatography Tandem-Mass Spectrometry (LC-MS/MS) Analysis

LC-MS/MS was performed as described previously,[5, 6] with slight modifications. Peptides were extracted from exosomes using MPEX PTS Reagents (GL Sciences Inc., Tokyo, Japan) and were separated through 250-min gradient elution at a flow rate of 250 nl/min with the UltiMate 3000 RSLCnano System (Thermo Fisher Scientific), which was directly interfaced with the Q Exactive Hybrid Quadrupole-Orbitrap Mass Spectrometer (Thermo Fisher Scientific). The Acclaim PepMap RSLC column was used. Mobile phase A consisted of water with 0.1% formic acid, and mobile phase B consisted of methanol with 0.1% formic acid. The Q Exactive Mass Spectrometer was operated in the data-dependent acquisition mode using Xcalibur 2.1.2 software and there was a single full-scan mass spectrum in the orbitrap (350–1500 m/z, 70,000 resolution), followed by 15 data-dependent MS/MS scans at 27% normalized collision energy.

Protein identification was performed using Proteome Discoverer 1.4 software (Thermo Fisher Scientific). The spectra were extracted from raw MS data files and searched against the SwissProt reviewed human proteome database. Precursor Mass Tolerance was 10 ppm, Fragment Mass Tolerance was 0.1 Da and a maximum of two missed cleavages were allowed. Carbamidomethylation (on C) was set as static modification, and oxidation (on M) was set as dynamic modification. Protein identification was considered valid if at least one peptide was statistically significant (with a false discovery rate (FDR) of 1%). Default values were used for all other parameters not mentioned above.

Gene Ontology (GO) Analysis/Pathway Analysis

The software MirPath v.3 from the DIANA tools website (http://snf-515788.vm.okeanos.grnet.gr/) was used to identify potential miRNA target genes and pathways in our study. microT-CDS was used to predict the potential target genes and demonstrate possible relationships between the databases. Pathway analysis was performed to determine the involvement of co-expressed genes in different biological pathways according to the Kyoto Encyclopedia of Genes and Genomes (KEGG). GO analysis was used to investigate the pathways associated with biological processes, cellular components, and specific molecular functions corresponding to the target genes of miRNAs identified by the software microT-CDS. Using the results of LC-MS/MS analysis, functional enrichment analysis was conducted using DAVID Bioinformatics Resources 6.8 online (https://david.ncifcrf.gov/) for the GO and KEGG pathway analyses.

Echocardiography

Echocardiography (Vivid i; GE Healthcare) was performed on rats under general anesthesia using 1% isoflurane just before, 1, 2, and 4 weeks after the treatment procedure. The left ventricular end-systolic dimension (LVESD) and end-diastolic dimension (LVEDD) were obtained from M-mode tracings at the midpapillary level. The left ventricular ejection fraction (LVEF) was calculated as follows: LVEF (%) = $[(LVEDD^3 - LVESD^3) / LVEDD^3]$[4].

Cardiac Catheterization

To assess systolic and diastolic cardiac function, cardiac catheterization was performed on rats under general anesthesia using 1% isoflurane, 4 weeks after the treatment procedure. A MicroTip catheter transducer (SPR-671; Millar Instruments Inc., Houston, TX, USA) and conductance catheters (Unique Medical Co, Osaka, Japan) were placed longitudinally in the left ventricle (LV) from the apex and connected to an Integral 3-signal conditioner-processor (Unique Medical Co.). End-systolic elastance and end-diastolic elastance were determined by transiently compressing the inferior vena cava. Data were recorded as a series of pressure–volume loops, which were analyzed using Integral 3 software (Unique Medical Co.). The maximal and minimal rates of change in LV pressure (dP/dt max and dP/dt min, respectively) were obtained from steady-state beats. After the hemodynamic assessment, the rats were sacrificed through anesthetic overdose and the hearts were removed for further biochemical and histological analyses.

Statistical Analysis

Data are presented as the means with standard error for continuous variables. Continuous variables were examined using the Student *t*-test. The one-way ANOVA test was used to compare the values between more than two groups. When the one-way ANOVA test was significant, group differences were compared using the post hoc Tukey's HSD test. Statistical analyses were performed using JMP®13 (SAS Institute Inc., Cary, NC). Statistical significance was defined as $P < 0.05$.

References

1. Takahashi, K, Tanabe, K, Ohnuki, M, Narita, M, Ichisaka, T, Tomoda, K*, et al.* (2007). Induction of pluripotent stem cells from adult human fibroblasts by defined factors. *Cell* **131**: 861-872.
2. Matsuura, K, Wada, M, Shimizu, T, Haraguchi, Y, Sato, F, Sugiyama, K*, et al.* (2012). Creation of human cardiac cell sheets using pluripotent stem cells. *Biochem Biophys Res Commun* **425**: 321-327.
3. Lee, WY, Wei, HJ, Lin, WW, Yeh, YC, Hwang, SM, Wang, JJ*, et al.* (2011). Enhancement of cell retention and functional benefits in myocardial infarction using human amniotic-fluid stem-cell bodies enriched with endogenous ECM. *Biomaterials* **32**: 5558-5567.
4. Lasser, C, Eldh, M, and Lotvall, J (2012). Isolation and characterization of RNA-containing exosomes. *J Vis Exp*: e3037.
5. Xu, L, Gao, Y, Chen, Y, Xiao, Y, He, Q, Qiu, H*, et al.* (2016). Quantitative proteomics reveals that distant recurrence-associated protein R-Ras and Transgelin predict post-surgical survival in patients with Stage III colorectal cancer. *Oncotarget* **7**: 43868-43893.
6. Jin, L, Huo, Y, Zheng, Z, Jiang, X, Deng, H, Chen, Y*, et al.* (2014). Down-regulation of Ras-related protein Rab 5C-dependent endocytosis and glycolysis in cisplatin-resistant ovarian cancer cell lines. *Mol Cell Proteomics.* 2014;13(11): 3138-3151.
