# Supplementary material for: Maturation of Human Induced Pluripotent Stem Cell-Derived Cardiomyocytes by Soluble Factors from Human Mesenchymal Stem Cells
Source: Mol Ther. 2018 Aug 16;26(11):2681–95. doi: 10.1016/j.ymthe.2018.08.012 (PMC6224789; doi:10.1016/j.ymthe.2018.08.012)
Supplement: Document S2. Article plus Supplemental Information [file mmc9.pdf]

Molecular Therapy
Original Article

Maturation of Human Induced Pluripotent Stem Cell-Derived Cardiomyocytes by Soluble Factors from Human Mesenchymal Stem Cells

Shohei Yoshida,[1] Shigeru Miyagawa,[1] Satsuki Fukushima,[1] Takuji Kawamura,[1] Noriyuki Kashiyama,[1] Fumiya Ohashi,[1] Toshihiko Toyofuku,[2] Koichi Toda,[1] and Yoshiki Sawa[1]

[1]Department of Cardiovascular Surgery, Osaka University Graduate School of Medicine, 2-2 Yamadaoka, Suita, Osaka 565-0871, Japan; [2]Department of Immunology and Regenerative Medicine, Osaka University Graduate School of Medicine, 2-2 Yamadaoka, Suita, Osaka 565-0871, Japan

In this study, we proposed that the functionality or phenotype of differentiated cardiomyocytes derived from human induced pluripotent stem cells (iPSC-CMs) might be modified by co-culture with mesenchymal stem cells (MSCs), resulting in an improved therapeutic potential for failing myocardial tissues. Structural, motility, electrophysiological, and metabolic analyses revealed that iPSC-CMs co-cultured with MSCs displayed aligned myofibrils with A-, H-, and I-bands that could contract and relax quickly, indicating the promotion of differentiation and the establishment of the iPSC-CM structural framework, and showed clear gap junctions and an electric pacing of >2 Hz, indicating enhanced cell-cell interactions. In addition, soluble factors excreted by MSCs, including several cytokines and exosomes, enhanced cardiomyocyte-specific marker production, produced more energy under normal and stressed conditions, and reduced reactive oxygen species production by iPSC-CMs under stressed condition. Notably, gene ontology and pathway analysis revealed that microRNAs and proteins in the exosomes impacted the functionality and maturation of iPSC-CMs. Furthermore, cell sheets consisting of a mixture of iPSC-CMs and MSCs showed longer survival and enhanced therapeutic effects compared with those consisting of iPSC-CMs alone. This may lead to a new type of iPSC-based cardiomyogenesis therapy for patients with heart failure.

INTRODUCTION

Heart failure retains a high global mortality rate, despite marked progress in medical treatments; therefore, it is vital to apply new concepts for developing novel therapeutic alternatives.[1,2] In the past decade, several stem cell therapies including bone marrow progenitor cells, cardiac cells, and somatic stem cells have been explored in clinical settings.[3–7] Unfortunately, their therapeutic effects are limited to the specific region of the heart or to the responding patient, likely because these depend primarily on paracrine effects by the transplanted cells and not on the recovery of functioning cardiomyocytes. Recently, a cardiomyogenesis therapy using cardiomyocytes derived from human induced pluripotent stem cells (hiPSC-CMs) was proposed as a new, alternative candidate therapeutic treatment for several previous stem cell applications.[8–10] However, remaining concerns including poor cell survival or immature cardiomyogenic differentiation, which directly influence therapeutic effects, limit the efficacy of clinical applications.[8,9,11] Conversely, it was reported that hiPSC-derived mature cardiac tissue showed longer survival after orthotopic transplantation.[12] Therefore, strategies to support the transplanted hiPSC-CMs by enhancing their maturity and functionality must be identified.

Several maturation protocols described previously showed that hiPSC-CMs were not matured completely compared with cardiomyocytes in adult hearts, and that substantial time was required to mature the hiPSC-CMs.[13–16] Moreover, Yang et al.[14] described the limitations of using a single factor to induce a complex trait such as maturation. In contrast, human mesenchymal stem cells (hMSCs) have been shown to secrete several soluble factors, which promote the differentiation of other stem or progenitor cells such as neural stem cells or oligodendroglial progenitor cells, and enhance the electrical coupling of hiPSC-CMs.[17–20] On the other hand, one potential reason for poor cell survival may constitute a damaged vascular network at the implanted site.[21,22] Somatic stem cells, such as myoblasts, co-cultured with hMSCs have been reported to enhance cell survival by forming a rich vascular network induced by cytokine secretion; hMSCs can also behave as feeder cells to support co-cultured stem or progenitor cell survival, proliferation, and differentiation.[23–25]

Herein, we hypothesized that hMSC co-culture might modulate hiPSC-CM maturity and functionality *in vitro* and enhance their cell survival and therapeutic potential for treating heart failure following myocardial infarction *in vivo*. We also investigated whether soluble factors secreted from hMSCs could induce hiPSC-CM

Received 23 May 2018; accepted 10 August 2018;
https://doi.org/10.1016/j.ymthe.2018.08.012.

Correspondence: Yoshiki Sawa, MD, PhD, Department of Cardiovascular Surgery, Osaka University Graduate School of Medicine, 2-2 Yamadaoka, Suita, Osaka 565-0871, Japan
E-mail: sawa-p@surg1.med.osaka-u.ac.jp

This is an open access article under the CC BY license (http://creativecommons.org/licenses/by/4.0/).

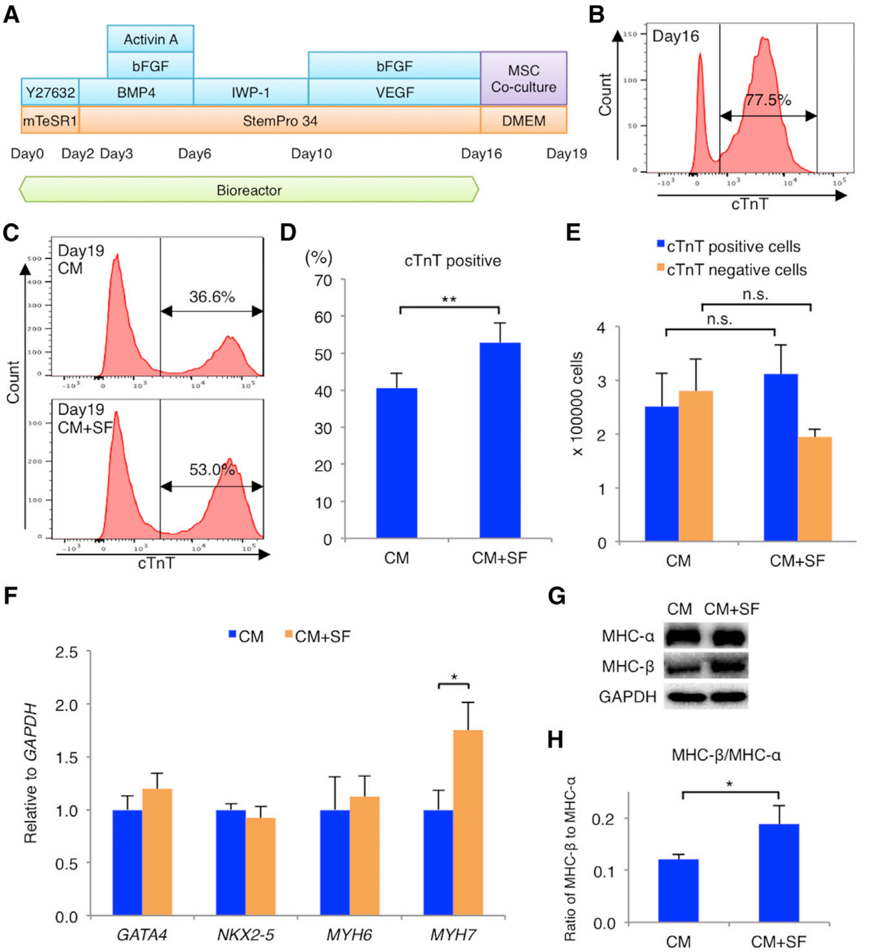

Figure 1. Human Mesenchymal Stem Cells Increase the Population of Cardiac Troponin T-Positive Cells and Promote the Molecular Development of Cardiomyocytes Derived from Human Induced Pluripotent Stem Cells

(A) Cardiomyogenic differentiation protocol and co-culture with human mesenchymal stem cells (hMSCs). (B) Representative flow cytometry data of differentiated human induced pluripotent stem cells (hiPSC-CMs) stained with anti-cardiac troponin T (cTnT) antibodies at day 16. (C) Representative flow cytometry data of differentiated hiPSC-CMs with and without hMSC-derived soluble factors stained with anti-cTnT antibodies at day 19 (CM+SF and CM, respectively). (D) Percentage of cTnT-positive cells in the CM and CM+SF groups as determined by flow cytometry (n = 5 for each group). **p < 0.01, Student t test. (E) Number of cTnT-positive or -negative cells in the CM and CM+SF groups (n = 3 for each group). n.s., not significant, Student t test. (F) Expression of cardiac cell-specific genes (GATA binding protein 4 [*GATA4*], NK2 homeobox 5 [*NKX2-5*], and myosin heavy chain 6 [*MYH6*] and *MYH7*) in CM or CM+SF cells, normalized against *GAPDH* expression (n = 7 for each group). *p < 0.05, Student t test. (G) Western blot of CM or CM+SF cells using anti-myosin heavy chain alpha (MHC-α) antibody, anti-MHC-β antibody, and anti-GAPDH antibodies. (H) Ratio of MHC-β to MHC-α in CM or CM+SF cells as determined by western blotting (n = 4 for each group). *p < 0.05, Student t test. For all experiments, results are shown as mean + SEM. bFGF, basic fibroblast growth factor; BMP4, bone morphogenetic protein 4; VEGF, vascular endothelial growth factor.

maturation, and whether co-culture and co-transplantation with hMSCs could enhance hiPSC-CM cell survival and therapeutic effects.

RESULTS

hMSCs Increase the Cardiac Troponin T-Positive Cell Population and Promote hiPSC-CM Molecular Development

hiPSCs were differentiated into cardiomyocytes using the protocol described in the Materials and Methods (Figure 1A). The obtained cardiomyocyte purity was 76% ± 3%, as assessed by flow cytometry for cardiac troponin T (cTnT) (Figure 1B). After the cardiac differentiation, hiPSC-CMs were cultured alone (CM), or co-cultured with hMSCs (CM+MSC) or hMSC-derived soluble factors (CM+SF) for 3 days in new plate dish, as described in the Materials and Methods (Figure S1). Although the suspension culture using bioreactors did not require a high number of adherent cells, a 3-day plate culture of hiPSC-CMs after the suspension culture increased the number of adherent cells such as fibroblasts, leading to a decrease in the purity of cardiomyocytes. Nevertheless, the cTnT-positive cell proportion was significantly higher in the CM+SF (53% ± 5%) than in the CM group (40% ± 4%; p = 0.0013) (Figures 1C and 1D). In the CM+SF and CM groups, total cell numbers were similar, whereas the cTnT-positive cell number was slightly, albeit not significantly, higher ($3.1 \pm 0.5 \times 10^6$ versus $2.5 \pm 0.6 \times 10^6$ cells; p = 0.2603) and the cTnT-negative cell number ($1.9 \pm 0.2 \times 10^6$ versus $2.8 \pm 0.6 \times 10^6$ cells; p = 0.2571) was slightly lower in the CM+SF than in the CM group (Figure 1E). qRT-PCR analysis (described in the Supplemental Materials and Methods) of known cardiac markers further validated maturation enhancement by co-culture with hMSC soluble factors. The mRNA expression of transcriptional regulators, such as GATA binding protein 4 (*GATA4*) and NK2 homeobox 5 (*NKX2-5*), did not differ significantly between groups. However, hMSC-secreted soluble factors significantly increased relative myosin heavy chain 7 (*MYH7*) mRNA expression (p = 0.0272), whereas that of myosin heavy chain 6 (*MYH6*) did not differ significantly between groups (Figure 1F). Notably, western blotting analysis (described in the Supplemental Materials and Methods) revealed that the myosin heavy chain-β (MHC-β)-to-MHC-α ratio, a known cardiac maturation marker,[11] was significantly higher in the CM+SF (0.19 ± 0.04) group than the CM group (0.12 ± 0.01; p = 0.0455) (Figures 1G and 1H),

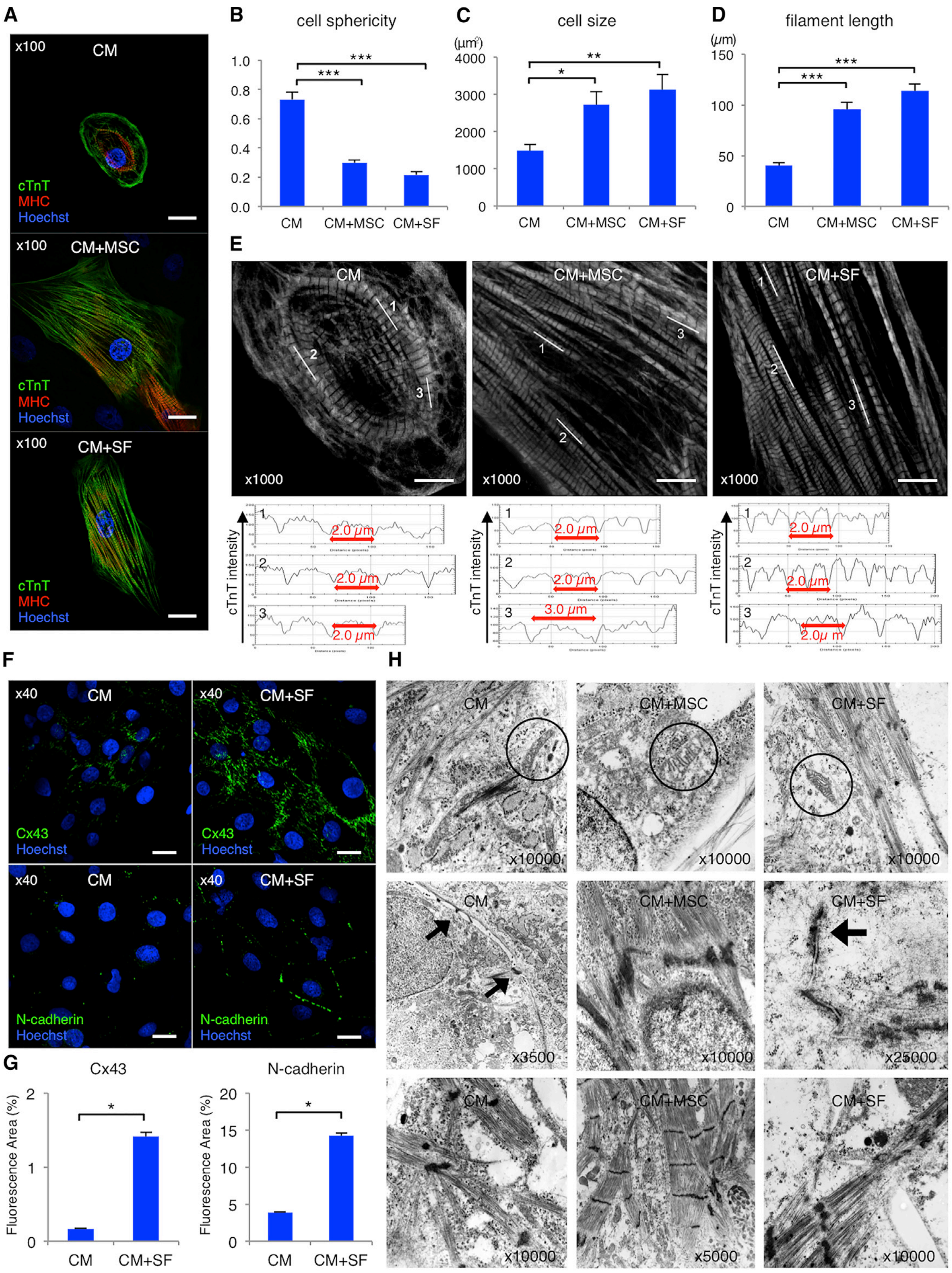

Figure 2. hMSCs Promote Structural Development in hiPSC-CMs

(A) Immunohistochemistry of cardiac troponin T (cTnT; green), myosin heavy chain (MHC; red), and nuclei (Hoechst33258; blue) in differentiated cardiomyocytes (CM), cardiomyocytes co-cultured with mesenchymal stem cells (CM+MSC), and cardiomyocytes cultured with MSC-derived soluble factors (CM+SF). Scale bars: 30 μm. (B–D) Cell sphericity (B), cell size (C), and filament length (D) in the CM, CM+MSC, and CM+SF groups (n = 7 for each group). $^{*}p < 0.05$; $^{**}p < 0.01$; $^{***}p < 0.001$, one-way ANOVA

(legend continued on next page)

consistent with the result of mRNA expression analysis. Thus, hiPSC-CMs induced differentiation more effectively with than without hMSC co-cultivation.

hMSCs Promote hiPSC-CM Structural Development

To evaluate the presence of cardiac-specific components in hiPSC-CMs, we performed immunostaining (detailed in the Supplemental Materials and Methods). Differentiated cardiomyocytes in the CM, CM+MSC, and CM+SF groups were stained with cTnT (green), cardiac MHC (red), and nuclei (Hoechst 33342; blue) (Figure 2A). The CM group (0.73 ± 0.05) exhibited a significantly higher sphericity index than the CM+MSC (0.30 ± 0.02; $p < 0.0001$) and CM+SF groups (0.22 ± 0.02, $p < 0.0001$; ANOVA: $p < 0.0001$) (Figure 2B), but a significantly lower average cell size (1,483 ± 496 versus 2,720 ± 955 μm^2, p = 0.0327 [CM+MSC], and 3,138 ± 1,034 μm^2, p = 0.0042 [CM+SF]; ANOVA: p = 0.0037) (Figure 2C). The filament length was also significantly shorter in the CM (40 ± 9 μm) than in the CM+MSC (96 ± 18 μm; $p < 0.0001$) and CM+SF groups (114 ± 18 μm; $p < 0.0001$) (Figure 2D). Super-resolution microscopic images demonstrated that CM group sarcomeres had an average length of 2.0 μm and did not contain H-bands (Figure 2E), whereas CM+MSC group sarcomeres had the same or greater lengths and contained H-bands, and CM+SF group sarcomeres exhibited 2.0-μm average length in addition to H-bands. These findings indicated that hMSC-derived soluble factors and cell-cell contact with hMSCs might contribute to hiPSC-CM structural alternations.

Connexin 43 (green) or N-cadherin (green) and nuclei (Hoechst 33342; blue) staining images showed higher connexin 43 or N-cadherin expression in the CM+SF group (1.4% ± 0.1% and 14.2% ± 0.3%, respectively) than in the CM group (0.2% ± 0.0%, p = 0.0495, and 3.9% ± 0.1%, p = 0.0495) (Figures 2F and 2G), which might lead to a robust physical and electrical junction in the hiPSC-CMs.

Moreover, transmission electron microscopy (TEM) images of hiPSC-CMs in the CM group showed intersecting immature myofibrils without A- or I-bands, small narrow mitochondria with indistinct cristae, and poor adhesion at the intercellular junction. Conversely, the CM+SF group showed myofibrils with immature A- and I-bands, mitochondria with distinct cristae, and gap junctions and intercalated disks at the intercellular junction. The CM+MSC group showed aligned myofibrils with clear A- and I-bands, mitochondria with more distinct cristae, and a high density of intercalated disks to which actin filaments attached (Figure 2H). Thus, structural analyses indicated that co-cultured hMSCs enhanced hiPSC-CM maturation.

hMSCs Promote hiPSC-CM Motility

To evaluate hiPSC-CM contractility, we performed motion analysis, in which the high-velocity area is red and low-velocity area is blue (Figure 3A; Videos S1, S2, S3, and S4). The CM+SF group had a significantly larger beating area (96% ± 1%) than the CM group (77% ± 2%; $p < 0.0001$) (Figure 3B). Other CM+SF parameters such as acceleration (307 ± 50 versus 153 ± 15 μm/s2; p = 0.0079), contraction velocity (16.8 ± 2.2 versus 7.7 ± 0.6 μm/s; p = 0.0027), and relaxation velocity (11.3 ± 0.9 versus 5.6 ± 0.4 μm/s; $p < 0.0001$) were also significantly higher than those in the CM group (Figures 3C–3E). Thus, hiPSC-CMs co-cultured with hMSCs showed increased contractility.

hMSCs Promote hiPSC-CM Electrophysiological Development

To further investigate the mechanisms underlying the cardiomyocyte performance changes, we performed an intracellular calcium ratiometric dye fluo-8 assay (Figure 3F). The CM+SF 25% (43 ± 1 beats per minute [bpm]) and 50% groups (40 ± 2 bpm) beating rates were slightly lower than the CM group rate (46 ± 3 bpm) (ANOVA: p = 0.1258) (Figure 3G). The CM+SF 50% group had a significantly higher peak ratio (1.044 ± 0.004) than the CM+SF 25% group (1.033 ± 0.001; p = 0.0208) and the CM group (1.031 ± 0.002, p = 0.0170; ANOVA: p = 0.0289) (Figure 3H). The rising slope in the CM+SF 50% (1.6 ± 0.2/ms) group was not significantly higher than that in the CM (1.2 ± 0.2/ms) or CM+SF 25% group (1.3 ± 0.2/ms; ANOVA: p = 0.4904) (Figure 3I). The CM group showed significantly shorter peak width duration (PWD; 317 ± 4 ms) than the CM+SF 25% (386 ± 13 ms; p = 0.0098) and 50% groups (380 ± 18 ms, p = 0.0256; ANOVA: p = 0.0085) (Figure 3J). Additionally, although CM and CM+SF 25% group cardiomyocytes could not achieve an electrical pacing of >2 Hz, CM+SF 50% group cardiomyocytes showed 2.5-Hz electric pacing (Figure 3K). Thus, co-culture with hMSCs promoted electrophysiological development in hiPSC-CMs.

hMSCs Promote hiPSC-CM Metabolic Development

hMSC-derived soluble factors increased the hiPSC-CM oxygen consumption rate (OCR) at every phase in a dose-dependent manner (Figure 4A). The CM+SF 50% group had significantly higher basal respiration (85.6 ± 12.5 pmol/min) and ATP production (70.9 ± 10.0 pmol/min) than the CM group (46.1 ± 3.0 pmol/min, p = 0.0205, and 39.2 ± 2.4 pmol/min, p = 0.0229, respectively), whereas basal respiration and ATP production in the CM+SF 25% group (71.1 ± 14.2 pmol/min, p = 0.1151, and 58.2 ± 11.8 pmol/min, p = 0.1405) were similar to those in the CM group (Figures 4B and 4C). Moreover, the CM+SF 50% (165.6 ± 12.3 pmol/min; p = 0.0002) and 25% groups (121.0 ± 18.8 pmol/min; p = 0.0340) had significantly higher spare respiratory capacity than the CM group

with post hoc Tukey's honestly significant difference (HSD) test. (E) Upper panels display immunohistochemistry of cTnT (white) in the CM, CM+MSC, or CM+SF groups through super-resolution microscopy. Lower panels show the intensity of cTnT at the white lines in the above images. Scale bars: 10 μm. (F) Upper panels show immunohistochemistry of connexin 43 (Cx43; green) and Hoechst33258 (blue) in the CM and CM+SF groups. Lower panels show immunohistochemistry of N-cadherin (green) and nuclei (Hoechst33258; blue) in the CM and CM+SF groups. Scale bars: 20 μm. (G) Percent of fluorescence area, which was stained with Cx43 and N-cadherin, in the CM and CM+SF groups (n = 4 for each group). *$p < 0.05$, Student t test. (H) Transmission electron microscopy images of cardiomyocytes in the CM, CM+MSC, and CM+SF groups. For all experiments, results are shown as mean + SEM.

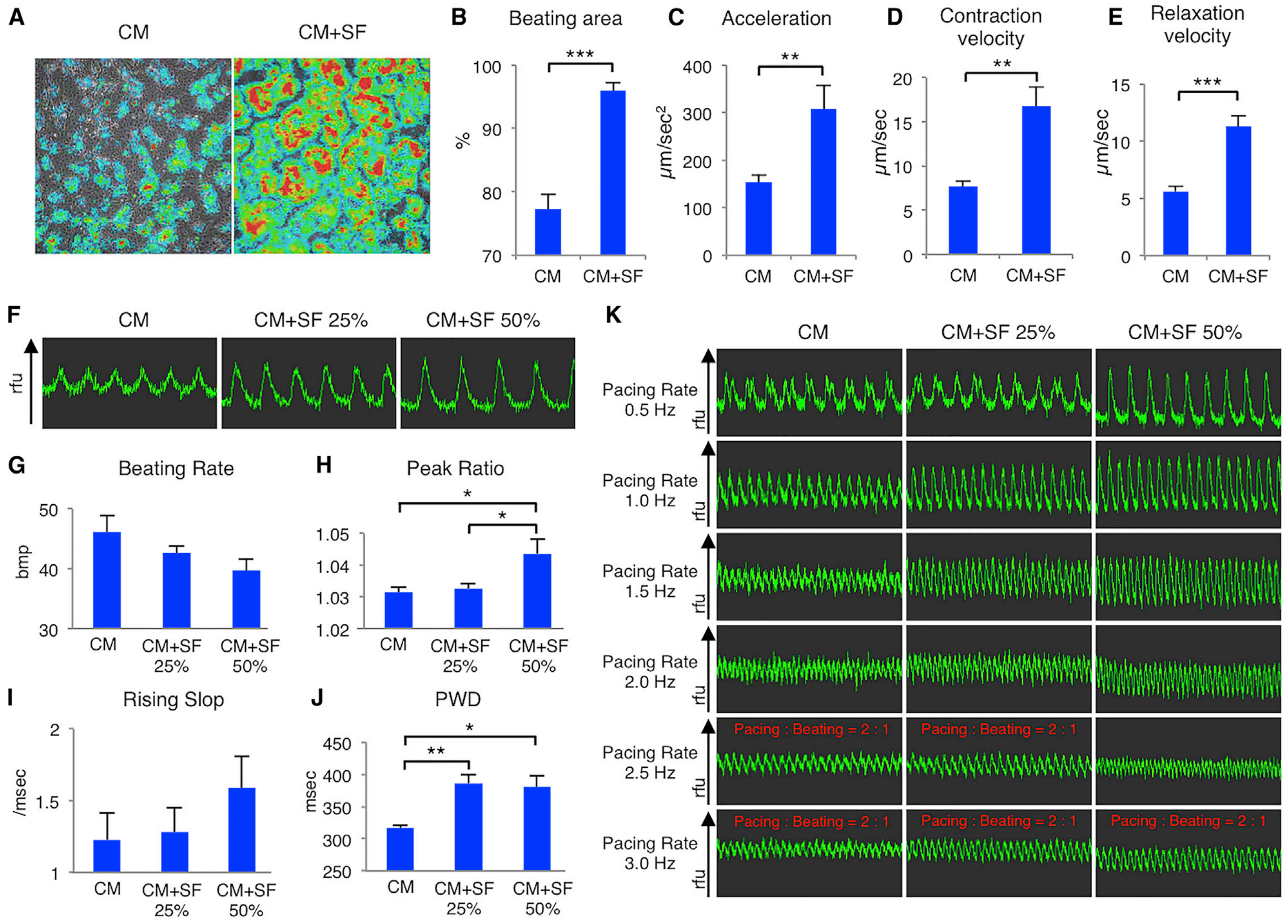

Figure 3. hMSCs Promote Contractile and Electrophysiological Development in hiPSC-CMs

(A) Representative velocity data in differentiated cardiomyocytes (CM; left panel) and cardiomyocytes cultured with MSC-derived soluble factors (CM+SF; right panel) by a motion analysis system. Red and blue represent high and low velocity, respectively. (B–E) Percentage of beating area (B), acceleration (C), contraction velocity (D), and relaxation velocity (E) in the CM and CM+SF groups (n = 4 for each group). **p < 0.01; ***p < 0.001, Student t test. (F) Representative wave forms associated with Ca^{2+} transients in CM, CM with soluble factors secreted from 25% total percentage hMSCs (CM+SF 25%), or CM with soluble factors secreted from 50% total percentage hMSCs (CM+SF 50%), using the FDSS/μCELL system. (G–J) Beating rate (G), peak ratio (H), rising slope (I), or peak width duration (PWD) (J) of cells in the CM, CM+SF 25%, and CM+SF 50% groups as analyzed by FDSS software U8524-12 (n = 6 for each group). *p < 0.05; **p < 0.01, one-way ANOVA with post hoc Tukey's HSD test. (K) Representative wave forms associated with Ca^{2+} transients in CM, CM+SF 25%, and CM+SF 50% cells with a pacing rate of 0.5, 1, 1.5, 2, 2.5, or 3 Hz. For all experiments, results are shown as mean + SEM.

(79.3 ± 3.4 pmol/min) (Figure 4D). Figure 4E shows cellular energy phenotypes of the CM, CM+SF 25%, and CM+SF 50% groups under normal and stressed conditions. Although extracellular acidification rate (ECAR) metabolic potentials did not differ between the three groups (CM: 164% ± 4%; CM+SF 25%: 166% ± 4%; CM+SF 50%: 161% ± 4%; ANOVA: p = 0.6325), the CM group OCR metabolic potential (211% ± 6%) was significantly lower than those of the CM+SF 25% (234% ± 7%; p = 0.0463) and CM+SF 50% groups (238% ± 7%, p = 0.0189; ANOVA: p = 0.0137) (Figure 4F). Thus, hMSC-derived soluble factors enhanced hiPSC-CM mitochondrial energetics.

In addition, oxidative stress increased CM group reactive oxygen species (ROS) levels to 4.6 ± 0.4-fold more than those of normal culture conditions. Conversely, ROS levels were unchanged in the CM+MSC (1.0 ± 0.3-fold increase; p < 0.0001) and the CM+SF (1.1 ± 0.1-fold increase; p < 0.0001) groups under oxidative stress (ANOVA: p < 0.0001) (Figure 4G). Consistent with the release of mitochondrial DNA fragments into the media upon mitochondrial damage, the relative levels of mitochondrial genes, such as *NADH* and *COX3*, were significantly increased in the CM group compared with those of the CM+MSC and CM+SF groups (*NADH*: 1.00 ± 0.02 versus 0.07 ± 0.02, p < 0.0001, versus 0.07 ± 0.02, p < 0.0001; *COX3*: 1.00 ± 0.01 versus 0.08 ± 0.02, p < 0.0001, versus 0.08 ± 0.02, p < 0.0001; ANOVA: p < 0.0001) (Figures 4H and 4I). Stanniocalcin 1 (STC-1), a protein that stabilizes mitochondrial membrane potential, was significantly increased in the medium of the CM+ SF group

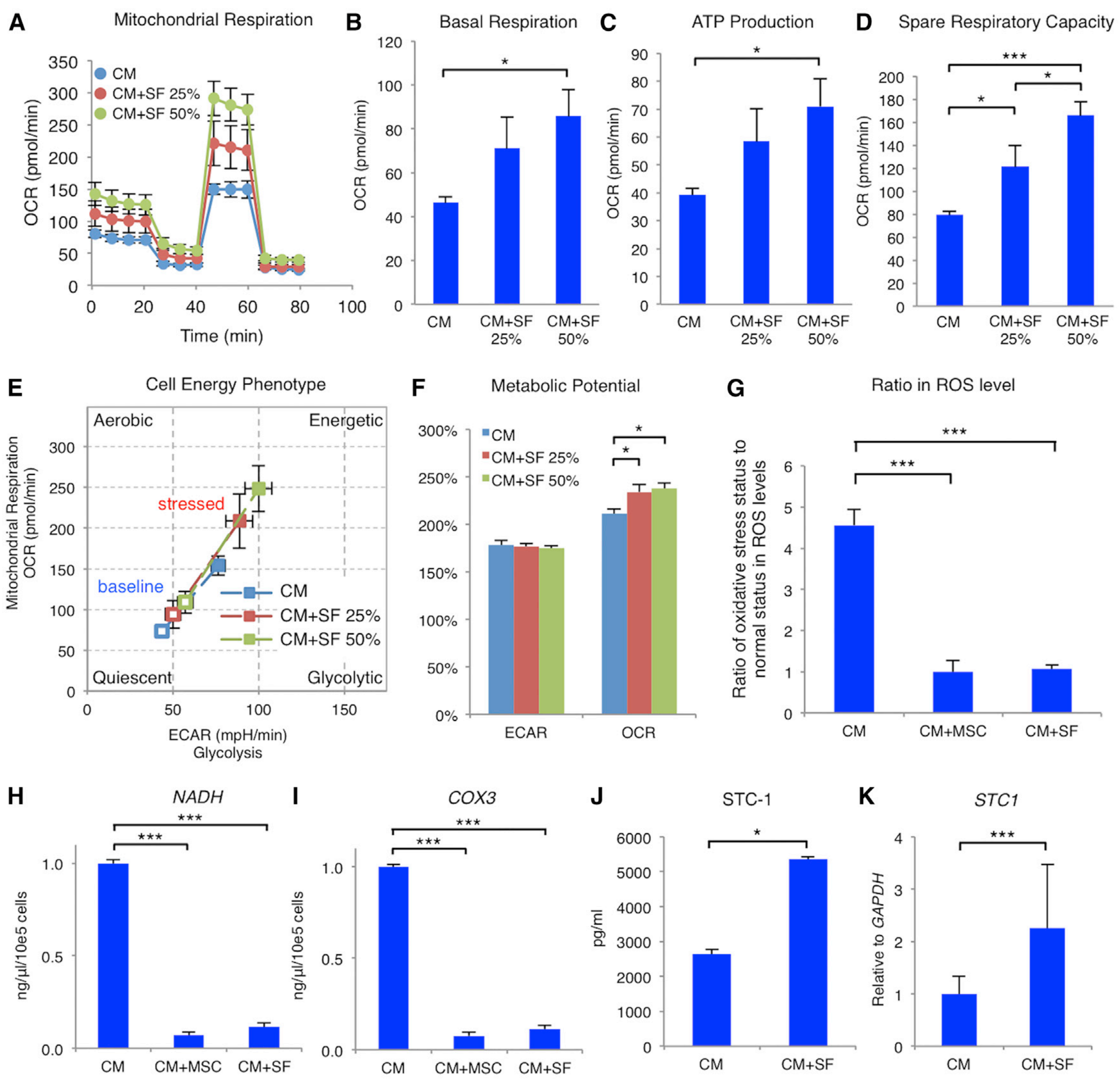

Figure 4. hMSCs Promote Metabolic Development in hiPSC-CMs

(A) Representative mitochondrial respiration in differentiated cardiomyocytes (CM), CM with soluble factors secreted from 25% of hMSCs (CM+SF 25%), or CM with soluble factors secreted from 50% of hMSCs (CM+SF 50%) after incubation with the ATP synthase inhibitor oligomycin, the respiratory uncoupler carbonyl cyanide-p-trifluoromethoxyphenylhydrazone (FCCP), and the respiratory chain blockers rotenone and antimycin A. (B–D) Basal respiration (B), ATP production (C), and spare respiratory capacity (D) of cells in the CM, CM+SF 25%, and CM+SF 50% groups (n = 9 for each group). *$p < 0.05$; ***$p < 0.001$, one-way ANOVA with post hoc Tukey's HSD test. (E) Cell energy phenotype of cells in the CM, CM+SF 25%, and CM+SF 50% groups under normal (outlined shapes) and stressed (solid shapes) conditions (n = 9 for each group). (F) Metabolic potential of glycolysis or mitochondrial respiration in the CM, CM+SF 25%, and CM+SF 50% groups (n = 9 for each group). *$p < 0.05$. (G) Ratio of reactive oxygen species (ROS) levels in cells undergoing oxidative stress compared with normal cells in the CM, CM+MSC, and CM+SF groups (n = 7 for each group). ***$p < 0.001$, one-way ANOVA with post hoc Tukey's HSD test. (H and I) Quantitative analysis of mitochondrial genes (*NADH*, H; *COX3*, I) from media containing CM, CM+MSC, or CM+SF cell culture (n = 4 for each group). ***$p < 0.001$, one-way ANOVA with post hoc Tukey's HSD test. (J) Concentration of stanniocalcin 1 (STC-1) in media containing CM or CM+SF cell culture (n = 3 for each group). *$p < 0.05$, Student t test. (K) Expression of the *STC1* gene in the CM and CM+SF groups, normalized against *GAPDH* expression (n = 7 for each group). ***$p < 0.001$, Student t test. For all experiments, results are shown as mean + SEM. ECAR, extracellular acidification rate; OCR, oxygen consumption rate.

(5,354 ± 75 pg/mL) compared with that in the CM group (2,632 ± 133 pg/mL; p = 0.0478), suggestive of ROS production suppression (Figure 4J). Furthermore, qRT-PCR revealed significantly higher relative hiPSC-CM *STC-1* mRNA expression in the CM+SF group (2.3 ± 1.2; p = 0.0004) than in the CM group (1.0 ± 0.3), suggesting that hMSC-derived soluble factors increased *STC-1* mRNA expression in hiPSC-CMs (Figure 4K). Thus, hiPSC-CMs co-cultured with hMSCs were protected from mitochondria-derived ROS production.

hMSC-Derived Soluble Factors

Next, we examined the mechanisms by which hMSC co-culture affected hiPSC-CMs. To assess hiPSC-CM maturation mechanisms,

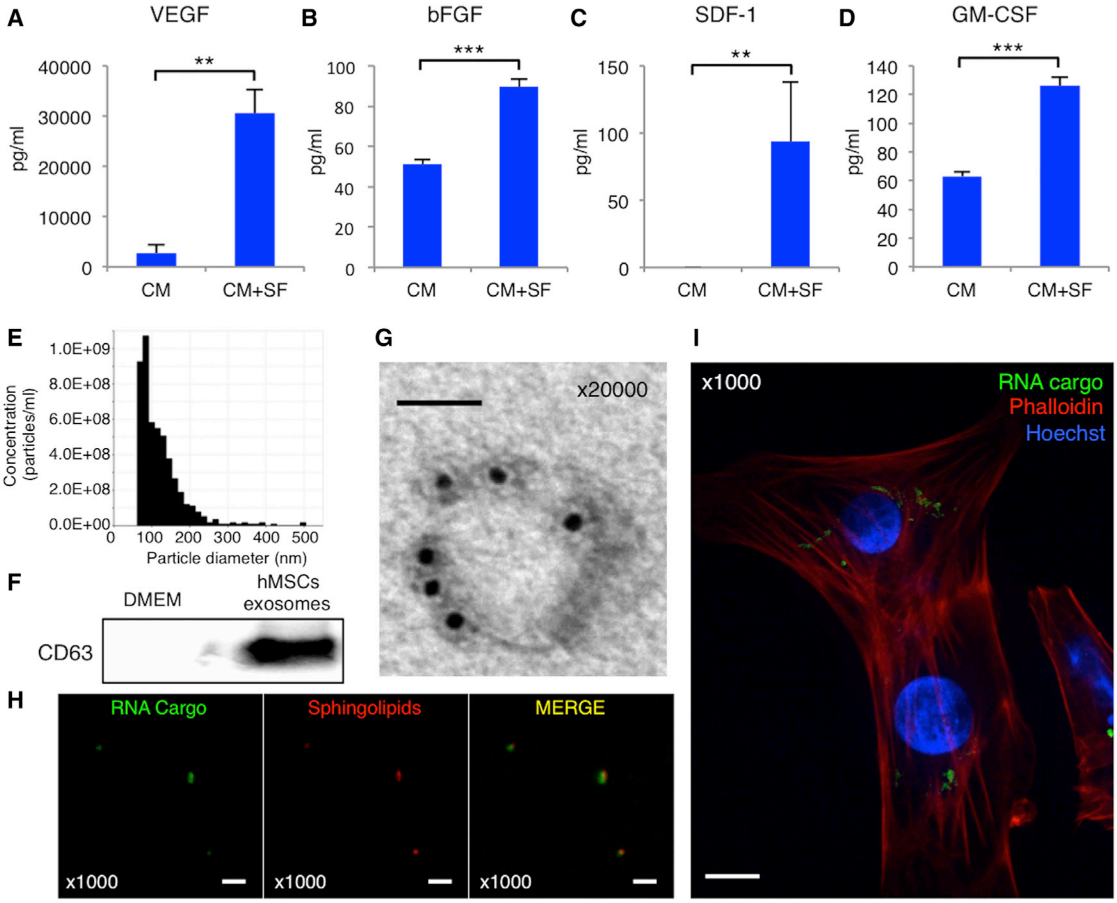

Figure 5. Soluble Factors Derived from hMSCs

(A–D) Concentration of vascular endothelial growth factor (VEGF) (A), basic fibroblast growth factor (bFGF) (B), stromal cell-derived factor (SDF-1) (C), and granulocyte-macrophage colony-stimulating factor (GM-CSF) (D) in differentiated cardiomyocytes (CM) and cardiomyocytes cultured with MSC-derived soluble factors (CM+SF; n = 8 for each group). **p < 0.01; ***p < 0.001, Student t test. (E) Distribution of particle size after ultracentrifugation of media with cultured hMSCs using the qNano system. (F) Representative western blotting data of the exosomes of hMSCs using anti-CD63 antibody. (G) Transmission electron microscopy images of an exosome derived from hMSCs stained with anti-CD63 antibody. Scale bar: 50 nm. (H) RNA (stained green, left) and sphingolipids (stained red, middle) are shown; particles containing both (right) RNA and sphingolipids were considered to be exosomes derived from hMSCs. Scale bars, 2 μm. (I) Exosomes derived from hMSCs stained with RNA cargo (green) were incubated with hiPSC-CMs stained with phalloidin (red) and nuclei (Hoechst33258; blue) 12 hr after addition of the exosomes into the culture media. Scale bar, 10 μm. For all experiments, results are shown as mean + SEM.

we investigated the hMSC-derived soluble factors. Cytokines in the supernatant were measured using Bio-Plex and an ELISA (Figure S2). The CM+SF group had higher vascular endothelial growth factor (VEGF; 30,566 ± 4755 pg/mL), basic fibroblast growth factor (bFGF; 89 ± 4 pg/mL), stromal cell-derived factor 1 (SDF-1; 94 ± 44 pg/mL), and granulocyte-macrophage colony-stimulating factor (GM-CSF; 126 ± 6 pg/mL) concentrations than the CM group (2,737 ± 1,664 pg/mL, p = 0.0016; 51 ± 2 pg/mL, p = 0.0008; 0 ± 0 pg/mL, p = 0.0027; 63 ± 3 pg/mL, p = 0.0008, respectively) (Figures 5A–5D). Additionally, investigation of hMSC-derived exosome contents showed that the majority of particles, which were concentrated using ultracentrifugation, had a diameter of 60–200 nm (Figure 5E) and were positive for CD63, which is specific to extracellular exosomes, by western blotting (Figure 5F). Following anti-CD63 antibody immunostaining, TEM images showed CD63-positive particles with 120-nm diameter (Figure 5G). Therefore, we considered the particles to be exosomes in the following assay. Super-resolution microscopic images revealed that the exosomes have sphingolipids and RNA (Figure 5H). Exosomes, in which the RNA cargo was stained green, were added to culture media containing hiPSC-CMs. Histological analysis revealed that the exosomes were taken into the hiPSC-CM cytosol after 12 hr of incubation (Figure 5I). Thus, co-cultured hMSCs released various bioactive factors for cardiac cells, as well as exosomes transmissible to cardiac cells.

hMSC-Derived Soluble Factors Impact hiPSC-CM Maturity

We investigated the impact of each hMSC-derived soluble factor on hiPSC-CM maturity using recombinant proteins and inhibitors.

Although each recombinant cytokine showed a slightly increased ratio of a known cardiac maturation marker MHC-β to MHC-α (Figure 6A), the overall cytokine ratio did not increase as much as the hMSC-derived soluble factors. Conversely, the ratio decreased upon addition of each blocking antibody or 1 μM GW4869, an exosome secretion blocker. A heatmap generated to present the relative mRNA expression related to cardiac markers (Figure 6B) showed that exosomes impacted mRNA expression rather than cytokines. Regarding metabolic processes, each recombinant cytokine increased the OCR in every phase, with GM-CSF most effective, bFGF and SDF-1 moderately effective, and VEGF least effective. All recombinant cytokines yielded OCR similar to that in the CM+SF group (Figure 6C). Notably, each blocking antibody decreased the OCR in every phase, with GM-CSF most effective, then bFGF, VEGF, and SDF-1 least effective. In particular, bFGF and GM-CSF decreased the ratio more strongly than that in the CM group, likely because the hiPSC-CM-secreted cytokines were blocked (Figure 6D). The hMSC-released exosomes also increased the OCR, which was lowered upon 1 μM GW4869 addition (Figure 6E). Regarding motility, contraction and relaxation velocity were significantly increased by each recombinant cytokine and hMSC-released exosomes, but decreased by each blocking antibody and GW4869. Furthermore, hMSC exosomes influenced both velocities more strongly than any cytokine (Figures 6F and 6G). Thus, released factors, especially hMSC-derived exosomes, could exert beneficial effects on hiPSC-CM performance.

MicroRNAs and Proteins in hMSC Exosomes

We extracted microRNA from exosomes derived from the CM, CM+MSC, and MSC groups. A PCR-based microRNA microarray assay revealed the expression of microRNAs reported to promote cardiomyocyte maturation, including the let7 family, microRNA134, microRNA145, and microRNA296 (Table S3).[15,26–29] These microRNAs were found at higher levels in hMSC-derived than in hiPSC-CM-derived exosomes (Figure 7A). Cardiomyocyte-specific microRNA levels, including microRNA1, microRNA133, microRNA208, and microRNA499,[30–35] were lower in hMSC-derived than in hiPSC-CM-derived exosomes. Such microRNA expression was higher in CM+MSC than in CM group exosomes. Next, we extracted microRNA from hiPSC-CMs with and without the addition of hMSC-derived soluble factors. CM+SF group cells showed higher expression of all of the above microRNAs than the CM group cells (Figure 7B).

Target prediction was performed using microT-CDS and DIANA mirPath v.3. Table S4 provides a detailed list of the identified target genes. Gene ontology enrichment analysis revealed that these genes were markedly enriched in the "cellular nitrogen compound metabolic process," "ion binding," "cell junction organization," "extracellular matrix organization," "cell adhesion," "intrinsic apoptotic signaling pathway," "positive regulation of muscle cell differentiation," "histone acetylation," "insulin-like growth factor receptor signaling pathway," and "transforming growth factor beta (TGF-β) receptor signaling pathway" (Figure 7C). In addition, pathway analysis of the target genes of these microRNAs revealed that pathways associated with adrenergic signaling in cardiomyocytes and the TGF-β, FoxO, AMP-activated protein kinase (AMPK), Wnt, Ras, cyclic guanosine monophosphate-protein kinase G (cGMP-PKG), and Rap1 signaling pathways were upregulated (Figure 7D).

Proteomics analysis of the proteins extracted from the hMSC-derived exosomes identified 598 gene products (Table S5). Functional enrichment analysis of 463 of the gene products, which were classified as products found in extracellular exosomes, revealed marked enrichment in the Wnt signaling pathway, autophagy, muscle contraction, angiogenesis, responses to calcium ions, cell-cell junctions, and actin filaments (Figure 7E). Additionally, pathway analysis of these gene products revealed substantial enrichment of gap junctions, cyclic AMP (cAMP) signaling pathway, and adrenergic signaling in cardiomyocytes (Figure 7F).

hiPSC-CM and hMSC Combination Enhanced Therapeutic Effects *In Vivo*

We performed cell sheets transplantation onto the hearts of athymic nude rats in a 2-week-old myocardial infarction model. Immunohistochemical analysis of cell sheets consisting of hiPSC-CM (CM sheet) or CM+MSC sheets (MIX sheet) revealed higher connexin 43 expression in the MIX sheet than in the CM sheet (Figure S3). Survival of the transplanted cells from the MIX sheets was observed 4 weeks after sheet transplantation through immunohistochemical images (Figure 8A), whereas transplanted cells from the other sheets were not detected at this time point. PCR showed that rats that had received MIX sheet transplantation had significantly higher human *GAPDH* levels, measured using total DNA extracted from whole heart samples (4.3 ± 2.3), than those receiving other sheets (sham: 0.3 ± 0.0, p = 0.0187; CM sheet: 1.0 ± 0.2, p = 0.0144; MSC sheet: 0.9 ± 0.2, p = 0.0190; Figure 8B). Figure 8C shows the relative left ventricular ejection fractions compared with sham rats through serial echocardiography. MIX sheet rats exhibited greater relative ejection fraction (19.4% ± 3.5%) than MSC sheet rats (6.5% ± 1.7%; p = 0.0033) and sham rats (0.0% ± 2.5%; p = 0.0007) at 1 week (ANOVA: p = 0.0004), 2 weeks (21.6% ± 3.9% [MIX sheet] versus 9.7% ± 1.3% [CM sheet], p = 0.0098, versus 5.2% ± 1.9% [MSC sheet], p = 0.0003, versus 0.0% ± 3.1% [sham], p < 0.0001; ANOVA: p < 0.0001), and 4 weeks after sheet transplantation (20.9% ± 3.8% [MIX sheet] versus 8.8% ± 1.5% [CM sheet], p = 0.0112; 3.6% ± 2.6% [MSC sheet], p = 0.0002, versus 0.0% ± 2.5% [sham], p < 0.0001; ANOVA: p < 0.0001).

Cardiac catheterization, performed to assess systolic and diastolic cardiac function,[36,37] showed significantly higher maximal rate of change in left ventricular pressure (dP/dt) in the MIX sheet (5,120 ± 400 mm Hg) than in the sham (2,782 ± 428 mm Hg; p = 0.0028) and MSC sheet groups (3,646 ± 392 mm Hg; p = 0.0150), and was slightly, but not significantly, higher than in the CM sheet group (4,906 ± 233 mm Hg; p = 0.7296; Figure 8D). The MIX sheet (−3,362 ± 384 mm Hg; p = 0.0351) and CM sheet groups (−3,701 ± 223 mm Hg; p = 0.0177) had significantly lower minimum dP/dt than the sham group (−1,974 ± 364 mm Hg;

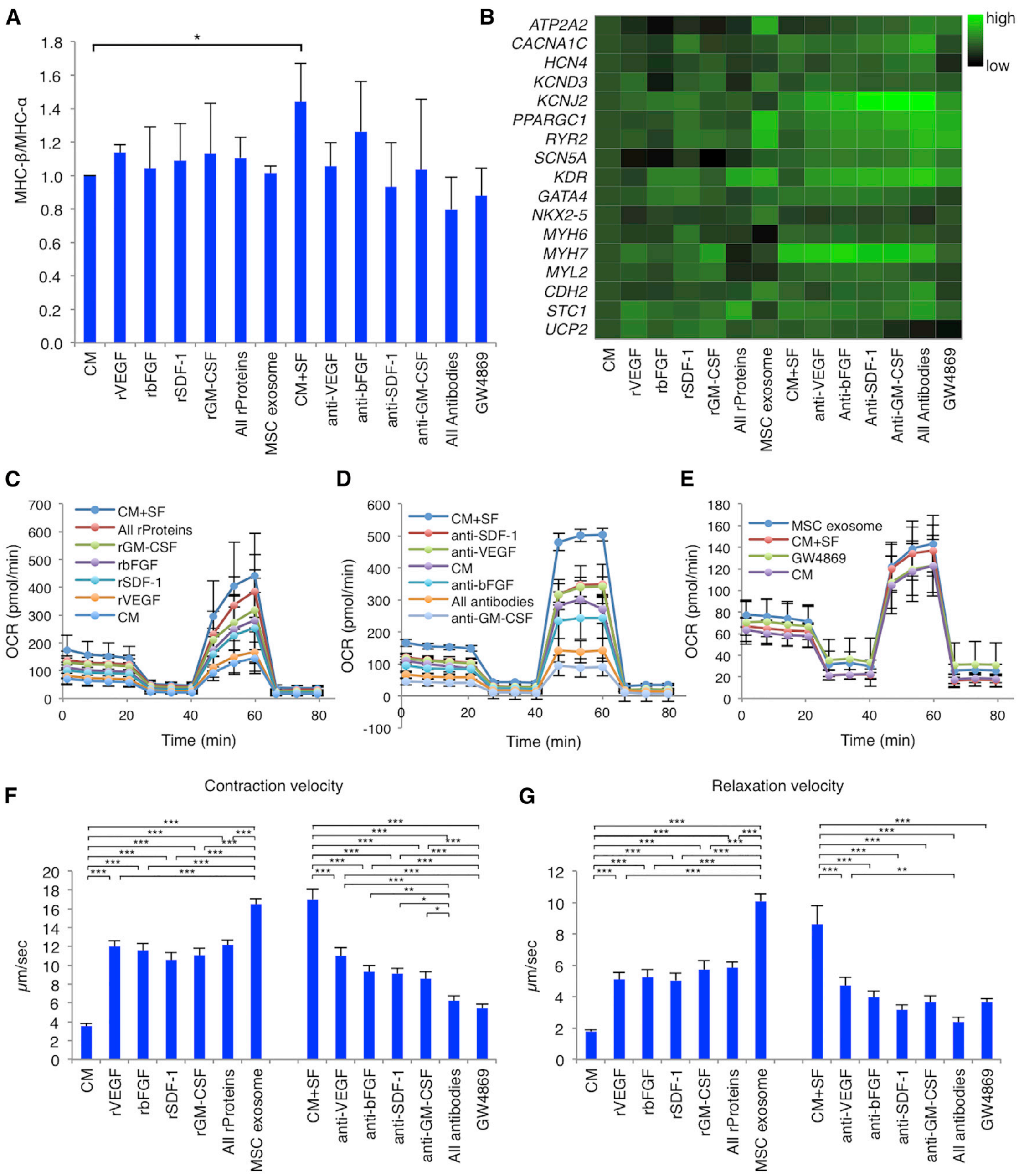

Figure 6. Impact of the Soluble Factors Derived from hMSCs on the Maturity of hiPSC-CMs

Recombinant vascular endothelial growth factor (rVEGF), recombinant basic fibroblast growth factor (rbFGF), recombinant stromal cell-derived factor 1 (rSDF-1), recombinant granulocyte-macrophage colony-stimulating factor (rGM-CSF), all four recombinant proteins (all rProteins), or hMSC exosomes (MSC exosome) were added to culture media containing hiPSC-CMs (CM). Anti-VEGF neutralizing antibody (anti-VEGF), anti-bFGF neutralizing antibody (anti-bFGF), anti-SDF-1 neutralizing antibody (anti-SDF-1), anti-GM-CSF neutralizing antibody (anti-GM-CSF), all four neutralizing antibodies (all antibodies), or GW4879 were also added to culture media containing hiPSC-CMs with hMSC-derived soluble factors. (A) Ratio of myosin heavy chain (MHC)-β to MHC-α in all groups (n = 4 for each group). *$p < 0.05$, one-way ANOVA with post hoc Tukey's HSD test. (B) Heatmap regarding expression of the cardiac genes in all groups, normalized against *GAPDH* expression (n = 9 for each group). (C) Representative mitochondrial respiration rates in the CM, rVEGF, rbFGF, rSDF-1, rGM-CSF, all rProteins, and CM+SF groups. (D) Representative mitochondrial respiration rates in the CM, anti-VEGF, anti-bFGF, anti-SDF-1, anti-GM-CSF, all antibodies, and CM+SF groups. (E) Representative mitochondrial respiration rates in the CM, MSC exosome, GW4879, and CM+SF groups. (F and G) Contraction velocity (F) or relaxation velocity (G) in all groups (n = 5 for each group). *$p < 0.05$; **$p < 0.01$; ***$p < 0.001$, one-way ANOVA with post hoc Tukey's HSD test. For all experiments, results are shown as mean + SEM. OCR, oxygen consumption rate.

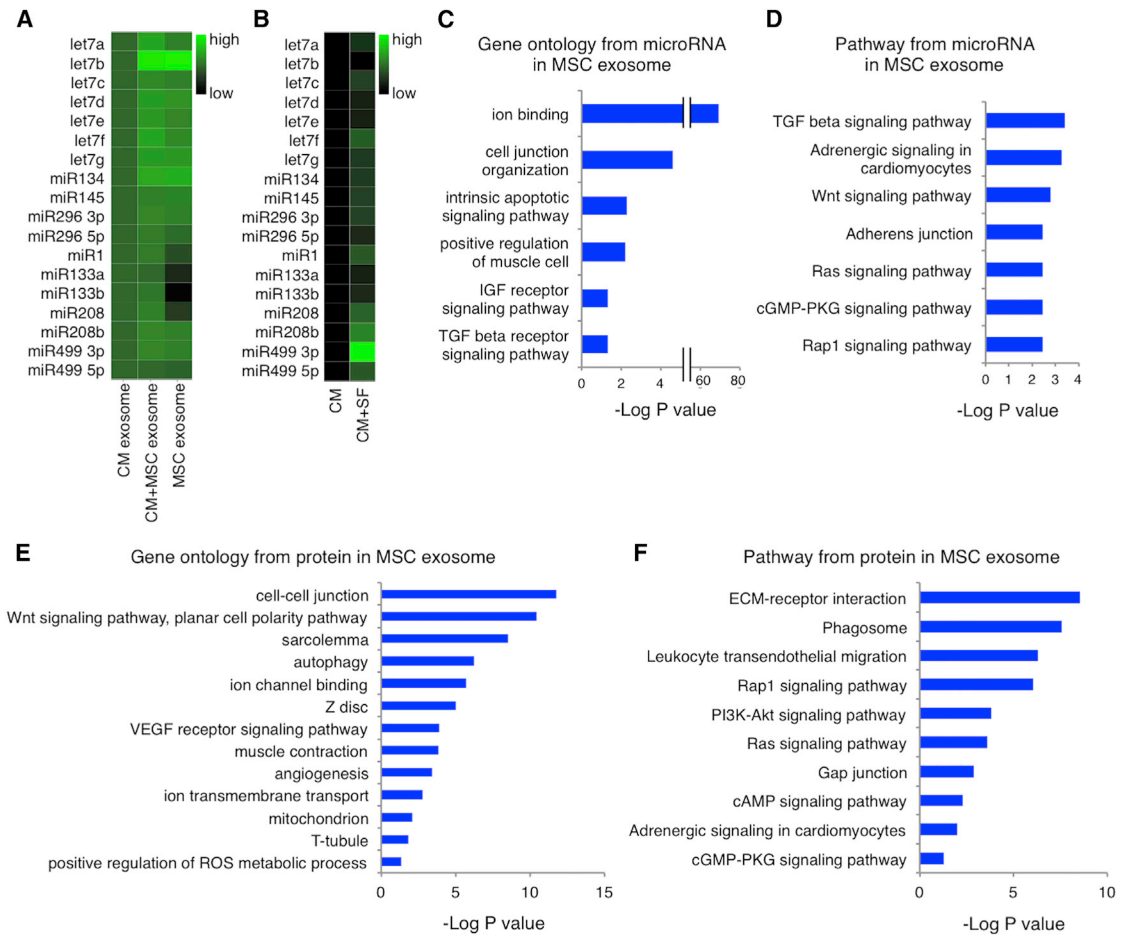

Figure 7. MicroRNAs and Proteins in hMSC-Derived Exosomes

(A and B) Expression of microRNAs reported to promote the maturation of cardiomyocytes (let7 family, microRNA134, microRNA145, microRNA296) or to be specific to cardiomyocytes (microRNA1, microRNA133, microRNA208, microRNA499) in exosomes from culture media containing differentiated cardiomyocytes (CM), cardiomyocytes co-cultured with MSCs (CM+MSC), or MSC (A); and in CM or cardiomyocytes cultured with MSC-derived soluble factors (CM+SF) (B). (C and D) Gene ontology (C) and pathway analysis (D) of the microRNAs associated with the maturation of cardiomyocytes. The vertical axes show the gene ontology category and pathway category, respectively, and the horizontal axes show the extent of enrichment of each gene ontology category or pathway, respectively. (E and F) Gene ontology (E) and pathway analysis (F) of proteins in hMSC exosomes. The vertical axes show the gene ontology category and the pathway category, respectively, and the horizontal axes show the extent of enrichment of each gene ontology category or pathway, respectively.

Figure 8E). The MIX sheet group had significantly higher end-systolic elastance (1,492 ± 45 mm Hg/mL) than other groups (sham: 526 ± 269 mm Hg/mL, p = 0.0053; CM: 836 ± 106 mm Hg/mL, p = 0.0255; MSC: 780 ± 170 mm Hg/mL, p = 0.0096; Figure 8F). The MIX sheet group had slightly lower end-diastolic elastance (46 ± 12 mm Hg/mL) than the other groups (sham: 99 ± 17 mm Hg/mL, p = 0.0802; CM sheet: 50 ± 22 mm Hg/mL, p = 0.8726; MSC sheet: 73 ± 12 mm Hg/mL, p = 0.3909; Figure 8G).

Additionally, the MIX sheet group fibrotic area in recipient cardiac tissue (11% ± 1%), which was positively associated with the region of myocardial infarction, was smaller than those of the sham (17% ± 2%; p = 0.0068) and MSC sheet groups (17% ± 1%; p = 0.0028; Figure 8H). The MIX sheet group capillary density (301 ± 6/field) was higher than those of the sham (187 ± 15/field; p < 0.0001), CM sheet (253 ± 7/field; p = 0.0028), and MSC sheet groups (228 ± 12/field; p < 0.0001; Figure 8I).

qRT-PCR analysis revealed that the MIX sheet group displayed higher relative hepatocyte growth factor (HGF; 3.8 ± 1.1) expression than the sham group (1.0 ± 0.2; p = 0.0107) and the CM sheet group (1.4 ± 0.4, p = 0.0235; Figure 8J). Although the expressions of *SDF-1* and *VEGF* were not different between the CM sheet, MSC sheet, and MIX sheet groups, the MIX sheet group showed a relatively higher SDF-1 (6.7 ± 1.6) and VEGF (5.1 ± 1.0) expression than the sham group (1.0 ± 0.3; p = 0.0007; Figure 8K; and 1.0 ± 0.3, p = 0.0011; Figure 8L, respectively). Thus, the MIX sheet could not only maintain cardiac properties *in vivo*, but also improved the functionality of transplanted hearts.

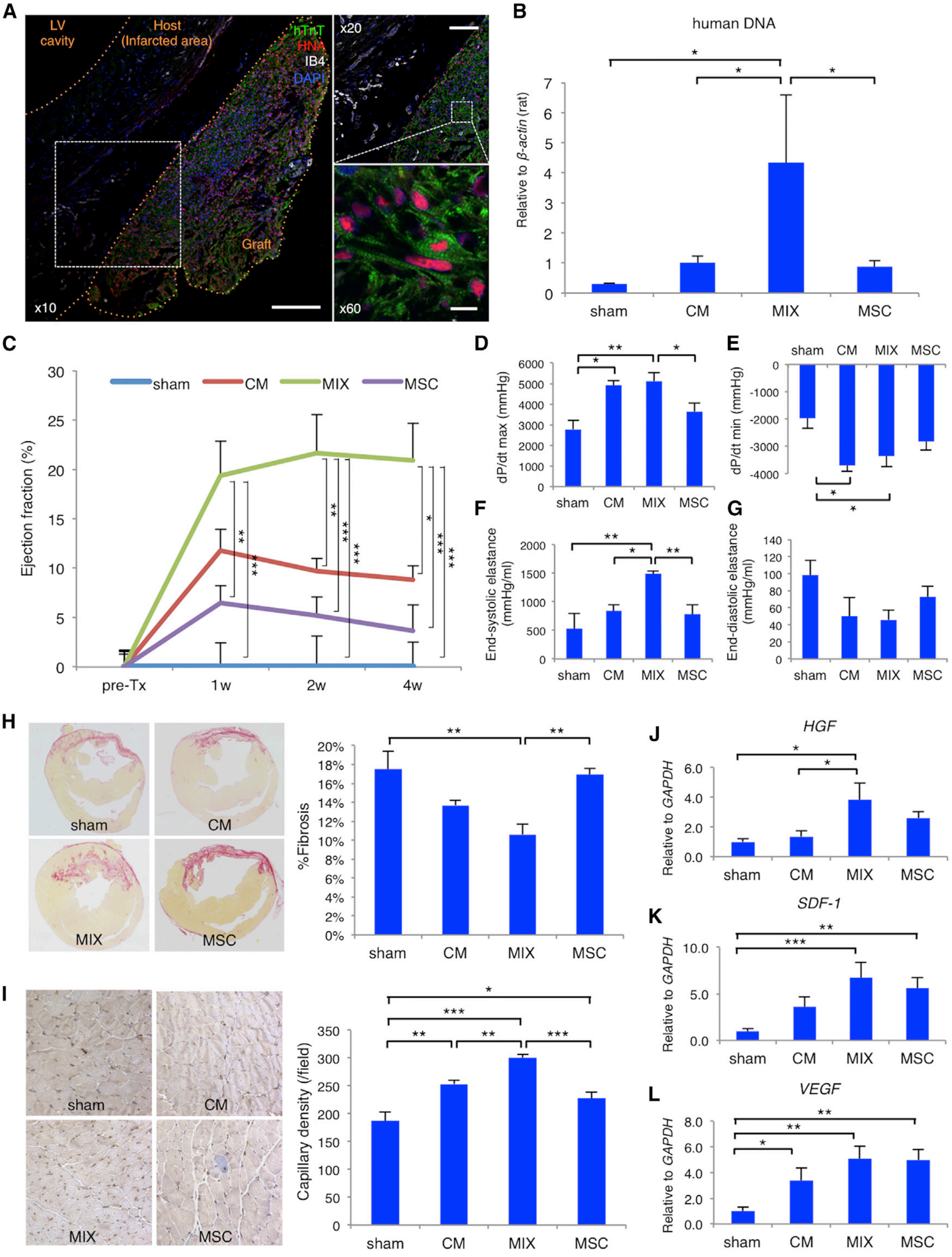

Figure 8. Combination of hiPSC-CMs and hMSCs Enhanced Therapeutic Effects *In Vivo*

(A) Immunohistochemistry of human troponin T (hTnT; green), human nuclei (HNA; red), isolectin B4 (IB4; white), and nuclei (DAPI; blue) in a rat transplanted with a cell sheet containing both differentiated cardiomyocytes and MSCs (MIX) 4 weeks after cell sheet transplantation. Scale bars: 200 μm (left); 100 μm (right, top); 10 μm (right, bottom). (B) Expression of human DNA in the whole hearts of rat given no transplant (sham) or transplanted with a CM sheet, a MIX sheet, or an MSC sheet, normalized against rat *β-actin* expression. (C) Serial changes in the relative left ventricular ejection fraction in each group compared with sham rats, analyzed by transthoracic echocardiography (n = 10 for

(legend continued on next page)

DISCUSSION

The major finding of this study was that hMSC co-culture enhanced hiPSC-CM functionality in structural, motility, electrophysiological, and metabolic aspects. This conclusion is supported by the following experiments. hMSC co-culture allowed hiPSC-CMs to: (1) increase cardiomyocyte purity; (2) increase the MHC-β-to-MHC-α ratio; (3) develop a rod-shaped morphology; (4) have fully formed mitochondria and aligned myofibrils with A-, H-, and I-bands; (5) produce more energy under normal and stressed conditions; and (6) reduce ROS production under oxidative stress. Thus, hMSC co-culture promoted iPSC-CM differentiation and enhanced myofibril maturation. Furthermore, hMSC co-culture allowed hiPSC-CMs to: (7) have clear gap junctions, (8) increase connexin 43 and N-cadherin expression, (9) contract and relax quickly, and (10) have an electric pacing of >2 Hz. Thus, hMSC co-culture promoted the iPSC-CM structural framework and enhanced cell-cell interactions. Finally, cell sheets consisting of a mixture of hiPSC-CMs and hMSCs showed longer survival and enhanced therapeutic effects after transplantation into athymic nude rats in a 2-week-old myocardial infarction model, compared with cell sheets consisting of hiPSC-CMs without hMSCs. Collectively, hMSC co-culture promoted iPSC-CM maturation and survival.

Following hiPSC establishment in 2007, they were expected to become a cell source for therapeutic applications such as regenerative medicine, disease modeling, drug screening, and toxicity testing.[38–41] The efficiency of cardiomyocyte differentiation from hiPSCs has been greatly improved in recent years,[38] with many reports describing over 60% cardiomyocytes in differentiated cultures. These cardiomyocytes exhibit sarcomeres, calcium transients, and spontaneous beating, but display a low degree of maturation based on the studied parameters.[42,43] A general consensus has emerged that maturation protocols must be developed to maximize hiPSC-CM therapeutic applications. In the present study, hMSC co-culture for 3 days, using a relatively simple method, affected various parameters representing the degree of cardiomyocyte maturity in adult hearts.

Although hMSC co-culture enhanced iPSC-CM maturation, the specific underlying mechanisms remain unclear. The enhancement of hiPSC-CM maturation caused by hMSC co-culture was observed even in a transwell assay, indicating that hMSC-secreted soluble factors comprised the primary cause of the mechanism. However, it was unclear which factor promoted differentiation and maturation. The present study revealed that hMSCs release cytokines such as VEGF, bFGF, SDF-1, and GM-CSF that are capable of modulating hiPSC-CM functionality. Notably, cytokines that have not been previously used in differentiation protocols for hiPSC-CMs, such as GM-CSF and SDF-1, had a greater influence on hiPSC-CM respiratory capacity than those used in current differentiation methods, such as VEGF and bFGF.

Furthermore, the present study demonstrated that hiPSC-CM functionality was also affected by the exosomes released from hMSCs. Exosomes are small vesicles, 30–150 nm in diameter, containing a wide range of functional proteins and microRNAs. Recent studies have revealed that exosomes play an important role in cell-to-cell communication as intercellular messengers.[44,45] We demonstrated that exosomes released from hMSCs included microRNAs previously shown to promote cardiomyocyte maturation.[15,26–29] Additionally, gene ontology and pathway analysis revealed that the microRNAs and the proteins in the hMSC-released exosomes impacted hiPSC-CM functionality and maturation. These results suggested that hiPSC-CM maturation could not be induced by a single factor, but rather by multiple factors that modulate a wide variety of genes or pathways.

hMSC co-culture promoted hiPSC-CM survival, although the mechanisms controlling this remain unclear. The transplanted cells are insufficiently supported by a vascular network of native myocardial cells, resulting in cellular stress.[21] The angiogenic potential of hMSCs might contribute to the longer hiPSC-CM survival in ischemic areas. This study revealed that hMSC co-culture allowed hiPSC-CMs to produce more energy by aerobic respiration under stressed conditions and to suppress ROS production induced by oxidative stress. In general, ROS play important roles in the regulation of cell survival; a sharp increase in ROS can induce cell death.[46,47] The suppression of ROS production by hMSCs could reduce cell death around the transplanted site *in vivo*. Moreover, hMSC co-culture allowed hiPSC-CMs to have clear gap junctions and to increase connexin 43 and N-cadherin expression. Gap junction channels allow the intercellular passage of small molecules and regulate essential processes,[48] and intercellular communication through gap junctions plays vital roles in cell differentiation and survival.[49] In addition, N-cadherin is essential for cell-cell contact in cardiomyocytes *in vivo* and *in vitro*,[50] with N-cadherin adhesion playing important roles in cardiomyocyte differentiation and survival.[51] Moreover, hMSCs may promote the formation of new intercalated disc-like structures between implanted and host cells, resulting in the synchronous beating of implanted hiPSC-CMs and host cells.

There were a few limitations to this study. The experiments in this study were performed using only a single cell line. Another cell line may yield different results; nevertheless, new concepts concerning maturation or transplantation methods were suggested through this

each group). pre-Tx, pre-transplantation; w, weeks after transplantation. (D–G) The dP/dt max (D), dP/dt min (E), end-systolic elastance (F), and end-diastolic elastance (G) in each group, analyzed by cardiac catheterization. (H) Left panels display representative images of myocardial fibrosis in each group, as assessed by Sirius Red staining. Right graph shows the percentage of fibrotic to myocardial tissue in each group (n = 8 for each group). (I) Left panels display representative images of immunohistochemistry of von Willebrand factor in each group. Right graph shows the capillary density per unit area in each group (n = 8 for each group). (J–L) Expression of the hepatocyte growth factor (*HGF*) (J), stromal cell-derived factor 1 (*SDF-1*) (K), or vascular endothelial growth factor (*VEGF*) (L) genes in each group, normalized against *GAPDH* expression (n = 8 for each group). For all experiments, one-way ANOVA with post hoc Tukey's HSD test is used, and results are shown as mean + SEM. *p < 0.05; **p < 0.01; ***p < 0.001.

research. In addition, too many factors may be associated with hiPSC-CM maturation to clarify each factor precisely, although hMSC-derived soluble factors caused hiPSC-CM maturation. Some factors might simultaneously contribute to the hiPSC-CM maturation in some aspects, but not in other aspects. The differentiation or maturation methods of hiPSC-CMs require further comprehensive analyses. Overall, however, the generated matured hiPSC-CMs may be useful for regenerative medicine, as well as disease modeling, drug screening, and toxicity testing. On the other hand, the regulation and quality control in both cells might be more complex than those in only hiPSC-CMs, when using this maturation method in a clinical setting.

In conclusion, this study provides a proof-of-concept and useful baseline data for future research aimed at elucidating the mechanisms underlying these morphological and functional changes. Co-culture with hMSCs was clearly shown to modulate the maturity and functionality of hiPSC-CMs *in vitro* and to enhance the survival and therapeutic potential of hiPSC-CMs for heart failure following myocardial infarction *in vivo*.

MATERIALS AND METHODS

hMSC Culture

A population of hMSCs from human bone marrow was purchased from Lonza (Basel, Switzerland) and maintained in MSC basal media (Lonza). hMSCs at P4 to P6 were used for all experiments in this study.

Cardiac Differentiation of hiPSCs

Cardiomyogenic differentiation from hiPSC line 253G1 was induced using a previously reported bioreactor system (Figure 1A).[39,52] Details are given in the Supplemental Materials and Methods (see Cardiac Differentiation of Human Induced Pluripotent Stem Cells section).

Co-culture with hMSCs

hiPSC-CMs were cultured on new dishes with the same number of hMSCs (CM+MSC) or without hMSCs (CM) for 3 days after differentiation in DMEM high glucose (Thermo Fisher Scientific, Waltham, MA, USA). To assess the effects of hMSC-secreted soluble factors, we also co-cultured hiPSC-CMs and hMSCs without direct cell-cell contact using Transwell inserts (3.0-μm pore polycarbonate membrane; Corning, Armonk, NY, USA) for 3 days; hMSCs were removed before assay performance (CM+SF) (Figure S1). Because the wells in 96-well plates were too small to culture equivalent hMSC numbers, 40,000 hiPSC-CMs were co-cultured therein with 20,000 (CM+SF 50%) or 10,000 hMSCs (CM+SF 25%). For all other experiments, hiPSC-CMs were co-cultured with equivalent hMSC numbers per plate.

Cell Sheet Preparation and Transplantation

In temperature-responsive culture dishes (UpCell; CellSeed, Tokyo, Japan),[25] we prepared three types of cell sheets as follows: (1) 1×10^6 hiPSC-CMs (CM sheet), (2) 1×10^6 hiPSC-CMs with 1×10^6 hMSCs (MIX sheet), and (3) 1×10^6 hMSCs (MSC sheet). Each cell sheet, or no sheet (sham), was transplanted and attached by several sutures onto the anterior wall of the left ventricle of athymic nude rats (F344/NJcl-rnu/rnu, 7 weeks old, male, 120–130 g; CLEA Japan, Osaka, Japan) 2 weeks after permanent ligation of the proximal site of the left anterior descending artery. Animal care procedures were consistent with the *Guide for the Care and Use of Laboratory Animals* (NIH). Experimental protocols were approved by the Ethics Review Committee for Animal Experimentation of Osaka University Graduate School of Medicine (reference no. 25-025-045).

Isolation of Exosomes from Cell Culture Media

Conditioned media were collected from cells grown in serum-free media for 48 hr. Dead cells and contaminating cell debris were removed by centrifugation at $300 \times g$ for 10 min and then at $2{,}000 \times g$ for 10 min at 4°C. Media were subjected to ultracentrifugation (SW32Ti, Ultra-Clear tube; Beckman Coulter, Brea, CA, USA) at $175{,}000 \times g$ for 120 min at 4°C; ultracentrifugation of the resulting pellet was repeated following washing with PBS to produce a pellet containing extracellular vesicles including exosomes.

SUPPLEMENTAL INFORMATION

Supplemental Information includes Supplemental Materials and Methods, three figures, five tables, and four videos and can be found with this article online at https://doi.org/10.1016/j.ymthe.2018.08.012.

AUTHOR CONTRIBUTIONS

S.Y. designed research studies, conducted experiments, acquired data, analyzed data, and wrote the manuscript. S.M. designed research studies, obtained funding, and revised the manuscript. S.F., T.K., and N.K. designed research studies and searched literature. F.O. conducted experiments, acquired data, and analyzed data. T.T. designed research studies, searched literature, and revised the manuscript. K.T. designed research studies and searched literature. Y.S. designed research studies, obtained funding, and approved the article.

ACKNOWLEDGMENTS

We thank Seiko Eiraku, Akima Harada, and Atsuko Wakimura for their technical support. This research was supported by The Japan Agency for Medical Research and Development project (Research Center Network for Realization of Regenerative Medicine) under grant 17bm0204003h0005.

REFERENCES

1. Menasche, P. (2011). Cardiac cell therapy: lessons from clinical trials. J. Mol. Cell. Cardiol. *50*, 258–265.

2. Behfar, A., Crespo-Diaz, R., Terzic, A., and Gersh, B.J. (2014). Cell therapy for cardiac repair—lessons from clinical trials. Nat. Rev. Cardiol. *11*, 232–246.

3. Malliaras, K., Makkar, R.R., Smith, R.R., Cheng, K., Wu, E., Bonow, R.O., Marbán, L., Mendizabal, A., Cingolani, E., Johnston, P.V., et al. (2014). Intracoronary cardiosphere-derived cells after myocardial infarction: evidence of therapeutic regeneration in the final 1-year results of the CADUCEUS trial (CArdiosphere-Derived aUtologous stem CElls to reverse ventricUlar dySfunction). J. Am. Coll. Cardiol. *63*, 110–122.

4. Schächinger, V., Erbs, S., Elsässer, A., Haberbosch, W., Hambrecht, R., Hölschermann, H., Yu, J., Corti, R., Mathey, D.G., Hamm, C.W., et al.; REPAIR-AMI Investigators

(2006). Intracoronary bone marrow-derived progenitor cells in acute myocardial infarction. N. Engl. J. Med. *355*, 1210–1221.

5. Tendera, M., Wojakowski, W., Ruzyłło, W., Chojnowska, L., Kepka, C., Tracz, W., Musiałek, P., Piwowarska, W., Nessler, J., Buszman, P., et al.; REGENT Investigators (2009). Intracoronary infusion of bone marrow-derived selected CD34+CXCR4+ cells and non-selected mononuclear cells in patients with acute STEMI and reduced left ventricular ejection fraction: results of randomized, multi-centre Myocardial Regeneration by Intracoronary Infusion of Selected Population of Stem Cells in Acute Myocardial Infarction (REGENT) Trial. Eur. Heart J. *30*, 1313–1321.

6. Miyagawa, S., Domae, K., Yoshikawa, Y., Fukushima, S., Nakamura, T., Saito, A., Sakata, Y., Hamada, S., Toda, K., Pak, K., et al. (2017). Phase I clinical trial of autologous stem cell-sheet transplantation therapy for treating cardiomyopathy. J. Am. Heart Assoc. *6*, e003918.

7. Yoshida, S., Miyagawa, S., Toda, K., Domae, K., and Sawa, Y. (2018). Skeletal myoblast sheet transplantation enhanced regional improvement of cardiac function. Eur. Heart J. Cardiovasc. Imaging *19*, 828–829.

8. Kawamura, M., Miyagawa, S., Miki, K., Saito, A., Fukushima, S., Higuchi, T., Kawamura, T., Kuratani, T., Daimon, T., Shimizu, T., et al. (2012). Feasibility, safety, and therapeutic efficacy of human induced pluripotent stem cell-derived cardiomyocyte sheets in a porcine ischemic cardiomyopathy model. Circulation *126* (11 Suppl 1), S29–S37.

9. Kawamura, M., Miyagawa, S., Fukushima, S., Saito, A., Miki, K., Ito, E., Sougawa, N., Kawamura, T., Daimon, T., Shimizu, T., et al. (2013). Enhanced survival of transplanted human induced pluripotent stem cell-derived cardiomyocytes by the combination of cell sheets with the pedicled omental flap technique in a porcine heart. Circulation *128* (11 Suppl 1), S87–S94.

10. Hsiao, L.C., Carr, C., Chang, K.C., Lin, S.Z., and Clarke, K. (2013). Stem cell-based therapy for ischemic heart disease. Cell Transplant. *22*, 663–675.

11. Yang, X., Pabon, L., and Murry, C.E. (2014). Engineering adolescence: maturation of human pluripotent stem cell-derived cardiomyocytes. Circ. Res. *114*, 511–523.

12. Li, J., Minami, I., Shiozaki, M., Yu, L., Yajima, S., Miyagawa, S., Shiba, Y., Morone, N., Fukushima, S., Yoshioka, M., et al. (2017). Human pluripotent stem cell-derived cardiac tissue-like constructs for repairing the infarcted myocardium. Stem Cell Reports *9*, 1546–1559.

13. Hazeltine, L.B., Simmons, C.S., Salick, M.R., Lian, X., Badur, M.G., Han, W., Delgado, S.M., Wakatsuki, T., Crone, W.C., Pruitt, B.L., and Palecek, S.P. (2012). Effects of substrate mechanics on contractility of cardiomyocytes generated from human pluripotent stem cells. Int. J. Cell Biol. *2012*, 508294.

14. Yang, X., Rodriguez, M., Pabon, L., Fischer, K.A., Reinecke, H., Regnier, M., Sniadecki, N.J., Ruohola-Baker, H., and Murry, C.E. (2014). Tri-iodo-l-thyronine promotes the maturation of human cardiomyocytes-derived from induced pluripotent stem cells. J. Mol. Cell. Cardiol. *72*, 296–304.

15. Kuppusamy, K.T., Jones, D.C., Sperber, H., Madan, A., Fischer, K.A., Rodriguez, M.L., Pabon, L., Zhu, W.Z., Tulloch, N.L., Yang, X., et al. (2015). Let-7 family of microRNA is required for maturation and adult-like metabolism in stem cell-derived cardiomyocytes. Proc. Natl. Acad. Sci. USA *112*, E2785–E2794.

16. Lundy, S.D., Zhu, W.Z., Regnier, M., and Laflamme, M.A. (2013). Structural and functional maturation of cardiomyocytes derived from human pluripotent stem cells. Stem Cells Dev. *22*, 1991–2002.

17. Rivera, F.J., Couillard-Despres, S., Pedre, X., Ploetz, S., Caioni, M., Lois, C., Bogdahn, U., and Aigner, L. (2006). Mesenchymal stem cells instruct oligodendrogenic fate decision on adult neural stem cells. Stem Cells *24*, 2209–2219.

18. Bai, L., Lennon, D.P., Eaton, V., Maier, K., Caplan, A.I., Miller, S.D., and Miller, R.H. (2009). Human bone marrow-derived mesenchymal stem cells induce Th2-polarized immune response and promote endogenous repair in animal models of multiple sclerosis. Glia *57*, 1192–1203.

19. Jadasz, J.J., Kremer, D., Göttle, P., Tzekova, N., Domke, J., Rivera, F.J., Adjaye, J., Hartung, H.P., Aigner, L., and Küry, P. (2013). Mesenchymal stem cell conditioning promotes rat oligodendroglial cell maturation. PLoS ONE *8*, e71814.

20. Rubach, M., Adelmann, R., Haustein, M., Drey, F., Pfannkuche, K., Xiao, B., Koester, A., Udink ten Cate, F.E., Choi, Y.H., Neef, K., et al. (2014). Mesenchymal stem cells and their conditioned medium improve integration of purified induced pluripotent stem cell-derived cardiomyocyte clusters into myocardial tissue. Stem Cells Dev. *23*, 643–653.

21. Shudo, Y., Miyagawa, S., Ohkura, H., Fukushima, S., Saito, A., Shiozaki, M., Kawaguchi, N., Matsuura, N., Shimizu, T., Okano, T., et al. (2014). Addition of mesenchymal stem cells enhances the therapeutic effects of skeletal myoblast cell-sheet transplantation in a rat ischemic cardiomyopathy model. Tissue Eng. Part A *20*, 728–739.

22. Sekiya, N., Matsumiya, G., Miyagawa, S., Saito, A., Shimizu, T., Okano, T., Kawaguchi, N., Matsuura, N., and Sawa, Y. (2009). Layered implantation of myoblast sheets attenuates adverse cardiac remodeling of the infarcted heart. J. Thorac. Cardiovasc. Surg. *138*, 985–993.

23. Majumdar, M.K., Thiede, M.A., Mosca, J.D., Moorman, M., and Gerson, S.L. (1998). Phenotypic and functional comparison of cultures of marrow-derived mesenchymal stem cells (MSCs) and stromal cells. J. Cell. Physiol. *176*, 57–66.

24. Richards, M., Fong, C.Y., Chan, W.K., Wong, P.C., and Bongso, A. (2002). Human feeders support prolonged undifferentiated growth of human inner cell masses and embryonic stem cells. Nat. Biotechnol. *20*, 933–936.

25. Okura, H., Matsuyama, A., Lee, C.M., Saga, A., Kakuta-Yamamoto, A., Nagao, A., Sougawa, N., Sekiya, N., Takekita, K., Shudo, Y., et al. (2010). Cardiomyoblast-like cells differentiated from human adipose tissue-derived mesenchymal stem cells improve left ventricular dysfunction and survival in a rat myocardial infarction model. Tissue Eng. Part C Methods *16*, 417–425.

26. Xu, N., Papagiannakopoulos, T., Pan, G., Thomson, J.A., and Kosik, K.S. (2009). MicroRNA-145 regulates OCT4, SOX2, and KLF4 and represses pluripotency in human embryonic stem cells. Cell *137*, 647–658.

27. Wu, Y.-H., Zhao, H., Zhou, L.-P., Zhao, C.-X., Wu, Y.-F., Zhen, L.-X., Li, J., Ge, D.X., Xu, L., Lin, L., et al. (2015). miR-134 modulates the proliferation of human cardiomyocyte progenitor cells by targeting meis2. Int. J. Mol. Sci. *16*, 25199–25213.

28. Tay, Y., Zhang, J., Thomson, A.M., Lim, B., and Rigoutsos, I. (2008). MicroRNAs to Nanog, Oct4 and Sox2 coding regions modulate embryonic stem cell differentiation. Nature *455*, 1124–1128.

29. Li, R., Yan, G., Li, Q., Sun, H., Hu, Y., Sun, J., and Xu, B. (2012). MicroRNA-145 protects cardiomyocytes against hydrogen peroxide (H_2O_2)-induced apoptosis through targeting the mitochondria apoptotic pathway. PLoS ONE *7*, e44907.

30. Cordes, K.R., and Srivastava, D. (2009). MicroRNA regulation of cardiovascular development. Circ. Res. *104*, 724–732.

31. Liu, N., and Olson, E.N. (2010). MicroRNA regulatory networks in cardiovascular development. Dev. Cell *18*, 510–525.

32. Liu, N., Bezprozvannaya, S., Williams, A.H., Qi, X., Richardson, J.A., Bassel-Duby, R., and Olson, E.N. (2008). microRNA-133a regulates cardiomyocyte proliferation and suppresses smooth muscle gene expression in the heart. Genes Dev. *22*, 3242–3254.

33. Zhao, Y., Samal, E., and Srivastava, D. (2005). Serum response factor regulates a muscle-specific microRNA that targets Hand2 during cardiogenesis. Nature *436*, 214–220.

34. Wilson, K.D., Hu, S., Venkatasubrahmanyam, S., Fu, J.-D., Sun, N., Abilez, O.J., Baugh, J.J., Jia, F., Ghosh, Z., Li, R.A., et al. (2010). Dynamic microRNA expression programs during cardiac differentiation of human embryonic stem cells: role for miR-499. Circ. Cardiovasc. Genet. *3*, 426–435.

35. Nishimura, Y., Kondo, C., Morikawa, Y., Tonomura, Y., Torii, M., Yamate, J., and Uehara, T. (2015). Plasma miR-208 as a useful biomarker for drug-induced cardiotoxicity in rats. J. Appl. Toxicol. *35*, 173–180.

36. Stekelenburg-de Vos, S., Steendijk, P., Ursem, N.T., Wladimiroff, J.W., and Poelmann, R.E. (2007). Systolic and diastolic ventricular function in the normal and extra-embryonic venous clipped chicken embryo of stage 24: a pressure-volume loop assessment. Ultrasound Obstet. Gynecol. *30*, 325–331.

37. Frederick, J.R., Fitzpatrick, J.R., 3rd, McCormick, R.C., Harris, D.A., Kim, A.Y., Muenzer, J.R., Marotta, N., Smith, M.J., Cohen, J.E., Hiesinger, W., et al. (2010). Stromal cell-derived factor-1alpha activation of tissue-engineered endothelial progenitor cell matrix enhances ventricular function after myocardial infarction by inducing neovasculogenesis. Circulation *122* (*Suppl 11*), S107–S117.

38. Mummery, C.L., Zhang, J., Ng, E.S., Elliott, D.A., Elefanty, A.G., and Kamp, T.J. (2012). Differentiation of human embryonic stem cells and induced pluripotent stem cells to cardiomyocytes: a methods overview. Circ. Res. *111*, 344–358.

39. Takahashi, K., Tanabe, K., Ohnuki, M., Narita, M., Ichisaka, T., Tomoda, K., and Yamanaka, S. (2007). Induction of pluripotent stem cells from adult human fibroblasts by defined factors. Cell *131*, 861–872.

40. Yu, J., Vodyanik, M.A., Smuga-Otto, K., Antosiewicz-Bourget, J., Frane, J.L., Tian, S., Nie, J., Jonsdottir, G.A., Ruotti, V., Stewart, R., et al. (2007). Induced pluripotent stem cell lines derived from human somatic cells. Science *318*, 1917–1920.

41. Yoshida, Y., and Yamanaka, S. (2011). iPS cells: a source of cardiac regeneration. J. Mol. Cell. Cardiol. *50*, 327–332.

42. Ribeiro, M.C., Tertoolen, L.G., Guadix, J.A., Bellin, M., Kosmidis, G., D'Aniello, C., Monshouwer-Kloots, J., Goumans, M.J., Wang, Y.L., Feinberg, A.W., et al. (2015). Functional maturation of human pluripotent stem cell derived cardiomyocytes in vitro—correlation between contraction force and electrophysiology. Biomaterials *51*, 138–150.

43. Robertson, C., Tran, D.D., and George, S.C. (2013). Concise review: maturation phases of human pluripotent stem cell-derived cardiomyocytes. Stem Cells *31*, 829–837.

44. Bian, S., Zhang, L., Duan, L., Wang, X., Min, Y., and Yu, H. (2014). Extracellular vesicles derived from human bone marrow mesenchymal stem cells promote angiogenesis in a rat myocardial infarction model. J. Mol. Med. (Berl.) *92*, 387–397.

45. Mineo, M., Garfield, S.H., Taverna, S., Flugy, A., De Leo, G., Alessandro, R., and Kohn, E.C. (2012). Exosomes released by K562 chronic myeloid leukemia cells promote angiogenesis in a Src-dependent fashion. Angiogenesis *15*, 33–45.

46. Trachootham, D., Lu, W., Ogasawara, M.A., Nilsa, R.D., and Huang, P. (2008). Redox regulation of cell survival. Antioxid. Redox Signal. *10*, 1343–1374.

47. Navarro-Yepes, J., Burns, M., Anandhan, A., Khalimonchuk, O., del Razo, L.M., Quintanilla-Vega, B., Pappa, A., Panayiotidis, M.I., and Franco, R. (2014). Oxidative stress, redox signaling, and autophagy: cell death versus survival. Antioxid. Redox Signal. *21*, 66–85.

48. Krysko, D.V., Leybaert, L., Vandenabeele, P., and D'Herde, K. (2005). Gap junctions and the propagation of cell survival and cell death signals. Apoptosis *10*, 459–469.

49. Ahir, B.K., and Pratten, M.K. (2014). Structure and function of gap junction proteins: role of gap junction proteins in embryonic heart development. Int. J. Dev. Biol. *58*, 649–662.

50. Zuppinger, C., Eppenberger-Eberhardt, M., and Eppenberger, H.M. (2000). N-Cadherin: structure, function and importance in the formation of new intercalated disc-like cell contacts in cardiomyocytes. Heart Fail. Rev. *5*, 251–257.

51. Soh, B.S., Buac, K., Xu, H., Li, E., Ng, S.Y., Wu, H., Chmielowiec, J., Jiang, X., Bu, L., Li, R.A., et al. (2014). N-cadherin prevents the premature differentiation of anterior heart field progenitors in the pharyngeal mesodermal microenvironment. Cell Res. *24*, 1420–1432.

52. Matsuura, K., Wada, M., Shimizu, T., Haraguchi, Y., Sato, F., Sugiyama, K., Konishi, K., Shiba, Y., Ichikawa, H., Tachibana, A., et al. (2012). Creation of human cardiac cell sheets using pluripotent stem cells. Biochem. Biophys. Res. Commun. *425*, 321–327.

YMTHE, Volume *26*

Supplemental Information

Maturation of Human Induced Pluripotent Stem Cell-Derived Cardiomyocytes by Soluble Factors from Human Mesenchymal Stem Cells

Shohei Yoshida, Shigeru Miyagawa, Satsuki Fukushima, Takuji Kawamura, Noriyuki Kashiyama, Fumiya Ohashi, Toshihiko Toyofuku, Koichi Toda, and Yoshiki Sawa

SUPPLEMENTAL INFORMATION

Figure S1. Co-culture of hiPSC-CMs and hMSCs.
Figure S2. Concentration of Cytokines in Culture Media.
Figure S3. Immunohistochemistry of Cell Sheets.
Table S1. Lists of Primers Used in This Study.
Table S2. Lists of Primary and Secondary Antibodies Used in This Study.
Table S3. Microarray for MicroRNAs in hiPSC-CMs or Exosomes in Culture Media.
Table S4: Target Prediction with Gene Expression from Identified MicroRNAs in Exosomes.
Table S5. Proteomics of MSC Exosomes.
Movie S1. Representative Video of hiPSC-CMs in the CM Group without Color.
Movie S2. Representative Video of hiPSC-CMs in the CM Group with Color.
Movie S3. Representative Video of hiPSC-CMs in the CM+SF Group without Color.
Movie S4. Representative Video of hiPSC-CMs in the CM+SF Group with Color.
Supplemental Materials and Methods
References

SUPPLEMENTAL FIGURE

Figure S1. Co-culture of hiPSC-CMs and hMSCs.

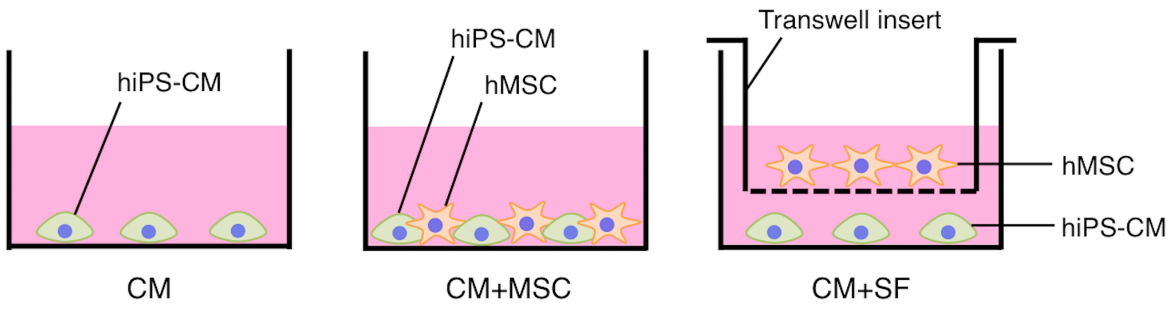

hiPSC-CMs were cultured on new dishes with the same number of hMSCs (CM+MSC) or without hMSCs (CM) for three days after differentiation in Dulbecco's modified Eagle's medium high glucose. hiPSC-CMs and hMSCs were also co-cultured without direct cell-cell contact using Transwell inserts (3.0-µm pore polycarbonate membrane) for three days; hMSCs were removed before assay performance (CM+SF).

Figure S2. Concentration of Cytokines in Culture Media.

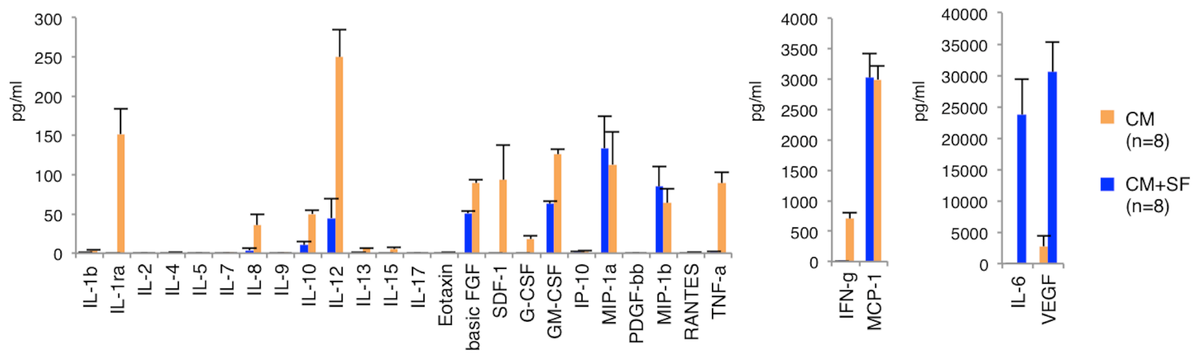

The concentration of each cytokine in culture media containing differentiated cardiomyocytes (CM) and cardiomyocytes cultured with mesenchymal stem cells derived soluble factors (CM+SF; n = 8, for each group), analyzed by an enzyme-linked immunosorbent assay (ELISA) kit and the Bio-plex suspension array system.

Figure S3. Immunohistochemistry of Cell Sheets.

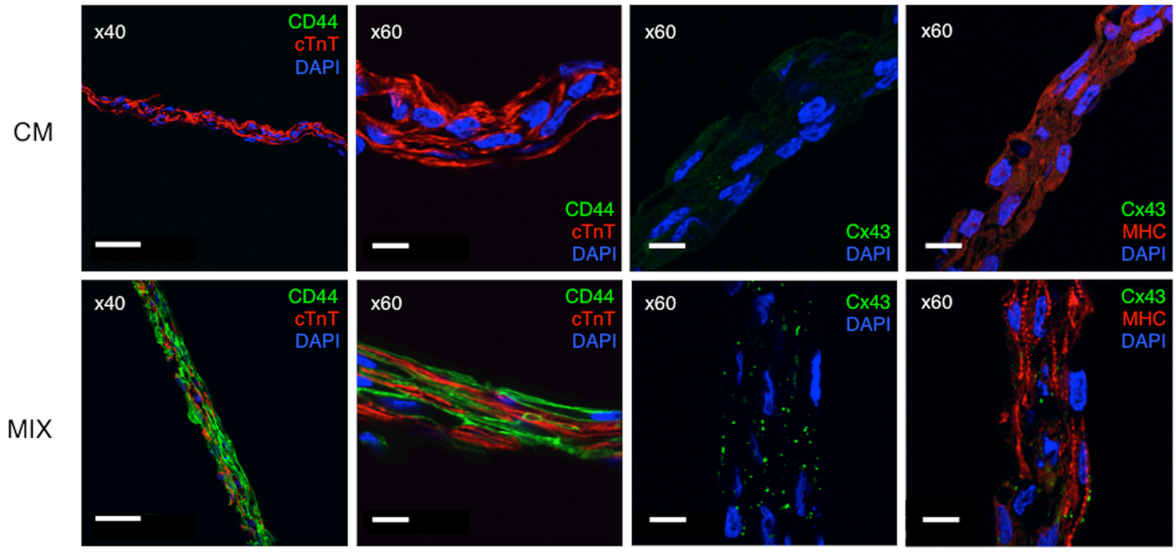

Cell sheets containing differentiated cardiomyocytes (CM, upper panels) mainly consisted of cardiac troponin T (cTnT)-positive cells, whereas cell sheets containing a mixture of CM and mesenchymal stem cells (MIX; lower panels) consisted of cTnT-positive cells and CD44-positive cells (left). The expression of connexin 43 (Cx43) in the MIX sheets was higher than in the CM sheets (right). Scale bar: 50 µm (left) and 10 µm (middles, right).

SUPPLEMENTAL TABLE

Table S1. Lists of Primers Used in This Study.

Gene Name	Primers	
CDH2	TaqMan	Hs00983056_m1
RYR2	TaqMan	Hs00181461_m1
ATP2A2	TaqMan	Hs00544877_m1
CACNA1C	TaqMan	Hs00167681_m1
KCND3	TaqMan	Hs00542597_m1
KCNJ2	TaqMan	Hs01876357_s1
SCN5A	TaqMan	Hs00165693_m1
HCN4	TaqMan	Hs00975492_m1
PPARGC1A	TaqMan	Hs01016719_m1
STC1	TaqMan	Hs00174970_m1
KDR	TaqMan	Hs00911700_m1
GJA1	TaqMan	Hs00748445_s1
β-actin (rat)	Europhins	1247000
GATA4	SYBR	F: 5'-AGGCCTCTTGCAATGCGGA-3'<br>R: 5'-CTGGTGGTGGCGTTGCTGG-3'
NKX2.5	SYBR	F: 5'-ACCTCAACAGCTCCCTGACTC-3'<br>R: 5'-ATAATCGCCGCCACAAACTCTCC-3'
MYH6	SYBR	F: 5'-TCAGCTGGAGGCCAAAAGTAAAGGA-3'<br>R: 5'-TTCTTGAGCTCTGAGCACTCGTCT-3'
MYH7	SYBR	F: 5'-TCGTGCCTGATGACAAACAGGAGT-3'<br>R: 5'-ATACTCGGTCTCGGCAGTGACTTT-3'
MYL2	SYBR	F: 5'-TGTCCCTACCTTGTCTGTTAGCCA-3'<br>R: 5'-ATTGGAACATGGCCTCTGGATGGA-3'
ALU	SYBR	F: 5'-GTCAGGAGATCGAGACCATCCC-3'<br>R: 5'-TCCTGCCTCAGCCTCCCAAG-3'
NADH	SYBR	F: 5'-ATACCCATGGCCAACCTCCT-3'<br>R: 5'-CGTCAGCTCGTGTTGTGAAA-3'
COX3	SYBR	F: 5'-ATGACCCACCAATCACATGC-3'<br>R: 5'-ATCACATGGCTAGGCCGGAG-3'

Table S2. Lists of Primary and Secondary Antibodies Used in This Study.

Antibody	Source
**For Immunocytochemistry and Immunohistochemistry**	
cTnT	Santa Cruz Biotechnology, sc-20025
MHC	Invitrogen, MA183347
Hoechst 33342	Dojindo, H342
Cx43	Sigma, C6219
N-cadherin	Abcam, ab12221
RNA cargo	Invitrogen, S32703
BODIPY™ TR Ceramide	Invitrogen, D7540
Phalloidin	Invitrogen, A34055
hTnT	Abcam, ab91605
HNA	Chemicon, MAB1281
IB4	Invitrogen, l32450
DAPI	Vector, H-1200
vWF	DAKO, A0082
CD44	Abcam, ab15107
Mouse IgG (H+L)	Life Technologies, A11001
Mouse IgG (H+L)	Life Technologies, A21422
Rabbit IgG (H+L)	Life Technologies, A11008
Rabbit IgG (H+L)	Life Technologies, A21428
**For Western Blotting**	
CD63	Invitrogen, 10628D
MHC-α	Abcam, ab185967
MHC-β	Abcam, ab172967
GAPDH	Abcam, ab8245
Beta Actin	Abcam, ab8224
Mouse IgG	GE, NA934-1ML
Rabbit IgG	GE, NA931-1ML
**For Flow Cytometry**	
Cardiac TnT	Santa Cruz Biotechnology, sc-20025
Isotype Control	Santa Cruz Biotechnology, sc-2025
Mouse IgG	Life Technologies, A11011
**For Immunoelectron Microscopy**	
CD63	Invitrogen, 10628D
Mouse IgG	Abcam, ab39619

cTnT: cardiac troponin T, hTnT: human troponin T, MHC: myosin heavy chain, Cx43: Connexin-43, HNA: human nuclear antigen, IB4: isolectin B4, DAPI: 4',6-diamidino-2-phenylindole, vWF: von Willebrand factor

Table S3. Microarray Analysis of MicroRNAs in hiPSC-CMs or Exosomes in Culture Media.

CM: differentiated cardiomyocytes derived from human induced pluripotent stem cells, MSC: human mesenchymal stem cells, SF: soluble factors secreted from human mesenchymal stem cells.

Table S4. Target Prediction with Gene Expression from Identified MicroRNAs in Exosomes.

GO: Gene Ontology, KEGG: Kyoto Encyclopedia of Genes and Genomes.

Table S5. Proteomics of MSC Exosomes.

GO: Gene Ontology, KEGG: Kyoto Encyclopedia of Genes and Genomes

SUPPLEMENTAL MOVIE

Movie S1. Representative Video of hiPSC-CMs in the CM Group without Color.

Movie S2. Representative Video of hiPSC-CMs in the CM Group with Color.
Red and blue represent a high and low velocity, respectively.

Movie S3. Representative Video of hiPSC-CMs in the CM+SF Group without Color.

Movie S4. Representative Video of hiPSC-CMs in the CM+SF Group with Color.
Red and blue represent a high and low velocity, respectively.

SUPPLEMENTAL MATERIALS AND METHODS

Cardiac Differentiation of Human Induced Pluripotent Stem Cells (hiPSCs)

hiPSC cell line 253G1, which was established in CiRA using the four Yamanaka factors, was used in this study.[1] Undifferentiated hiPSCs were expanded and maintained on SNL feeder cell layers in primate embryonic stem (ES) cell media (ReproCell, Tokyo, Japan) supplemented with 4 ng/ml basic fibroblast growth factor (bFGF) (Wako, Osaka, Japan). Cardiomyogenic differentiation was induced using a bioreactor system as previously reported.[2] Undifferentiated hiPSCs were detached and dissociated into single cells by 5 min incubation with Accumax (Innovative Cell Technologies, San Diego, CA, USA). The cells were then allowed to form embryoid bodies through suspension in modified Tenneille Serum Replacer 1 media (STEMCELL Technologies, Vancouver, Canada) supplemented with 10 µM Y-27632 (Wako) in bioreactors for 2 days (Figure 1a). On day 2, the media were changed to StemPro 34 media (Thermo Fisher Scientific, Waltham, MA, USA) supplemented with 0.5 ng/ml bone morphogenetic protein 4 (BMP4) (R&D Systems, Minneapolis, MN, USA); on day 3, media including human recombinant activin A (R&D Systems), BMP4, and basic fibroblast growth factor (bFGF) were added to the bioreactors. The final concentrations were as follows: activin A, 3 ng/ml; BMP4, 10 ng/ml; and bFGF, 5 ng/ml. On day 6, media including IWP-3, a Wnt inhibitor (Stemgent, Cambridge, MA, USA) were added at a final concentration of 4 µM IWP-3. On day 10, the media were changed to Stem Pro 34 supplemented with 5 ng/ml vascular endothelial growth factor (VEGF; R&D Systems) and 10 ng/ml bFGF. On day 16, embryoid bodies were dissociated using Accumax (Innovative Cell Technologies) for 10 min, and the number of dissociated cells was counted by Countes (Thermo Fisher Scientific).

hiPSC-derived cardiomyocytes (hiPSC-CMs) were cultured on new dishes with the same number of hMSCs (CM+MSC) or without hMSCs (CM) for three days after differentiation in Dulbecco's modified Eagle's medium (DMEM) high glucose (Thermo Fisher Scientific). To assess the effects of hMSC-secreted soluble factors, hiPSC-CMs and hMSCs were also co-cultured without direct cell-cell contact using Transwell inserts (3.0-µm pore polycarbonate membrane, Corning Inc., Armonk, NY, USA) for 3 days; hMSCs were removed before assay performance (CM+SF). As the wells in 96-well plates were too small to culture equivalent hMSC numbers, 40,000 hiPSC-CMs were co-cultured therein with 20,000 (CM+SF 50%) or 10,000 hMSCs (CM+SF 25%). For all other experiments, hiPSC-CMs were co-cultured with equivalent hMSC numbers per plate.

Flow Cytometry

Cardiomyocytes derived from human induced pluripotent stem cells (hiPSC-CMs) were dissociated with 0.25% trypsin-EDTA (Thermo Fisher Scientific), fixed with CytoFix fixation buffer (Becton Dickinson, Franklin Lakes, NJ, USA) for 20 min, permeabilized with Perm/Wash buffer (Becton Dickinson) at room temperature for 10 min, and then incubated with human anti-troponin T (TnT) antibody (Santa Cruz Biotechnology, Dallas, TX, USA) for 30 min. The labeled cells were washed with Perm/Wash buffer prior to incubation with the secondary antibody at room temperature for 30 min, and then assayed using a FACS Canto II (Becton Dickinson). The hiPSC-CMs in the CM and CM+SF groups were used in this experiment and the hiPSC-CMs in the CM+MSC group were excluded, because co-existence with hMSCs made an accurate measurement in this experiment impossible.

Quantitative Real-Time PCR (RT-qPCR)

Total RNA from hiPSC-CMs *in vitro* or cardiac tissue after cell sheet transplantation *in vivo* was isolated using the PureLink RNA Mini Kit (Thermo Fisher Scientific) or RNeasy Fibrous Tissue Mini Kit (Qiagen, Hilden, Germany), respectively. RNA was reverse transcribed to cDNA using the SuperScript III reverse transcription kit (Thermo Fisher Scientific). RT-qPCR was performed using the Viia7 Real-Time PCR system (Thermo Fisher Scientific) in triplicate for each sample with TaqMan (Thermo Fisher Scientific) or SYBR green (Thermo Fisher Scientific) probes. The samples were normalized against the housekeeping gene glyceraldehyde-3-phosphate dehydrogenase (*GAPDH*). The hiPSC-CMs in the CM and CM+SF groups were used in this experiment and the hiPSC-CMs in the CM+MSC group were excluded, because co-existence with hMSCs made an accurate measurement in this experiment impossible.

For quantification of mitochondrial damage, RT-qPCR was performed on mitochondrial genes including human cytochrome C oxidase subunit III (*COX3*) and human *NADH* dehydrogenase using culture media in the CM, CM+MSC, and CM+SF groups.

For quantification of engraftment, RT-qPCR for genomic DNA was performed 4 weeks after cell sheet transplantation using the Viia7 Real-Time PCR system in triplicate for each sample.[3] Genomic DNA was extracted from the whole LV using the PureLink Genomic DNA Mini Kit (Thermo Fisher Scientific). The samples were normalized to the rat housekeeping gene *β-actin*.

The primers used for all PCR analyses can be found in Table SI.

Western Blotting

Total protein was acquired from cardiomyocytes with or without co-culture and subjected to sodium dodecyl sulfate-polyacrylamide gel electrophoresis. After blocking with blocking one (Nakalai Tesque, Osaka, Japan), the membranes were incubated with anti-myosin heavy chain alpha (MHC-α) rabbit monoclonal antibody, anti-MHC-β rabbit monoclonal antibody, or anti-GAPDH mouse monoclonal antibody (Abcam, Cambridge, UK) overnight with shaking at 4 °C. After incubation with anti-rabbit or anti-mouse horseradish peroxidase-coupled secondary antibody (Santa Cruz Biotechnology), bands were visualized using Amersham ECL Prime Western Blotting Detection Reagent (GE Healthcare, Little Chalfont, UK) and quantified using the ChemiDoc MP Imaging System (Bio-Rad Laboratories, Hercules, CA, USA). The hiPSC-CMs in the CM and CM+SF groups were used in this experiment and the hiPSC-CMs in the CM+MSC group were excluded, because co-existence with hMSCs made an accurate measurement in this experiment impossible.
Exosomes isolated from the supernatant of hMSCs were examined by western blotting using anti-CD63 antibody (Thermo Fisher Scientific) with the same procedures explained above.

Immunocytochemistry and Immunohistochemistry Analysis

Dissociated single cells or harvested hearts were fixed with 4% paraformaldehyde and labeled with primary antibodies, followed by incubation with fluorescence-conjugated secondary antibodies, counterstaining with 4', 6-Diamidino-2-phenylindole (DAPI) (Vector Laboratories, Burlingame, CA, USA) or Hoechst33258 (Dojindo, Kumamoto, Japan), and finally analysis by confocal microscopy (FV1200 or SD-OSR, Olympus, Tokyo, Japan). The labeled cells were captured based on their fluorescence intensity. A list of the antibodies used can be found in Supplemental Table SI. The hiPSC-CMs in the CM, CM+MSC, and CM+SF groups were used in this experiment.

Electron Microscopy

hiPSC-CMs were fixed overnight in 1/2 strength Karnovsky's (2% paraformaldehyde/2.5% glutaraldehyde buffered with 0.2 M cacodylate) and post-fixed in 2% OsO_4 buffer. After dehydration, cells were embedded in Quetol 812 (Nissin EM, Tokyo, Japan), sectioned into 70-nm slices, and stained with uranyl acetate for 2 h and lead citrate for 5 min. The samples were imaged using an electron microscope (Hitachi H-7500; Hitachi, Tokyo, Japan) set to 80 kV. The hiPSC-CMs in the CM, CM+MSC, and CM+SF groups were used in this experiment.

Cell Motility Analysis

Beating cells were monitored at a rate of 150 Hz for 6 s at 37 °C with a high-speed camera-based motion analysis system (SI8000 View; Sony, Tokyo, Japan). The beating area, contraction velocity, relaxation velocity, and acceleration were measured using SI8000C Analyzer (Sony). The hiPSC-CMs in the CM and CM+SF groups were used in this experiment and the hiPSC-CMs in the CM+MSC group were excluded, because co-existence with hMSCs made an accurate measurement in this experiment impossible.

Ca^{2+} Transient Measurement

Ca^{2+} transient measurement was performed using 96-well plates. After hiPSC-CMs were washed with phosphate buffered saline (PBS), cells were loaded with 5 µM Fluo-8 regents (AAT Bioquest, Sunnyvale, CA, USA) in serum-free minimal essential media (MEM) at 37 °C for 30 min. Intracellular fluorescence was recorded with or without pacing at 0.5–3 Hz at 37 °C using an FDSS/µCELL system (Hamamatsu Photonics, Hamamatsu, Japan). Data were analyzed using FDSS software U8524-12 (Hamamatsu Photonics) to obtain beating rate, peak ratio, rising slope, and peak width duration. Fluorescence intensity was described using relative fluorescence units (rfu) in this study. The hiPSC-CMs in the CM, CM+SF 25%, and CM+SF 50% groups were used in this experiment because 96-well plates should be used for this experiment. The hiPSC-CMs in the CM+MSC group were excluded, because co-existence with hMSCs made an accurate measurement in this experiment impossible.

Mitochondrial Function Assay

The Seahorse XF96 extracellular flux analyzer was used to assess mitochondrial function. Plates were pre-treated with bovine serum albumin; at 16 days after differentiation, the cells were seeded onto the plates at a density of 40,000 cells per XF96 well. The cells were cultured at 37 °C for 3 days in the Seahorse plates before analysis. Culture medium was exchanged for a basal medium (XF assay medium supplemented with 25 mM glucose and 1 mM sodium pyruvate) 1 h before the assay and for the duration of the measurement of oxygen consumption rate (OCR) and extracellular acidification rate (ECAR). Substrates and selective inhibitors were injected during the measurements to achieve final concentrations of 25 mM glucose, 2.5 µM oligomycin, 1 µM carbonyl cyanide-p-trifluoromethoxyphenylhydrazone (FCCP), 2.5 µM rotenone, and 2.5 µM antimycin A. The basal respiration rate was defined as the average values of OCR measured from time point 1 to 4 (0–21 min) during the experiments. Adenosine triphosphate (ATP) production was defined as the difference in OCR between the basal respiration and the OCR value after 2.5 µM oligomycin injection. Spare respiratory capacity was defined as the difference in OCR

between the basal respiration and the OCR value after 1 μM FCCP injection. In addition, the metabolic potential was evaluated by calculating the stressed OCR and the stressed ECAR. The stressed OCR was defined as the ratio of the OCR under stressed conditions with 1 μM FCCP to the OCR under normal conditions. We measured the ECAR under normal and stressed conditions with 2.5 μM oligomycin; the stressed ECAR was defined as the ratio of the ECAR under stressed conditions to the ECAR under normal conditions. The hiPSC-CMs in the CM, CM+SF 25%, and CM+SF 50% groups were used in this experiment because 96-well plates should be used for this experiment. The hiPSC-CMs in the CM+MSC group were excluded because co-existence with hMSCs made an accurate measurement in this experiment impossible.

Reactive Oxygen Species (ROS) Measurement

The levels of intracellular ROS were analyzed using the OxiSelect Intracellular ROS Assay Kit (Cell Biolabs, San Diego, CA, USA) according to the manufacturer's instructions after hiPSC-CMs were cultured with or without hMSCs in 24-well plates for 3 days. Briefly, the cells were washed three times with PBS and 200 μl 1× 2′,7′-dichlorodihydrofluorescin diacetate (DCFH-DA) solution was added to the cells, which were incubated at 37 °C for 30 min in light. After washing three times with PBS, the cells were incubated in DMEM with or without 2 μM H_2O_2 for 60 min. The absorbance of the cells was measured at 530 nm on a microplate reader (PowerWave HT; BioTek, Beijing, China). The cells in the CM, CM+MSC, and CM+SF groups were used in this experiment.

Protein Analysis

Enzyme-linked immunosorbent assay (ELISA) kits were used to measure proteins such as hepatocyte growth factor (HGF), stromal cell-derived factor 1 (SDF-1), and vascular endothelial growth factor (VEGF; R&D Systems) secreted from the cultured cells, according to the manufacturers' instructions. The concentration of other proteins secreted from the cultured cells was measured using the Bio-Plex suspension array system (27-plex; Bio-Rad Laboratories) according to the manufacturer's instructions. The media in the CM and CM+MSC groups were used in this experiment.

Recombinant Proteins and Inhibitors

To investigate the impact of each cytokine derived from hMSCs on the maturity of hiPSC-CMs, we used recombinant proteins and inhibitors in several experiments. Recombinant VEGF, recombinant SDF-1, recombinant bFGF, and recombinant

granulocyte-macrophage colony-stimulating factor (GM-CSF, all from R&D Systems) were purchased as recombinant proteins. Anti-VEGF neutralizing antibodies, anti-SDF-1 neutralizing antibodies, anti-bFGF neutralizing antibodies, and anti-GM-CSF neutralizing antibodies (all from R&D Systems) were purchased as blocking antibodies. GW4869 (Sigma-Aldrich, St. Louis, MO, USA) was purchased as an exosome secretion blocker.

Immunoelectron Microscopy

Preparation of exosomes for transmission electron microscopy (TEM) was performed as described previously by Lässer *et al*.[4] After isolation of the exosomes, they were pre-fixed using 2% paraformaldehyde for 10 min. Then, the samples were immunostained with anti-CD63 antibody (Thermo Fisher Scientific) for 40 min and 10 nm-gold labeled secondary antibodies (Abcam) for 40 min. After washing the grid, we post-fixed the samples by incubating the grid with 2.5% glutaraldehyde for 10 min. We contrasted the sample with 2% uranyl acetate for 15 min. After incubating the grid for 10 min with 0.13% methyl cellulose and 0.4% uranyl acetate, the samples were examined under an electron microscope (Hitachi H-7500; Hitachi, Tokyo, Japan). The antibodies used in this study can be found in Table SII.

Particle Size Analysis

Size distribution analysis of the exosomes was performed using the qNano system (Izon Science, Christchurch, New Zealand). After diluting 5 μl of samples in 45 μl of dilution buffer (100 mM KCl (Wako), 10 mM Tris (hydroxymethyl) aminomethane (Wako), 3 mM ethylenediaminetetraacetic acid (Nakalai Tesque), and 0.01% v/v Triton X-100 (MP Biomedicals, Santa Ana, CA, USA)), the size of the exosomes was measured using NP100 nanopores and CPC100B calibration particles and analyzed using Izon Control Suite 3.2 software according to the manufacturer's instructions.

Microarray for MicroRNAs

MicroRNAs were extracted from 1 $\times 10^6$ hiPSC-CMs or exosomes using the mirVana™ miRNA Isolation Kit (Thermo Fisher Scientific) according to the manufacturer's instructions. The concentration and purity of the extracted RNA were determined using the ND-1000 Spectrophotometer (NanoDrop). Cellular RNA (200 ng) or exosomal RNA (50 ng) were retrotranscribed and pre-amplified according to the manufacturer's instructions (Thermo Fisher Scientific). Pre-amplified products were loaded onto the TaqMan Array Human MicroRNA A Cards v2.0 (Thermo Fisher Scientific). PCR was then performed using Viia7 Real-Time PCR system. The results of PCR were normalized against *RNU44*, which was

selected as an endogenous control.

Liquid Chromatography Tandem-Mass Spectrometry (LC-MS/MS) Analysis

LC-MS/MS was performed as described previously,[5, 6] with slight modifications. Peptides were extracted from exosomes using MPEX PTS Reagents (GL Sciences Inc., Tokyo, Japan) and were separated through 250-min gradient elution at a flow rate of 250 nl/min with the UltiMate 3000 RSLCnano System (Thermo Fisher Scientific), which was directly interfaced with the Q Exactive Hybrid Quadrupole-Orbitrap Mass Spectrometer (Thermo Fisher Scientific). The Acclaim PepMap RSLC column was used. Mobile phase A consisted of water with 0.1% formic acid, and mobile phase B consisted of methanol with 0.1% formic acid. The Q Exactive Mass Spectrometer was operated in the data-dependent acquisition mode using Xcalibur 2.1.2 software and there was a single full-scan mass spectrum in the orbitrap (350–1500 m/z, 70,000 resolution), followed by 15 data-dependent MS/MS scans at 27% normalized collision energy.

Protein identification was performed using Proteome Discoverer 1.4 software (Thermo Fisher Scientific). The spectra were extracted from raw MS data files and searched against the SwissProt reviewed human proteome database. Precursor Mass Tolerance was 10 ppm, Fragment Mass Tolerance was 0.1 Da and a maximum of two missed cleavages were allowed. Carbamidomethylation (on C) was set as static modification, and oxidation (on M) was set as dynamic modification. Protein identification was considered valid if at least one peptide was statistically significant (with a false discovery rate (FDR) of 1%). Default values were used for all other parameters not mentioned above.

Gene Ontology (GO) Analysis/Pathway Analysis

The software MirPath v.3 from the DIANA tools website (http://snf-515788.vm.okeanos.grnet.gr/) was used to identify potential miRNA target genes and pathways in our study. microT-CDS was used to predict the potential target genes and demonstrate possible relationships between the databases. Pathway analysis was performed to determine the involvement of co-expressed genes in different biological pathways according to the Kyoto Encyclopedia of Genes and Genomes (KEGG). GO analysis was used to investigate the pathways associated with biological processes, cellular components, and specific molecular functions corresponding to the target genes of miRNAs identified by the software microT-CDS. Using the results of LC-MS/MS analysis, functional enrichment analysis was conducted using DAVID Bioinformatics Resources 6.8 online (https://david.ncifcrf.gov/) for the GO and KEGG pathway analyses.

Echocardiography

Echocardiography (Vivid i; GE Healthcare) was performed on rats under general anesthesia using 1% isoflurane just before, 1, 2, and 4 weeks after the treatment procedure. The left ventricular end-systolic dimension (LVESD) and end-diastolic dimension (LVEDD) were obtained from M-mode tracings at the midpapillary level. The left ventricular ejection fraction (LVEF) was calculated as follows: LVEF (%) = $[(LVEDD^3 - LVESD^3) / LVEDD^3]$[4].

Cardiac Catheterization

To assess systolic and diastolic cardiac function, cardiac catheterization was performed on rats under general anesthesia using 1% isoflurane, 4 weeks after the treatment procedure. A MicroTip catheter transducer (SPR-671; Millar Instruments Inc., Houston, TX, USA) and conductance catheters (Unique Medical Co, Osaka, Japan) were placed longitudinally in the left ventricle (LV) from the apex and connected to an Integral 3-signal conditioner-processor (Unique Medical Co.). End-systolic elastance and end-diastolic elastance were determined by transiently compressing the inferior vena cava. Data were recorded as a series of pressure–volume loops, which were analyzed using Integral 3 software (Unique Medical Co.). The maximal and minimal rates of change in LV pressure (dP/dt max and dP/dt min, respectively) were obtained from steady-state beats. After the hemodynamic assessment, the rats were sacrificed through anesthetic overdose and the hearts were removed for further biochemical and histological analyses.

Statistical Analysis

Data are presented as the means with standard error for continuous variables. Continuous variables were examined using the Student *t*-test. The one-way ANOVA test was used to compare the values between more than two groups. When the one-way ANOVA test was significant, group differences were compared using the post hoc Tukey's HSD test. Statistical analyses were performed using JMP®13 (SAS Institute Inc., Cary, NC). Statistical significance was defined as $P < 0.05$.

References

1. Takahashi, K, Tanabe, K, Ohnuki, M, Narita, M, Ichisaka, T, Tomoda, K*, et al.* (2007). Induction of pluripotent stem cells from adult human fibroblasts by defined factors. *Cell* **131**: 861-872.
2. Matsuura, K, Wada, M, Shimizu, T, Haraguchi, Y, Sato, F, Sugiyama, K*, et al.* (2012). Creation of human cardiac cell sheets using pluripotent stem cells. *Biochem Biophys Res Commun* **425**: 321-327.
3. Lee, WY, Wei, HJ, Lin, WW, Yeh, YC, Hwang, SM, Wang, JJ*, et al.* (2011). Enhancement of cell retention and functional benefits in myocardial infarction using human amniotic-fluid stem-cell bodies enriched with endogenous ECM. *Biomaterials* **32**: 5558-5567.
4. Lasser, C, Eldh, M, and Lotvall, J (2012). Isolation and characterization of RNA-containing exosomes. *J Vis Exp*: e3037.
5. Xu, L, Gao, Y, Chen, Y, Xiao, Y, He, Q, Qiu, H*, et al.* (2016). Quantitative proteomics reveals that distant recurrence-associated protein R-Ras and Transgelin predict post-surgical survival in patients with Stage III colorectal cancer. *Oncotarget* **7**: 43868-43893.
6. Jin, L, Huo, Y, Zheng, Z, Jiang, X, Deng, H, Chen, Y*, et al.* (2014). Down-regulation of Ras-related protein Rab 5C-dependent endocytosis and glycolysis in cisplatin-resistant ovarian cancer cell lines. *Mol Cell Proteomics.* 2014;13(11): 3138-3151.
